# A Comprehensive Experimental and Theoretical Investigation of the Antioxidant Properties of Hispidin and Isohispidin

**DOI:** 10.1021/acs.joc.4c02837

**Published:** 2025-02-25

**Authors:** Houssem Boulebd, Imene Amine Khodja, Khedidja Benarous, Marcin Mą̨czyński, Maciej Spiegel

**Affiliations:** † Laboratory of Synthesis of Molecules with Biological Interest, University of Frères Mentouri Constantine 1, Constantine 25017, Algeria; ‡ Fundamental Sciences Laboratory, Amar Telidji University, Laghouat 03000, Algeria; § Department of Organic Chemistry and Pharmaceutical Technology, Faculty of Pharmacy, Wroclaw Medical University, Borowska 211A, Wroclaw 50-556, Poland; ⊥ Laboratory of Applied Sciences and Didactics, Higher Normal School of Laghouat, Laghouat 03000, Algeria

## Abstract

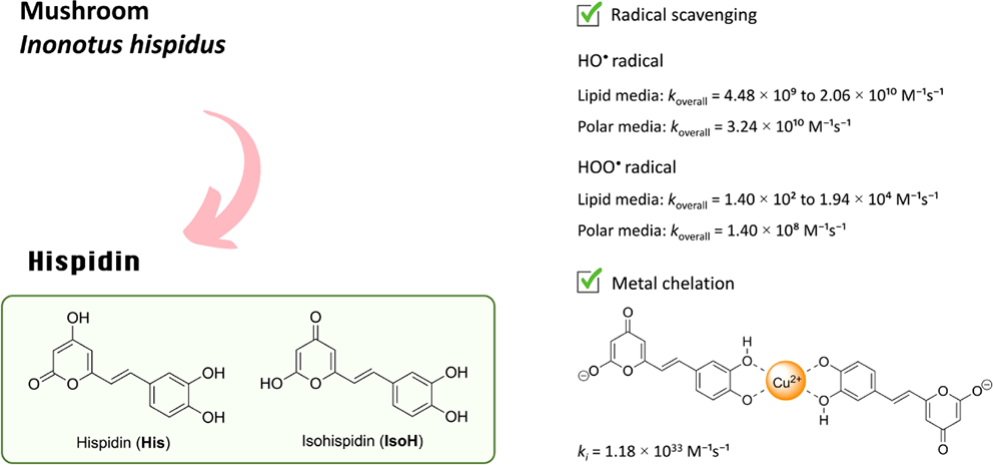

This study provides a comprehensive analysis of the antioxidant activity of hispidin (**His**) and its tautomer isohispidin (**IsoH**) using DFT calculations, corroborated by experimental data. Under physiological conditions, both tautomers demonstrated significant scavenging capacity for the HO^•^ radical, with *k*_overall_ ranging from 4.48 × 10^9^ to 2.06 × 10^10^ M^–1^ s^–1^ in lipid media and 3.24 × 10^10^ M^–1^ s^–1^ in water. Mechanistic analysis revealed that the radical adduct formation (RAF) mechanism is dominant in lipid environments, whereas both RAF and single electron transfer (SET) operate nonselectively in water. Hispidin also exhibited strong scavenging capacity for the HOO^•^ radical in water (*k*_overall_ = 1.40 × 10^8^ M^–1^ s^–1^), but its reactivity in lipid environments was comparatively lower, with *k*_overall_ of 1.40 × 10^2^ M^–1^ s^–1^ for **His** and 1.94 × 10^4^ M^–1^ s^–1^ for **IsoH**. The *f*-HAT mechanism was identified as the predominant pathway in lipid media, while both *f*-HAT and SET contribute to HOO^•^ scavenging in water. Additionally, hispidin demonstrated a strong ability to chelate copper(II) ions, effectively inhibiting HO^•^ radical formation via the Fenton reaction. The theoretical results align well with the experimental data from the DPPH and ABTS assays, indicating that hispidin is a potent antioxidant under physiological conditions.

## 1 Introduction

Different species of the genera *Inonotus* and *Phellinus* (family *Hymenochaetaceae*) synthesize hispidin, a yellow phenolic compound.^1^ In the past, these wood-decay fungi were used as traditional remedies in Algeria, Western Siberia, and Russia to treat various serious diseases, including cancer, liver, gastric, and heart conditions.^2−4^ Several in vitro studies^2,5−10^ have demonstrated their effectiveness against a range of conditions, including antilipase, antiobesity, antigout, anticancer, anti-inflammatory, as well as antiviral activity against COVID-19 and influenza A and B viruses. This mushroom, a facultative saprophyte (brown basidiomycete), has been observed as a parasite on various broadleaf trees in China and Europe. These trees include *Ulmus campestris*, *Sorbus aucuparia*, *Acer saccharum*, Euphrates poplar (*Populus euphratica*), mulberry (*Morus alba L*.), and Manchurian ash (*Fraxinus mandshurica*).^9^ In our traditional medicine, this mushroom is called “Sorret Elbtoum” and produces yellow fruiting bodies, while in ancient Chinese medical texts, it is referred to as “Sanghuang″, “Meshimakobu” in Japan and “Sanghwang” in South Korea.^9^ This mushroom has a long history of therapeutic use and significant economic value in Algeria and Southeast Asia.^9^

**Figure 1:**
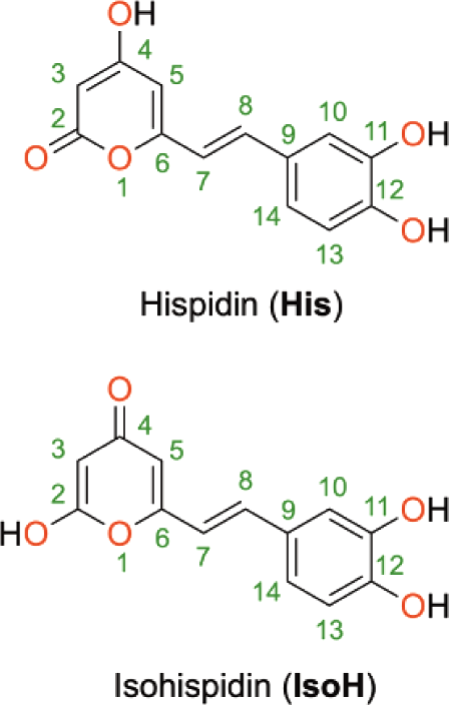
Atomic numbering and molecular structure of hispidin (**His**) and isohispidin (**IsoH**).

Hispidin is a phenolic compound comprising two aromatic rings: 4-hydroxy-pyran-2-one (**His**), which tautomerizes to 2-hydroxy-pyran-4-one (**IsoH**), and 4,5-dihydroxy-phenyl, connected by a double bond (Figure 1). Although the phenolic structure of hispidin suggests potential for antioxidant activity, this property has not been extensively investigated or rigorously established. Only a few studies have investigated its antioxidant activity. Han et al. examined the antiradical activity of hispidin and its derivatives, isolated from the fruiting body of *Phaeolus schweinitzii*, using three methods: the DPPH scavenging assay, the total antioxidant capacity assay, and the lipid peroxidation assay.^11^ The substance showed significant antioxidant activity in these assays. In the DPPH scavenging assay, it presented an IC_50_ value of 58.8 μM, which is lower than that of other compounds. In the total antioxidant capacity assay, hispidin exhibited higher antioxidant capacity. Additionally, in the lipid peroxidation assay, it effectively inhibited lipid peroxidation in mouse liver homogenate, leading to a significant decrease in MDA content, a key aldehyde product of lipid peroxidation. Furthermore, it has been shown that hispidin, isolated from mushrooms such as *Phellinus linteus* and *Inonotus hispidus*, has potent scavenging capacity for DPPH and superoxide anions, comparable to α-tocopherol and BHT. However, its scavenging capacity against hydroperoxyl radicals is lower than that of BHA.^12,13^

These experimental studies highlight the remarkable antioxidant potential of the polyphenol; however, none have explored the mechanism by which it is exerted. The study of the reaction mechanisms of natural antioxidants could provide crucial information on the structure–activity relationship of antioxidants, paving the way for the discovery of new potent antioxidants. In this context, only one study has been conducted on the antioxidant mechanism of **His**. This study examined the antiradical mechanism using DFT calculations at the theoretical level B3P86/6–31+G(d,p).^14^ Although this work revealed useful information on the antioxidant mechanism of the compound, particularly the role of OH groups and spin density delocalization, its tautomer and important aspects of the mechanism remain to be examined, such as the influence of the lipid physiological environment, the dissociation of OH groups at physiological pH, RAF (radical adduct formation) and SPLET (sequential proton loss electron transfer) mechanisms, metal ion chelation capacity, and pro-oxidant properties. Consequently, the antioxidant activity of hispidin requires further investigation.

In this study, we conduct a combined experimental and theoretical investigation into the antioxidant activity and underlying mechanism of hispidin isolated from the mushroom *Inonotus hispidus*.^2,6^ The antiradical capacity of this molecule was first examined using the DPPH and ABTS assays and compared with that of the standard Trolox. Subsequently, the thermodynamics and kinetics of all antioxidant mechanisms were examined for all potential positions toward both hydroxyl and hydroperoxyl radicals. Several factors were considered, including the influence of lipid and polar physiological conditions and the influence of physiological pH. Additionally, the capacity of the phytochemical to chelate Cu(II) ions, involved in the formation of hydroperoxyl ions via the Fenton reaction, was also considered under polar physiological conditions. The pro-oxidant activity of the complexes resulting from chelation with Cu(II) was also evaluated.

## 2 Material and Methods

### 2.1 Extraction and Isolation of Hispidin

The fruit body of the mushroom *Inonotus hispidus* was obtained in October 2022 from a herborist in Laghouat City, south of Algeria. This fungus was identified by KB and a sample is deposited in by Bernard Duhem, Muséum National d’Histoire Naturelle, Laboratoire de Cryptogamie, 12 Buffon Street, 75231 Paris, France.^2^ The extraction, isolation, and characterization of hispidin were carried out according to the same protocol cited in our previous works.^2,6^

### 2.2 DPPH and ABTS Assays

#### 2.2.1 DPPH Assay

The presence of a hydrogen-donating antioxidant decreases the absorption of the DPPH solution at 517 nm, resulting in a loss of its characteristic deep violet color. The absorption at 517 nm vanishes proportionally to the degree of DPPH reduction by the antioxidant. The remaining nonreduced DPPH, measured after 30 min, inversely reflects the radical scavenging activity. We followed the same experimental protocol described by Benarous et al. (2015)^2^ with slight modifications. Equal volumes of a 150 μM DPPH solution and hispidin solutions prepared at varying concentrations were added to the wells of a 96-well microplate. The plate was incubated at ambient temperature for 30 min. Subsequently, a Thermo Fisher Scientific microplate reader was used to measure the absorbance of each well at 517 nm. All assays were performed in triplicate with a final volume of 240 μL in each well.

#### 2.2.2 ABTS Assay

The ABTS (2,2′-azinobis (3-ethylbenzothiazoline sulfonate) and peroxidation enzyme (peroxidase metmyoglobin or horseradish peroxidase) assay is a method used to evaluate antioxidant activity in plant extracts and pure molecules (natural or synthesized). For this cation radical generation, we mixed three components: 1 mL of ABTS (20 mM), 150 μL of H_2_O_2_ (1 mM), and 1 mL of buffer solution peroxidase (0.2 mg/mL) with pH 6.9, then the volume is adjusted to 100 mL. The resulted mixture is blue in color. We used a Thermo Fisher Scientific microplate reader to read the obtained absorbances at 417 nm. The assays are done in triplicates for a final mixture in each well of 240 μL (40 μL for hispidin and Trolox with different concentrations and 200 μL of ABTS cation aqueous solution).

### 2.3 Computational Details

All density functional theory (DFT) calculations in this work were performed using Gaussian 16 software.^15^ The M06-2X function coupled with the 6-311++G(d,p) basis set was used for all calculations of the radical scavenging mechanisms.^16^ Previous studies have verified the reliability of this methodology, particularly for reactions involving free radicals.^17,18^ Research on the copper-involving antioxidative activity, however, was studied under the M06 functional which is recommended for application in organometallic chemistry.^16^ The metal cation was described using SDD effective core potentials,^19^ in accordance with the methodology validated in our previous papers.^20−23^ The ground and transition states were confirmed by imaginary frequencies (0 and 1, respectively). To simulate polar and lipid physiological conditions, solvation effects of water and pentyl ethanoate, respectively, were incorporated using the SMD solvation model.^24^ The thermodynamic descriptors relating to the antioxidant mechanisms studied were calculated as indicated in previous research.^25−27^ Kinetic assessments were conducted following the QM-ORSA (quantum mechanics-based overall free radical scavenging activity test) approach.^28,29^ The rate constant (*k*) was determined using standard transition state theory (TST) and a 1 M standard state at 298.15 K, calculated according to the equation below:^30−32^
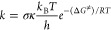


Where σ is the reaction symmetry number,^33,34^ κ represents tunneling corrections computed using the Eckart barrier,^35^*k*_B_ is the Boltzmann constant, *h* is the Planck constant, Δ*G*^≠^ is Gibbs free energy of activation. Additional methodological details are provided in Table S1.

## 3 Results and Discussion

### 3.1 Experimental Investigation of the DPPH and ABTS Radical Scavenging Capacity

To determine the antiradical potential of hispidin, we conducted an experimental study using commonly used DPPH and ABTS tests. Trolox was used as a reference, and the results are presented in Table 1. The IC_50_ value of hispidin was slightly higher than that of Trolox in the DPPH test (36.05 ± 0.08 and 30.17 ± 2.48 μM, respectively). However, in the ABTS assay, its IC_50_ was approximately 10 μM lower than that of Trolox (52.13 ± 4.30 and 64.71 ± 2.75 μM, respectively). Assuming that DPPH inhibition occurs via hydrogen transfer (HT), while ABTS inhibition occurs via electron transfer (ET),^36^ we can conclude that Trolox is more potent than the polyphenol in terms of hydrogen transfer mechanisms. However, hispidin turns out to be a better electron donor than Trolox. These results suggest that hispidin is a potent antioxidant, comparable to Trolox, and warrant further investigation of its antioxidant activity by computational methods.

**Table 1:** Antioxidant Activities of **His** and Trolox

Compound	DPPH (IC_50_ in μM)	ABTS (IC_50_ in μM)
Hispidin	36.05 ± 0.08	52.13 ± 4.30
Trolox	30.17 ± 2.48	64.71 ± 2.75

### 3.2 Theoretical Investigation of the Radical Scavenging Capacity

#### 3.2.1 Thermodynamic and Kinetic Evaluation in the Gas-Phase

In the initial phase of our investigation, we assessed the thermodynamic and kinetic properties of **His** and **IsoH** reactions with common ROS radicals, namely HO^•^ and HOO^•^, in the gas phase. Under these conditions, the polyphenols are anticipated to engage in two distinct mechanisms known as *f*-HAT (formal hydrogen transfer) and RAF (radical adduct formation), which exhibit relatively minimal dependence on environmental factors (eqs 1 and 2).^37−39^ Mechanisms involving electron transfer, such as SETPT (sequential electron transfer proton transfer) and SPLET (sequential proton loss electron transfer), are favored primarily in polar environments and were thus not considered in this preliminary analysis (eqs 3 and 4). Figure S1 depicts a schematic representation of the investigated mechanisms.
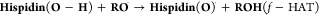
1
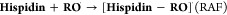
2
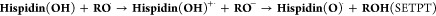
3
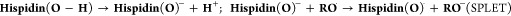
4

**Figure 2:**
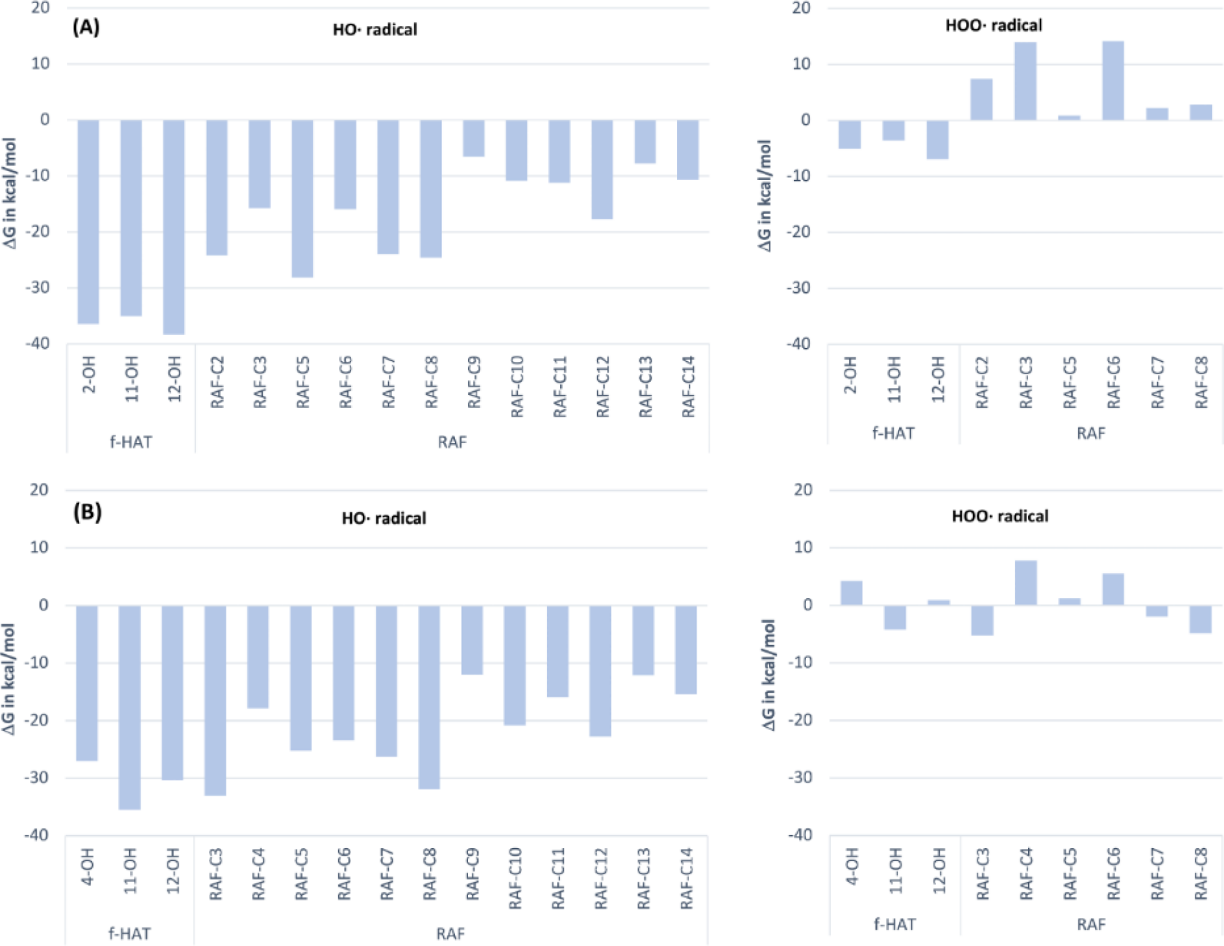
Thermodynamic evaluation of the reaction of **IsoH** (A) and **His** (B) with HO^•^ and HOO^•^ radicals in the gas phase following the *f*-HAT and RAF mechanisms.

The calculated Gibbs free energies (Δ*G*°) for the reactions between HO^•^ and HOO^•^ with **His** and **IsoH** at all potential sites are depicted in Figure 2. It is evident that all reactions involving the HO^•^ radical are thermodynamically favorable, with Δ*G*° values ranging from −12.0 to −35.5 kcal/mol for **His** and from −6.5 to −38.3 kcal/mol for **IsoH**. Reactions between **His** and the HOO^•^ radical are only favorable for the singular *f*-HAT mechanism (−4.2 kcal/mol) and three RAF pathways (Δ*G*° ranging from −1.9 to −5.2 kcal/mol). Conversly, **IsoH** is capable to attenuate HOO^•^ solely via *f*-HAT routes, the Gibbs free energies of are enclosed within −3.6 to −6.9 kcal/mol, in contrast to RAF reactions, all determined to be endergonic (Δ*G*° ranging from 0.9 to 14.1 kcal/mol). Consequently, only thermodynamically favorable reactions were considered in the following kinetic analysis.

The kinetics of the reactions between the studied polyphenols and the HO^•^ and HOO^•^ radicals were investigated, and the results are presented in Tables 2 and 3. As evidenced from the collected data, while **His** scavenging activity against HOO^•^ is linked with the *f*-HAT from 11OH and RAF from C3, C7, and C8, the propensity of hydrogen transfer outnumbers the others. The obtained individual rate constant of 2.87 × 10^–15^ cm^3^ molecule^–1^ s^–1^ actually constitutes the observable one. This specificity changes when it comes to HO^•^. Although *f*-HAT is still a major route, the branching ratios collected for RAFs implicate their contribution to the overall activity too.

**Table 2:** Calculated Kinetic Characteristics of the Reactions of **His** with HO^•^ and HOO^•^ Radicals in the Gas Phase^a^

	Mechanisms		IF^b^ (cm^–1^)	Δ*G^≠^*^c^ (kcal/mol)	*K*^d^	Γ^e^ (%)	*k*_overall_
HOO^•^	*f*-HAT	11OH	–2742.6	12.2	2.87 × 10^–15^	100	2.87 × 10^–15^
	RAF	C3	–662.1	14.4	4.79 × 10^–19^	0	
		C7	–632.0	15.9	3.52 × 10^–20^	0	
		C8	–746.9	15.5	7.58 × 10^–20^	0	
HO^•^	*f*-HAT	4OH	–515.4	0.5	4.63 × 10^–12^	10	4.52 × 10^–11^
		11OH^f^	-	-	7.28 × 10^–12^	16	
		12OH	–1731.5	4.4	2.65 × 10^–12^	6	
	RAF	C3	–144.8	–0.3	4.10 × 10^–12^	9	
		C4	–458.8	7.1	8.40 × 10^–14^	0	
		C5	–334.7	2.2	4.04 × 10^–12^	9	
		C6	–416.9	5.1	1.45 × 10^–12^	3	
		C7	–320.9	3.7	3.53 × 10^–12^	8	
		C8	–371.2	2.6	4.00 × 10^–12^	9	
		C9	–460.9	7.9	1.93 × 10^–14^	0	
		C10	–272.5	0.4	4.05 × 10^–12^	9	
		C11	–244.1	0.8	4.04 × 10^–12^	9	
		C12	–371.5	3.4	3.56 × 10^–12^	8	
		C13	–496.2	7.0	9.76 × 10^–14^	0	
		C14	–452.8	5.0	1.63 × 10^–12^	4	

a The *k* values are in cm^3^ molecule^–1^ s^–1^.

b Imaginary frequency.

c Activation free energy.

d Rate constant.

e Branching ratio.

f The relaxed scan indicates that the reaction is barrierless and the spontaneous H-transfer takes place.

**Table 3:** Calculated Kinetic Characteristics of the Reactions of **IsoH** with HO^•^ and HOO^•^ Radicals in the Gas Phase^a^

	Mechanisms		IF^b^ (cm^–1^)	Δ*G^≠^*^c^ (kcal/mol)	*K*^d^	Γ^e^ (%)	*k*_overall_
HOO^•^	*f*-HAT	2OH	–2246.62	16.5	1.00 × 10^–17^	98	1.20 × 10^–17^
		11OH	–2325.5	20.0	1.10 × 10^–19^	1	
		12OH	–2351.4	19.9	1.20 × 10^–19^	1	
HO^•^	*f*-HAT	2OH	–2603.1	7.8	1.40 × 10^–14^	0	1.05 × 10^–10^
		11OH	–2456.73	9.0	4.30 × 10^–14^	0	
		12OH	–2713.07	7.8	4.60 × 10^–14^	0	
	RAF	C2	–369.3	5.9	1.30 × 10^–11^	12	
		C3	–365.6	5.7	1.70 × 10^–11^	16	
		C5	–424.7	6.5	4.10 × 10^–12^	4	
		C6	–506.6	10.4	6.50 × 10^–15^	0	
		C7	–306.6	5.7	1.60 × 10^–11^	15	
		C8	–351.6	7.5	7.60 × 10^–13^	1	
		C9	–445.8	11.6	7.40 × 10^–16^	0	
		C10	–281.6	5.4	2.90 × 10^–11^	28	
		C11	–249.7	5.5	2.50 × 10^–11^	24	
		C12	–370.0	8.2	2.60 × 10^–13^	0	
		C13	–494.6	11.6	7.40 × 10^–16^	0	
		C14	–438.5	9.3	3.70 × 10^–14^	0	

a The *k* values are in cm^3^ molecule^–1^ s^–1^.

b Imaginary frequency.

c Activation free energy.

d Rate constant.

e Branching ratio.

In the instance of **IsoH**, the reaction with the HOO^•^ radical, hydrogen abstraction at the 2OH position, is dominant, even exclusive, with a contribution of 98%. On the other hand, the other OH groups made a negligible contribution, of the order of 2%. This observation suggests that the OH group of the pyran ring is the main site of **IsoH**’s antioxidant activity toward the HOO^•^ radical. Concerning the HO^•^ radical, the RAF mechanism is characterized by particularly favorable kinetics, accounting for almost the entire reaction (contribution of about 100%). In particular, the attacks at positions C2, C3, C7, C10, and C11 stand out as the most reactive sites, contributing to 95% of the total reactivity. The other positions show a minimal contribution in the reaction of **IsoH** with the HO^•^ radical. Analysis of the overall rate constants toward the HO^•^ and HOO^•^ radicals (1.50 × 10^–10^ and 1.20 × 10^–17^ cm^3^ molecule^–1^ s^–1^, respectively) reveals that **IsoH** is an excellent scavenger of the HO^•^ radical and a good scavenger of the HOO^•^ radical in the gas phase. It emerges that **IsoH** is a much better antiradical agent than **His**.

#### 3.2.2 Radical Scavenging Capacity in Physiological Media

**Figure 3:**
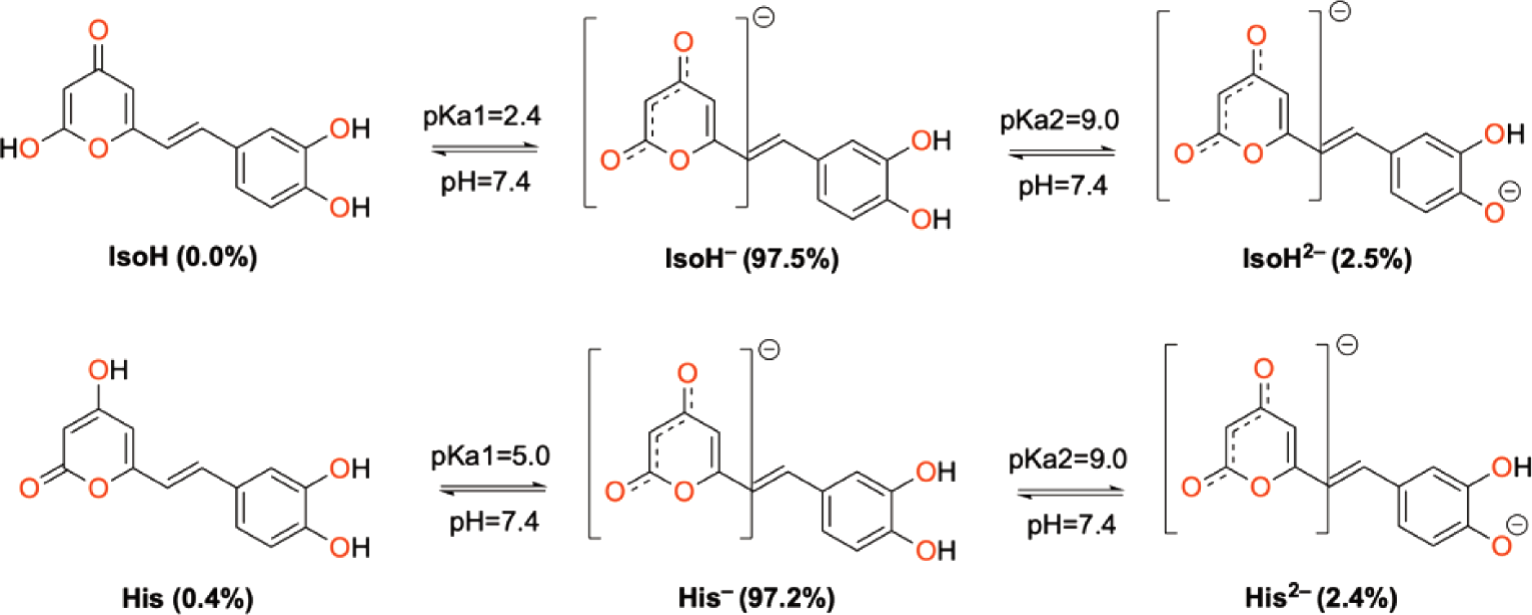
Computed p*K*_a_ values of **IsoH** and **His** and their acid–base equilibrium at physiological pH.

Given that the antiradical activity occurs in solution, the influence of the environment is a crucial factor to consider. Thus, we examined the effects of polar and lipid environments on the reaction of hispidin with HO^•^ and HOO^•^ radicals. Lipid conditions were simulated using pentylethanoate as a solvent, while polar conditions were studied in water at a physiological pH (7.4). Since hispidin is a phenolic compound subject to deprotonation at physiological pH, we first calculated its p*K*_a_ values following a previously established protocol.^40^ The obtained values as well as the equilibria in water at physiological pH are presented in Figure 3. The calculated p*K*_a_ values are 2.4 and 9.0 for **IsoH** and 5.9 and 9.0 for **His**. These indicate that the monodeprotonated state is dominant for both tautomers (97.2 to 97.5%) at physiological pH, with a small proportion (2.4–2.5%) present in the doubly deprotonated state.

It is important to consider these dissociated forms in water, as their reactivity may differ from that of the neutral form. Given these outcomes and the previously mentioned tautomeric equilibrium between two isoforms, the proton that is released upon deprotonation results in indistinguishable forms, hereafter considered within the research in water as a single entity, referred to as **His**.

**Figure 4:**
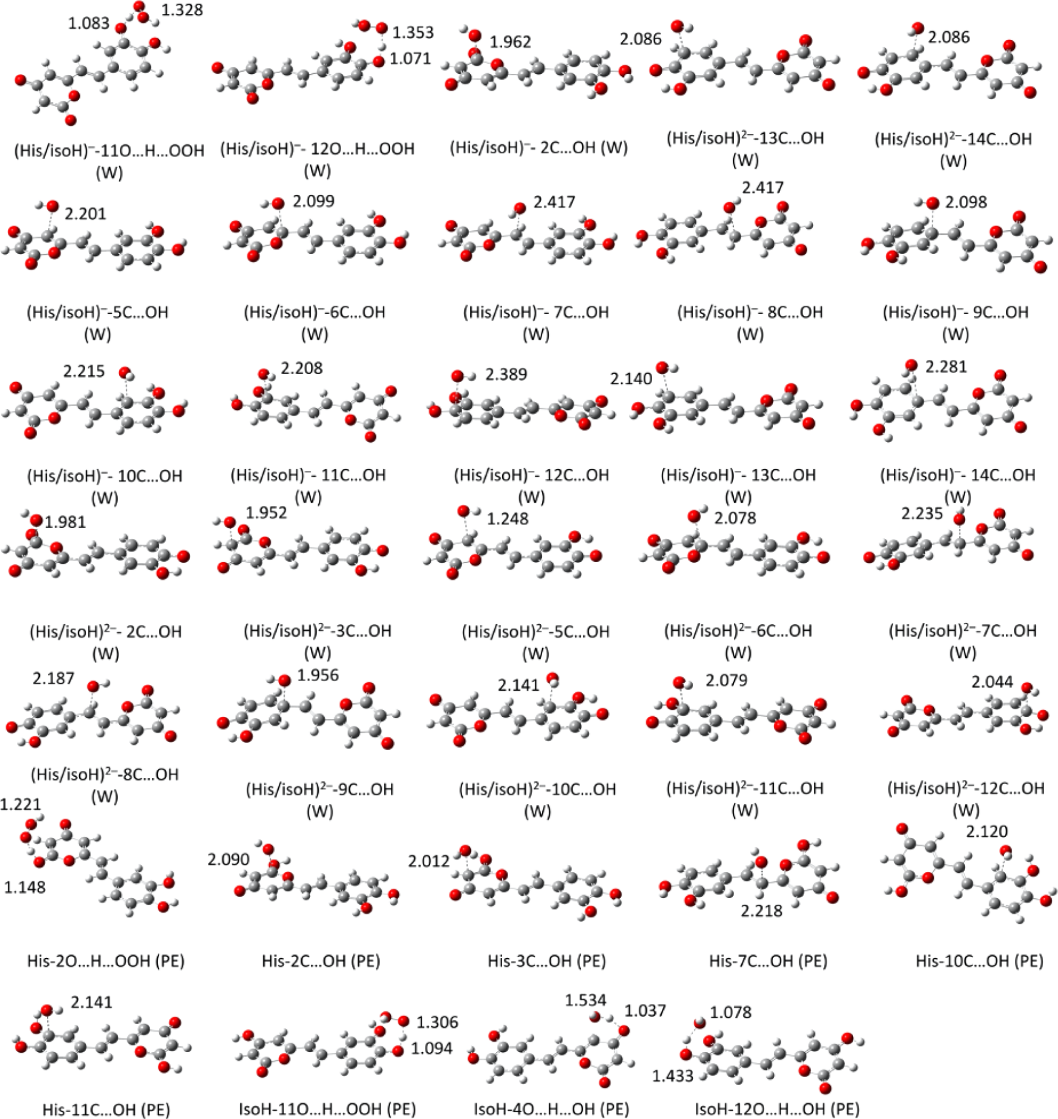
Localized transition states of the reaction of **His** and **isoH** with HO^•^ and HOO^•^ radicals in physiological media.

In accordance with the outcomes demonstrated in the gas phase study, only the most active sites were examined in lipid media, i.e., pentylpentanoate. Whereas in water, where both dissociated forms are present, all sites were examined. The obtained results are shown in Tables 4– 5,6, and the localized transition states (TSs) are represented in Figure 4.

**Table 4:** Kinetic Data of the Reactions of HO^•^ and HOO^•^ Radicals with **His** in Pentyl Ethanoate

	Mechanisms		IF^a^ (cm^–1^)	Δ*G^≠^*^b^ (kcal/mol)	κ^c^	*K*^d^(M^–1^ s^–1^)	Γ^e^ (%)	*k*_overall_ (M^–1^ s^–1^)
HOO^•^	*f*-HAT	11OH	–4076.0	14.9	239.33	1.70 × 10^4^	87	1.94 × 10^4^
		12OH	–2127.7	15.7	125.3	2.46 × 10^3^	13	
	RAF	C8	–764.6	19.0	1.89	1.39 × 10^–1^	0	
HO^•^	*f*-HAT	4OH	–545.0	5.4	1.0	5.52 × 10^8^	3	2.06 × 10^10^
		11OH^f^	–	–	–	2.80 × 10^9^	14	
		12OH	–1149.7	9.3	3.5	3.39 × 10^6^	0	
	RAF	C3	–208.3	1.6	1	2.58 × 10^9^	13	
		C4	–453.7	8.2	1.23	7.61 × 10^6^	0	
		C5	–295.5	3.2	1	2.35 × 10^9^	11	
		C6	–385.2	6.2	1.14	1.83 × 10^8^	1	
		C7	–308.7	1.9	1	2.55 × 10^9^	12	
		C8	–340.7	3.6	1	2.17 × 10^9^	11	
		C9	–393.7	8.2	1.17	6.83 × 10^6^	0	
		C10	–298.0	3.6	1	2.12 × 10^9^	10	
		C11	–228.7	3.5	1	2.18 × 10^9^	11	
		C12	–305.9	3.7	1	2.10 × 10^9^	10	
		C13	–424.3	7.8	1.2	1.31 × 10^7^	0	
		C14	–397.5	5.0	1.14	9.27 × 10^8^	5	

a Imaginary frequency.

b Activation free energy.

c Tunneling correction.

d Rate constant,.

e Branching ratio.

f The relaxed scan indicates that the reaction is barrierless and the spontaneous H-transfer takes place.

**Table 5:** Kinetic Data of the Reactions of HO^•^ and HOO^•^ Radicals with **IsoH** in Pentyl Ethanoate

	Mechanisms		IF^a^(cm^–1^)	Δ*G*^≠^^b^(kcal/mol)	κ^c^	*K*^d^(M^–1^ s^–1^)	Γ^e^(%)	*k*_overall_ (M^–1^ s^–1^)
HOO^•^	*f*-HAT	2OH	–2515.6	18.3	609.3	1.40 × 10^2^	100	1.40 × 10^2^
HO^•^	RAF	C2	–329.9	6.3	0.0	1.60 × 10^8^	4	4.48 × 10^9^
		C3	–322.3	5.9	0.1	3.60 × 10^8^	8	
		C7	–309.8	4.8	0.0	3.60 × 10^9^	80	
		C10	–329.9	6.3	0.0	1.60 × 10^8^	4	
		C11	–233.2	6.1	0.1	2.00 × 10^8^	4	

a Imaginary frequency.

b Activation free energy.

c Tunneling correction.

d Rate constant

e Branching ratio.

**Table 6:** Kinetic Data of the Reactions of HO^•^ and HOO^•^ Radicals with **His** in Water at pH = 7.4

Radical	Mechanisms		State	Δ*G^≠^*^a^ (kcal/mol)	κ^b^	*k*_app_^c^(M^–1^ s^–1^)	*f*^d^	*k*_f_^e^ (M^–1^ s^–1^)	Γ^f^ (%)	*k*_overall_ (M^–1^ s^–1^)
HOO^•^	*f*-HAT	11OH	**His**^**–**^	16.4	1450.1	9.30 × 10^3^	0.975	9.07 × 10^3^	0	1.40 × 10^8^
		12OH		16.0	778.2	9.50 × 10^3^		9.26 × 10^3^	0	
		11OH	**His**^**2–**^	-	-	3.40 × 10^9^^g^	0.025	8.50 × 10^7^	61	
	SET		**His**^**–**^	21.2	18.8^g^	1.50 × 10^–3^	0.975	1.46 × 10^–3^	0	
			**His**^**2–**^	4.2	15.6^g^	2.20 × 10^9^	0.025	5.50 × 10^7^	39	
HO^•^	RAF	C2	**His**^**–**^	∼0	1	2.11 × 10^9^	0.975	2.06 × 10^9^	6	3.24 × 10^10^
		C3		-	-	3.20 × 10^9^^g^		3.20 × 10^9^	10	
		C5		∼0	1	2.74 × 10^9^		2.67 × 10^9^	8	
		C6		3.2	1	2.10 × 10^9^		2.04 × 10^9^	6	
		C7		∼0	1	2.60 × 10^9^		2.53 × 10^9^	8	
		C8		0.8	1	2.50 × 10^9^		2.44 × 10^9^	8	
		C9		3.4	1	2.04 × 10^9^		1.99 × 10^9^	6	
		C10		1.0	1	2.38 × 10^9^		2.32 × 10^9^	7	
		C11		2.1	1	2.34 × 10^9^		2.28 × 10^9^	7	
		C12		0.6	1	2.56 × 10^9^		2.50 × 10^9^	8	
		C13		3.0	1	2.17 × 10^9^		2.11 × 10^9^	7	
		C14		0.9	1	2.45 × 10^9^		2.39 × 10^9^	7	
		C2	**His**^**2–**^	∼0	1	2.11 × 10^9^	0.025	5.27 × 10^7^	0	
		C3		∼0	1	2.08 × 10^9^		5.19 × 10^7^	0	
		C5		∼0	1	2.39 × 10^9^		5.98 × 10^7^	0	
		C6		1.8	1	2.19 × 10^9^		5.48 × 10^7^	0	
		C7		∼0	1	2.38 × 10^9^		5.95 × 10^7^	0	
		C8		∼0	1	2.33 × 10^9^		5.82 × 10^7^	0	
		C9		∼0	1	2.08 × 10^9^		5.20 × 10^7^	0	
		C10		∼0	1	2.28 × 10^9^		5.70 × 10^7^	0	
		C11		∼0	1	2.21 × 10^9^		5.53 × 10^7^	0	
		C12		∼0	1	2.18 × 10^9^		5.44 × 10^7^	0	
		C13		∼0	1	2.22 × 10^9^		5.55 × 10^7^	0	
		C14		∼0	1	2.22 × 10^9^		5.55 × 10^7^	0	
	SET		**His**^**–**^	6.8	1.3^h^	3.30 × 10^9^	0.975	3.22 × 10^9^	10	
			**His**^**2–**^	3.6	21.9^h^	5.20 × 10^–4^	0.025	1.30 × 10^–5^	0	

a Activation energy.

b Tunneling correction.

c Apparent rate constant.

d Mole fraction.

e *k*_f_ = *f*.*k*_app_.

f Branching ratio.

g Diffusion rate constant.

h The nuclear reorganization energy (λ).

The results indicate that the two tautomers exhibit different reactivities in lipid media. **IsoH** shows a lower rate constant for HOO^•^ scavenging compared to **His** (1.40 × 10^2^ vs 1.94 × 10^4^ M^–1^ s^–1^), though **His** is slightly more active than **IsoH** against the HO^•^ radical (2.06 × 10^10^ vs 4.48 × 10^9^ M^–1^ s^–1^). Mechanistically, the *f*-HAT pathway is the sole mechanism for HOO^•^ scavenging in both tautomers, with 2OH as the exclusive site for **IsoH** and 11OH for **His**. Regarding the HO^•^ radical, both tautomers primarily react via the RAF mechanism, though at different reaction sites. These findings emphasize the crucial role of tautomerization in influencing the antiradical activity of polyphenols, particularly in lipid environments. When compared to other natural antioxidants, the highest rate constant obtained for HOO^•^ in lipid media (1.94 × 10^4^ M^–1^ s^–1^) is comparable to that of feruloylquinic acid (*k*_overall_ = 4.10 × 10^4^ M^–1^ s^–1^)^41^ and BHT (*k*_overall_ = 1.70 × 10^4^ M^–1^ s^–1^)^26^ and surpasses that of Trolox (*k*_overall_ = 3.40 × 10^3^ M^–1^ s^–1^),^29^ ascorbic acid (*k*_overall_ = 5.71 × 10^3^ M^–1^ s^–1^, M052X/6-311++G(d,p)),^29^ and cannabidiolic acid (*k*_overall_ = 1.36 × 10^3^ M^–1^ s^–1^).^25^ This suggests that hispidin is an effective HOO^•^ scavenger in lipid media. Concerning the HO^•^ radical, the highest rate constant for hispidin (2.06 × 10^10^ M^–1^ s^–1^) is quite comparable to that of BHT (1.13 × 10^11^ M^–1^ s^–1^),^26^ indicating that hispidin is a potent HO^•^ scavenger.

On the other hand, the reaction of HO^•^ and HOO^•^ radicals with **His** in water at physiological pH differs from those in the gas phase and in lipid media. For the HOO^•^ radical, **His** mainly reacts via the *f*-HAT and SET mechanisms from the doubly deprotonated form (**His**^**2–**^). The overall rate constant is 1.40 × 10^8^ M^–1^ s^–1^, significantly higher than that of Trolox (*k*_overall_ = 8.96 × 10^4^ M^–1^ s^–1^),^29^ BHT (*k*_overall_ = 1.52 × 10^5^ M^–1^ s^–1^),^26^ ascorbic acid (*k*_overall_ = 9.97 × 10^7^ M^–1^ s^–1^, M052X/6-311++G(d,p)),^29^ and feruloylquinic acid (*k*_overall_ = 2.28 × 10^7^ M^–1^ s^–1^),^41^ explaining the previously obtained experimental results. It is important to note that the *f*-HAT reaction from 11OH is the most favorable site, contributing 61% to the overall rate constant. This is due to the high rate constant of this pathway, which equals the diffusion rate constant. This property has been observed in other antioxidants containing the catechol unit such as quercetins,^42^ 5-*O*-methylnorbergenin,^43^ anthocyanidins,^44^ and caftaric acid.^45^ For the SET mechanism, the **His**^**2–**^ form is the most active despite its low concentration at physiological pH, as observed for other antioxidants.^25,45^ Regarding the HO^•^ radical, the reaction appears to be nonselective and can occur at any position with comparable rate constants (*k*_app_ ∼ 10^9^ M^–1^ s^–1^). The molar fraction has been found to be the decisive factor determining the reactive species. **His**^**–**^ determines the overall reactivity of **His** with HO^•^ in water. The overall rate constant is 3.24 × 10^10^ M^–1^ s^–1^, comparable to that of rosmarinic acid (*k*_overall_ = 2.89 × 10^10^ M^–1^ s^–1^). It is important to note that SET is more favored from **His**^**–**^ than from **His**^**2**–^, which is the opposite of what was obtained with the HOO^•^ radical. This observation indicates that the nature of the radical can completely change the mechanism of an antioxidant, hence the need to examine a maximum number of free radicals when studying the antioxidant mechanism.

Overall, for the HOO^•^ radical, hispidin exhibited moderate to good activity in lipid media by reacting via the *f*-HAT mechanism. This activity becomes important in water, where hispidin showed an overall rate constant higher than those of Trolox and BHT standards. On the other hand, the reaction with the HO^•^ radical was found to be selective at the C7 position through the RAF mechanism for **IsoH** in lipid media, whereas it exhibited complete nonselectivity for **His** in lipid media and for both **IsoH** and **His** in aqueous media. By a combination of experimental and theoretical results, it can be concluded that hispidin is a potent antioxidant under physiological conditions.

### 3.3 Metal Chelating Properties

Antiradical activity is not the only beneficial property exhibited by polyphenols like hispidin. Another important feature is their antioxidative potential, which is grounded in their interactions with d-block metals. From a physiological standpoint, copper(II) and iron(III) play detrimental roles due to their involvement in Fenton-like reactions that trigger significant oxidative stress, contributing to the development of serious diseases such as Alzheimer’s and Parkinson’s.^46^

Dietary antioxidants can either amplify oxidative stress by accelerating the Fenton reaction or mitigate it by chelating metals and altering their oxidative potential or by scavenging hydroxyl radicals generated *in statu nascendi* processes, as evidenced in previous studies.^22,23,23−48^ To address this subject, adequate computational studies following the protocols from previously referenced papers were conducted. As stated, the main reaction of interest in terms of pro-oxidative behavior is the reduction of the cupric cation to the cuprous cation,^49^ depicted by the reaction scheme below:
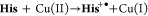
5

This yields a state that can easily generate hazardous hydroxyl radicals:
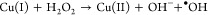
6

A square-planar, four-water-coordinated Cu(II) complex was considered as a target in accordance with literature reports.^50^ To keep the computations reasonable, the resulting Cu(I) was also modeled with four solvent molecules, though only two formed a solvation sphere, following experimental evidence.^51,52^

It is worth mentioning that reaction eq 6 may also be stimulated by biological antioxidants such as ascorbate (Asc^–^) and the superoxide anion radical (O_2_^–•^); however, such processes are physiologically controlled and rarely dangerous.^52^

To confirm the reliability of the chosen computational approach, the kinetic constants for the aforementioned reactions were established. The results, *k* = 3.72 × 10^9^ [M^–1^ s^–1^] for O_2_^•–^/O_2_ and *k* = 8.82 × 10^8^ [M^–1^ s^–1^] for Asc^–^/Asc^•^, are consistent with their corresponding experimental values.^53−55^ This substantiates the reaction rate of Cu(II) by **His**^**–**^ to be *k* = 1.16 × 10^4^ [M^–1^ s^–1^] and *k* = 3.75 × 10^9^ [M^–1^ s^–1^] for **His**^**2–**^. Therefore, the predominant monoanionic species reveals itself as a much weaker reducing agent than either of the physiological antioxidants, whereas the activity of the dianion is very similar to that of the superoxide anion radical.

The chelating properties of dietary antioxidants originate from their structure, which is rich in phenolic groups. In the case of **His**, these groups are located at positions C11 and C12, making these sites likely candidates for coordinating copper ions. Both unidentate and bidentate forms of the mono complex, as well as the bis complex, were considered primarily to assess the Maxwell–Boltzmann distribution of each. This approach consistent with previous works^47^ ensures that the study relies on the species that are actually present.

**Figure 5:**
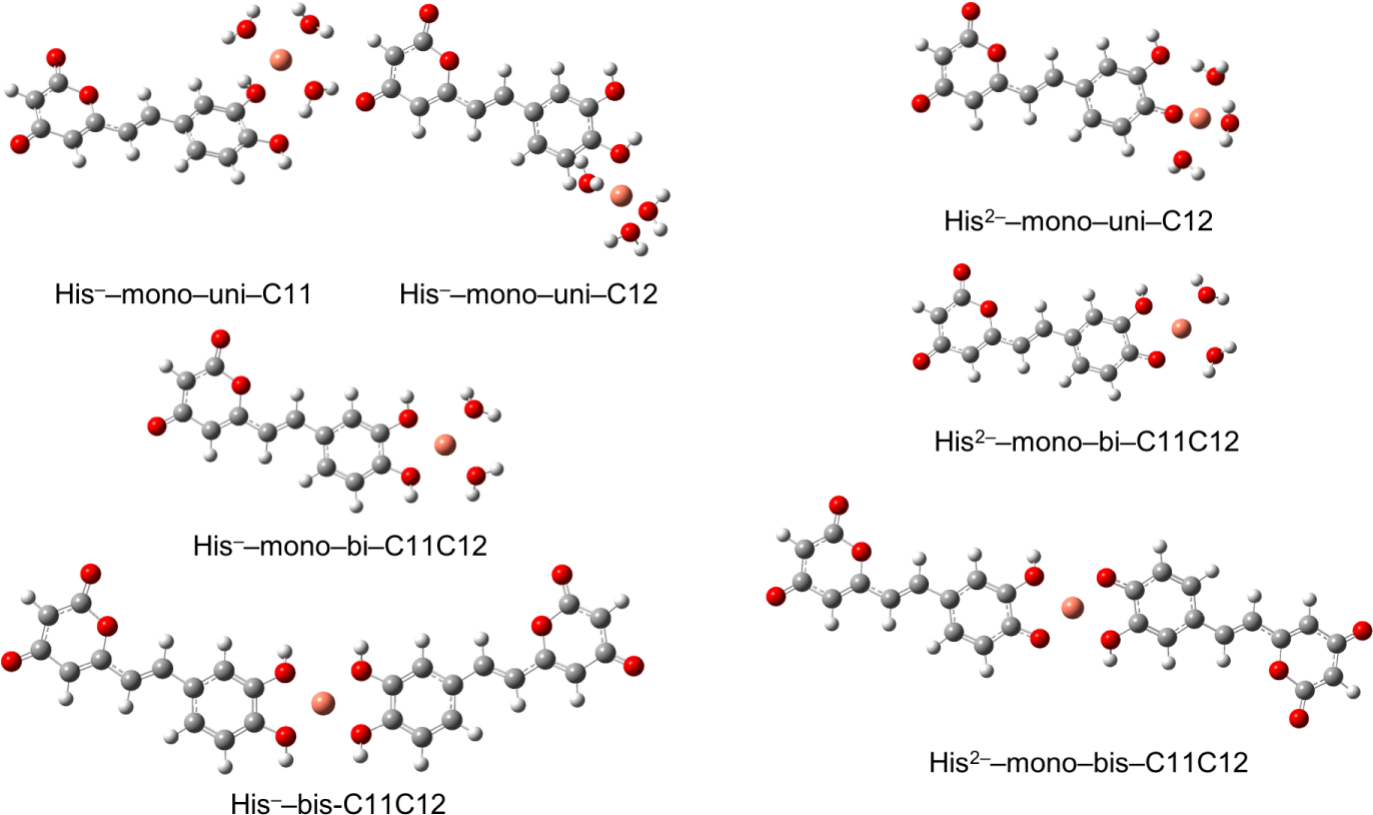
Structures of the formable Cu (II)–His complexes labeled in accordance to Table 7.

Each complex formed by **His**^**–**^ is present in a non-negligible population. In the case of **His**^**2–**^, however, only the bis structure was found (Table 7 and Figure 5). It seems that position C12 is slightly more favorable than C11 for unidentate complexes, and the bidentate complex is more desirable than the unidentate complex. Surprisingly, forming a bidentate mono complex is favored over the bis complex (−3.9 vs −1.9 kcal/mol), making the bidentate mono form the prevalent species in the instance of **His**^**–**^. For **His**^**2–**^, the bis complex is the only expected geometry, as indicated by the significant exergonicity of the chelation reaction and its associated rate constant of 1.18 × 10^33^ M^–1^ s^–1^. In all cases, copper(II) maintains its square-planar geometry without significant deviations among the generated structures

**Table 7:** Gibbs Free Energies of Complexation (in kcal mol^–1^), Equilibrium Constants, and Maxwell–Boltzmann Distribution (%) in Water at pH = 7.4

Species	Form^a^		Site	Δ*G*	*K*_i_	M-B
**His**^**–**^	mono	uni	C11	–0.8	3.86 × 10°	0.49
			C12	–1.6	1.39 × 10^1^	1.78
		bi	C11C12	–3.9	7.38 × 10^2^	94.68
	bis		C11C12	–1.9	2.37 × 10^1^	3.04
**His**^**2–**^	mono	uni	C11^b^	-	-	-
			C12	–20.6	1.35 × 10^15^	0.00
		bi	C11–C12	–27.3	9.79 × 10^19^	0.00
	bis		C11–C12	–45.1	1.18 × 10^33^	100.00

a The nomenclature follows standard coordination chemistry conventions.

b The complex does not form. Instead, it optimizes into the C4” form.

The chemical behavior of **His–**Cu complex toward physiological reductants was investigated, and the results are displayed in Table 8. The findings suggest that complexes formed with **His**^**–**^ only slightly decrease the reaction rate with the reaction mixture with O_2_^•–^. More favorable properties are observed for **His**^**2–**^, where the rate constant decreases by over 10-fold. Interestingly, reduction by ascorbate appears more efficient when copper is complexed with **His**^**–**^ rather than in its free aquated form. Simultaneously, the reactivity of the **His**^**2–**^–Cu(II) chelate is noticeably reduced. Based on these outcomes, the dianionic form demonstrates the highest efficacy in inhibiting direct reduction processes.

**Table 8:** Energies of Reactions (Δ*E*, in kcal mol^–1^), Gibbs Free Energies of Reaction (Δ*G*, in kcal mol^–1^), Reorganization Energies (λ, in kcal mol^–1^), Activation Energies (Δ*G*^≠^, in kcal mol^–1^), and Rate Constants (*k*, M^–1^ s^–1^), for the Reactions of the Complexes with the O_2_^•–^ and Asc^–^ in Water at pH = 7.4

				O_2_^•–^					Asc^–^				
Species	Form^a^	Site	Δ*E*		Δ*G*	λ	Δ*G*^≠^	*k*	Δ*E*	Δ*G*	λ	Δ*G*^≠^	*k*
Cu^2+^(H_2_O)_4_				8.9	–21.1	39.7	2.2	3.72 × 10^9^	18.0	–6.3	31.7	5.1	8.28 × 10^8^
**His**^**–**^	mono	uni	C11	6.4	–25.4	41.0	1.5	1.40 × 10^9^	15.5	26.1	32.3	3.6	1.52 × 10^9^
			C12	6.6	–25.5	41.3	1.5	1.39 × 10^9^	15.7	–10.7	32.6	3.7	1.50 × 10^9^
		bi	C11C12	2.7	–31.2	43.2	0.8	1.42 × 10^9^	11.8	–16.5	34.6	2.4	1.73 × 10^9^
	bis		C11C12	–3.2	–28.6	34.5	0.3	1.33 × 10^9^	5.9	–13.8	25.6	1.4	1.60 × 10^9^
**His**^**2–**^	bis		C11C12	21.3	–10.0	40.3	5.7	3.04 × 10^8^	30.4	4.8	31.4	10.4	1.39 × 10^5^

a The nomenclature follows standard coordination chemistry conventions.

Despite the implausible results for **His**^**–**^, this species may still be an important protector against OH-initiated damage, scavenging the radicals formed at the metal site by the polyphenolic part of the entire system. The viability of possible *f*-HAT, RAF, and SET mechanisms was assessed solely from a thermochemical standpoint. Kinetic evaluations were limited due to two circumstances: first, the hydroxyl radical is so reactive that it reacts at the diffusion-limited reaction rate with any molecule in its proximity, and second, reports suggest^56,57^ that the Bell–Evans–Polanyi principle applies to this species. Therefore, if the reaction is exergonic, then it shall proceed with a reaction rate proportional to the corresponding Gibbs free energies.

**Figure 6:**
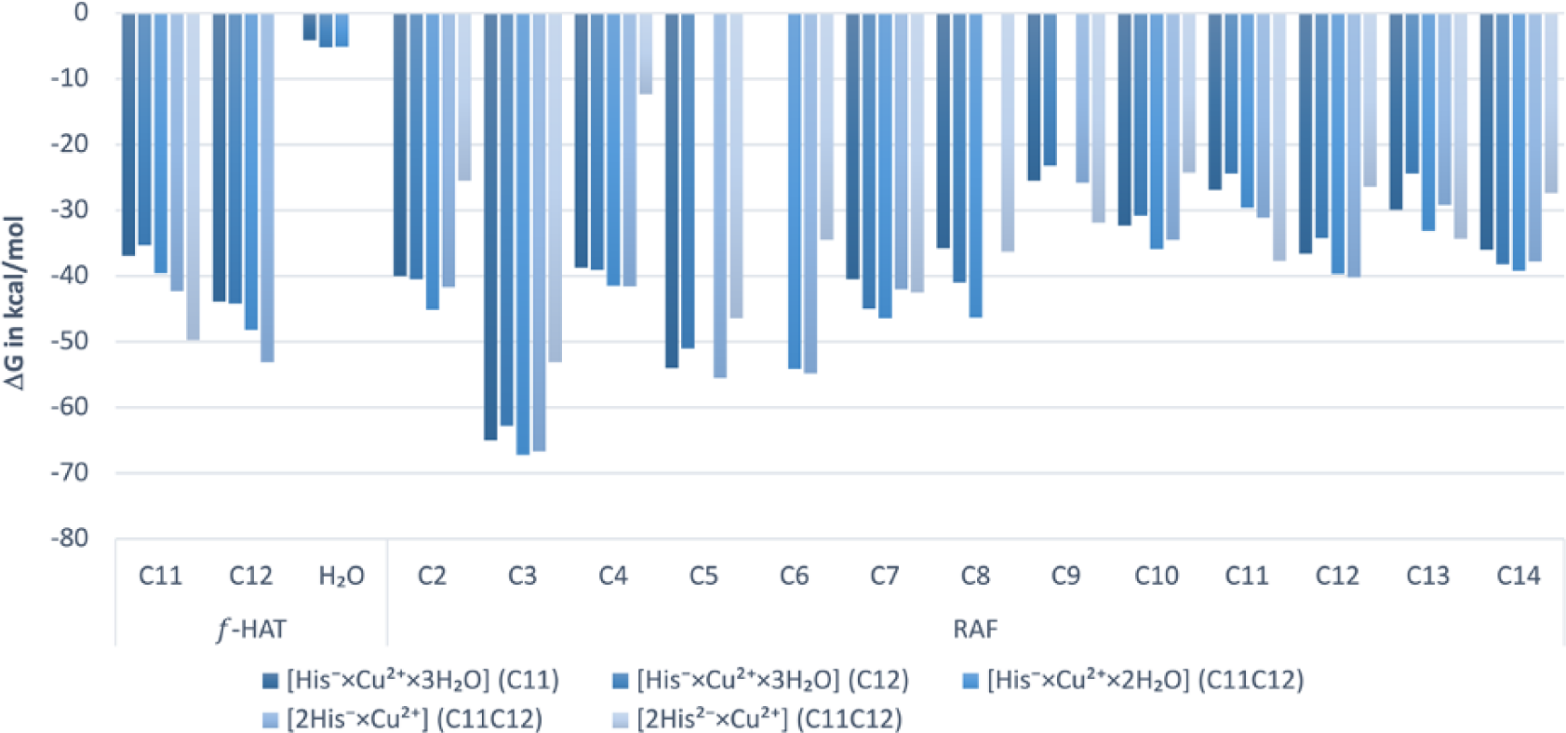
Gibbs free energies of reaction (Δ*G*, in kcal mol^–1^) for *f*-HAT and RAF pathways of the complexes with ^•^OH under the studied conditions.

Building on that, the formation of radical adducts at position C3, between the ketone and deprotonated hydroxyl, is the most readily observed product. Generally, the propensity of the RAF mechanism favors that ring, proceeding through the linker, with the catechol ring being the least prone, at least from a relative standpoint, as these processes remain highly exergonic (Figure 6). Similarly favorable characteristics are exhibited by *f*-HAT, which shows similarity to RAF regardless of the site. There is a slight preference for C12 over C11 due to the spin density delocalization of the radical formed, although the differences are marginal. Additionally, hydrogen abstraction may occur at the water-coordinating copper ion, although this pathway does not distinguish itself significantly from other possibilities. The viability of the *f*-HAT process improves when moving from unidentate to bidentate complexes, then to bis- forms, and finally considering the dianionic form of the polyphenol. No clear trends regarding the RAF propensity can be distinguished, however.

The electron transfer reactions were studied in more detail due to their entirely different character. The organized results (see Table 9) indicate that significant SET occurs only for the bis complex of **His**^**2–**^ (*k* = 9.58 × 10^8^ M^–1^ s^–1^). For the remaining complexes, the reaction rates were found to be minimal.

**Table 9:** Energies of Reactions (Δ*E*, in kcal mol^–1^), Gibbs Free Energies of Reaction (Δ*G*, in kcal mol^–1^), Reorganization Energies (λ, in kcal mol^–1^), Activation Energies (Δ*G*^≠^, in kcal mol^–1^), and Rate Constants (*k*, M^–1^ s^–1^), for the SET Pathways of the Complexes with ^•^OH under Studied Conditions

Species	Form		Site	Δ*E*	Δ*G*	λ	Δ*G*^≠^	*K*
**His**^**–**^	mono	uni	C11	30.9	16.6	24.4	17.2	1.42 × 10°
			C12	29.3	16.8	22.5	17.2	1.53 × 10°
		bi	C11C12	34.1	11.4	32.7	14.9	7.27 × 10^1^
	Bis		C11C12	39.5	14.9	34.7	17.7	6.59 × 10^–1^
**His**^**2–**^	Bis		C11C12	13.6	–4.0	27.6	5.0	9.58 × 10^8^

## 4 Conclusion

The antioxidant activity of hispidin was effectively assessed using thermodynamic and kinetic DFT calculations as well as DPPH and ABTS assays. The study demonstrated that hispidin is a potent radical scavenger under both polar and lipid physiological conditions. In polar media, both tautomers of hispidin exhibited similar HO^•^ and HOO^•^ radical scavenging capacities, with rate constants of *k* = 3.24 × 10^10^ M^–1^ s^–1^ and *k* = 1.40 × 10^8^ M^–1^ s^–1^, respectively. In contrast, in lipid media, the tautomers showed different outcomes with **His** being more reactive than **IsoH** toward the HO^•^ radical, while the reverse was observed for HOO^•^. This highlights the significant role that tautomerization plays in the antiradical activity of polyphenols. Mechanistically, the RAF mechanism was found to be decisive for HO^•^ scavenging under physiological conditions for both tautomers. Conversely, the *f*-HAT mechanism is dominant for the HOO^•^ radical in lipid media, while both *f*-HAT and SET mechanisms are viable in water at physiological pH. It is also noteworthy that, for HOO^•^ radicals in lipid media, the *f*-HAT pathway occurs exclusively at 2OH for **IsoH** and predominantly at 11OH for **His**. The study of the chelation capacity revealed that hispidin is an excellent chelator of Cu(II) ions, and its complexation prevents the generation of HO^•^ radicals via the ascorbate pathway. The theoretical calculations are consistent with the results of the DPPH and ABTS assays, highlighting the high antioxidant capacity of hispidin in physiological environments.

## Data Availability

The data underlying this study are available in the published article and its Supporting Information.

## Supporting Information Available

A schematic representation of the antioxidant mechanisms of polyphenols, details of the computational protocol (PDF)
