# Supplementary material for: A Comprehensive Experimental and Theoretical Investigation of the Antioxidant Properties of Hispidin and Isohispidin
Source: J Org Chem. 2025 Feb 25;90(9):3257–68. doi: 10.1021/acs.joc.4c02837 (PMC11894650; doi:10.1021/acs.joc.4c02837)
Supplement: Supplementary file 1 — jo4c02837_si_001.pdf [file jo4c02837_si_001.pdf]

A Comprehensive Experimental and Theoretical Investigation of The Antioxidant Properties of Hispidin and Isohispidin

Houssem BOULEBD[1], Imene AMINE KHODJA[1], Khedidja BENAROUS[2],

Marcin MĄCZYŃSKI[3], Maciej SPIEGEL[3,*]

[1] Laboratory of Synthesis of Molecules with Biological Interest, University of Frères Mentouri Constantine 1, Constantine 25017, Algeria.
[2]Fundamental Sciences Laboratory, Amar Telidji University, Laghouat 03000, Algeria
[3]Department of Organic Chemistry and Pharmaceutical Technology, Faculty of Pharmacy, Wroclaw Medical University, Borowska 211A, 50-556 Wroclaw, Poland
*Corresponding authors: maciej.spiegel@umw.edu.pl

**Table of Contents**	Page
**Figure S1.** Antiradical mechanisms of phenolic compounds. f-HAT: formal hydrogen transfer; RAF: radical adduct formation; SETPT: sequential electron transfer proton transfer; and SPLET: sequential proton lose electron transfer.	S2
**Table S1.** The method to calculate rate constant following the conventional transition state theory.	S3-S4
**References**	S4-S5
**XYZ-coordinates**	S6-S58

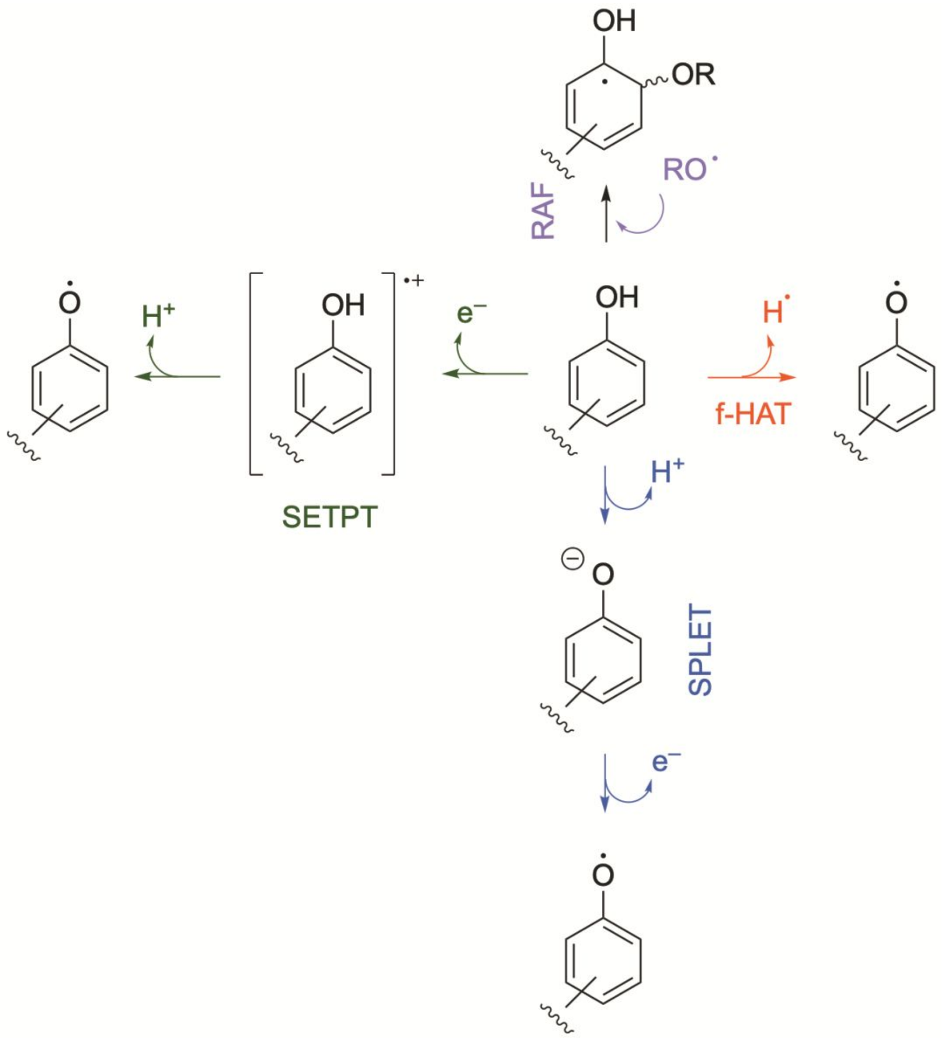

Figure S1. Antiradical mechanisms of phenolic compounds. f-HAT: formal hydrogen transfer; RAF: radical adduct formation; SETPT: sequential electron transfer proton transfer; and SPLET: sequential proton lose electron transfer.

Table S1. The method to calculate rate constant following the conventional transition state theory.

PA (proton affinity) values were calculated as follows.

$$\mathrm{PA} = \mathrm{H(ArO^{-})} + \mathrm{H(H^{+})} - \mathrm{H(ArOH)}$$

Where $\mathrm{H(ArOH)}$, $\mathrm{H(ArO^{-})}$, and $\mathrm{H(H^{+})}$ are enthalpies of the neutral molecule, anion molecule, and proton respectively.

The pKa values of **His** were computed according to the literature using the following equation [1]:

$$\mathrm{AH} + \mathrm{OH^{-}(3H_2O)} \rightarrow \mathrm{A^{-}(H_2O)} + 3\mathrm{H_2O}$$

$$\mathrm{pK_a} = \frac{\Delta \mathrm{G_{sol}}}{\mathrm{RTln(10)}} + 14 + 3\log{[\mathrm{H_2O}]}$$

Where DG_{sol} is the Gibbs free energy of the reaction in solution, R is the gas constant, and T is the temperature (298.15 K).

The kinetic calculations were carried out following the methodology of the quantum mechanics-based test for overall free radical scavenging activity (QM-ORSA)[2, 3]. The rate constant (*k*) was calculated by using standard transition state theory (TST) and 1M standard state at 298.15 K according to the equation below: [4-10]

$$k = \sigma\kappa \frac{k_B T}{h} e^{-(\Delta G^{\neq})/RT}$$

Where σ is the reaction symmetry number, [11, 12] κ represents tunneling corrections computed using the Eckart barrier,[13] k_B is the Boltzmann constant, *h* is the Planck constant, $\Delta G^{\neq}$ is Gibbs free energy of activation.

The apparent rate constants (k_{app}, rate constants close to the diffusion limit) were corrected using the Collins−Kimball theory [14]. Branching ratios (Γ, %) were calculated as follows:

$$\boldsymbol{?_{\mathrm{path}}} = \frac{\mathbf{k}}{\mathbf{k_{overall}}} \times \mathbf{100}$$

Where *k* and $k_{overall}$ are the rate constants of a specific reaction path and the sum of the rate constants of all reaction paths, respectively.

Marcus theory has been used to predict the Gibbs free energy of activation of the single electron transfer mechanism [15]:

$$\Delta G^{\neq}_{SET} = \frac{\lambda}{4}(1 + \frac{\Delta G^{0}_{SET}}{\lambda})^{2}$$

$$\lambda \approx \Delta E_{SET} + \Delta G^{0}_{SET}$$

Where λ is the nuclear reorganization energy, ΔG^{0}_{SET} is the Gibbs free energy of reaction, and ΔE_{SET} is the nonadiabatic energy difference between reactants and vertical products. The computational kinetic study was performed with Eyringpy software[9].

References

[1] Rebollar-Zepeda, A. M.; Campos-Hernández, T.; Ramírez-Silva, M. T.; Rojas-Hernández, A.; Galano, A. Searching for Computational Strategies to Accurately Predict p Ka s of Large Phenolic Derivatives. J. Chem. Theory Comput. 2011, 7 (8), 2528–2538. https://doi.org/10.1021/ct2001864.

[2] Galano, A.; Alvarez ‐ Idaboy, J. R. A Computational Methodology for Accurate Predictions of Rate Constants in Solution: Application to the Assessment of Primary Antioxidant Activity. J Comput Chem 2013, 34 (28), 2430 – 2445. https://doi.org/10.1002/jcc.23409.

[3] Galano, A.; Alvarez ‐ Idaboy, J. R. Computational Strategies for Predicting Free Radical Scavengers' Protection against Oxidative Stress: Where Are We and What Might Follow? Int J of Quantum Chemistry 2019, 119 (2), e25665. https://doi.org/10.1002/qua.25665.

[4] Evans, M. G.; Polanyi, M. Some Applications of the Transition State Method to the Calculation of Reaction Velocities, Especially in Solution. Trans. Faraday Soc. 1935, 31, 875. https://doi.org/10.1039/tf9353100875.

[5] Eyring, H. The Activated Complex in Chemical Reactions. The Journal of Chemical Physics 1935, 3 (2), 107–115. https://doi.org/10.1063/1.1749604.

[6] Truhlar, D. G.; Garrett, B. C.; Klippenstein, S. J. Current Status of Transition-State Theory. J. Phys. Chem. 1996, 100 (31), 12771–12800. https://doi.org/10.1021/jp953748q.

[7] Furuncuoğlu, T.; Uğur, İ.; Değirmenci, İ.; Aviyente, V. Role of Chain Transfer Agents in Free Radical Polymerization Kinetics. Macromolecules 2010, 43 (4), 1823–1835. https://doi.org/10.1021/ma902803p.

[8] Vélez, E.; Quijano, J.; Notario, R.; Pabón, E.; Murillo, J.; Leal, J.; Zapata, E.; Alarcón, G. A Computational Study of Stereospecifity in the Thermal Elimination Reaction of Menthyl Benzoate in the Gas Phase. J of Physical Organic Chem 2009, 22 (10), 971–977.

https://doi.org/10.1002/poc.1547.
[9] Dzib, E.; Cabellos, J. L.; Ortíz ‐ Chi, F.; Pan, S.; Galano, A.; Merino, G. Eyringpy : A Program for Computing Rate Constants in the Gas Phase and in Solution. Int J of Quantum Chemistry 2019, 119 (2), e25686. https://doi.org/10.1002/qua.25686.
[10] Boulebd, H.; Khodja, I. A.; Bay, M. V.; Hoa, N. T.; Mechler, A.; Vo, Q. V. Thermodynamic and Kinetic Studies of the Radical Scavenging Behavior of Hydralazine and Dihydralazine: Theoretical Insights. J. Phys. Chem. B 2020, 124 (20), 4123–4131. https://doi.org/10.1021/acs.jpcb.0c02439.
[11] Pollak, E.; Pechukas, P. Symmetry Numbers, Not Statistical Factors, Should Be Used in Absolute Rate Theory and in Broensted Relations. J. Am. Chem. Soc. 1978, 100 (10), 2984–2991. https://doi.org/10.1021/ja00478a009.
[12] Fernández-Ramos, A.; Ellingson, B. A.; Meana-Pañeda, R.; Marques, J. M. C.; Truhlar, D. G. Symmetry Numbers and Chemical Reaction Rates. Theor Chem Account 2007, 118 (4), 813–826. https://doi.org/10.1007/s00214-007-0328-0.
[13] Eckart, C. The Penetration of a Potential Barrier by Electrons. Phys. Rev. 1930, 35 (11), 1303–1309. https://doi.org/10.1103/PhysRev.35.1303..
[14] Collins, F. C.; Kimball, G. E. Diffusion-Controlled Reaction Rates. Journal of Colloid Science 1949, 4 (4), 425–437. https://doi.org/10.1016/0095-8522(49)90023-9.
[15] Corchado, J. C.; Coitiño, E. L.; Chuang, Y.-Y.; Fast, P. L.; Truhlar, D. G. Interpolated Variational Transition-State Theory by Mapping. J. Phys. Chem. A 1998, 102 (14), 2424–2438. https://doi.org/10.1021/jp9801267.

==> AOX-I/PE/HIS_10C_HO <==
30
DATASET

C	-1.575675	0.636263	-0.089148
C	-2.333899	1.756016	0.237609
C	-3.729673	1.711836	0.252304
C	-4.396778	0.533964	-0.051052
C	-3.654004	-0.600801	-0.391281
C	-2.253704	-0.567385	-0.419368
O	-4.352599	-1.730438	-0.653456
O	-5.747118	0.483978	-0.012411
C	-0.120958	0.715526	-0.071971
C	0.707438	-0.341436	-0.093532
C	2.153421	-0.221847	-0.068667
C	2.993395	-1.284251	-0.052109
C	4.398250	-1.032952	-0.026074
C	4.878118	0.242411	-0.014419
C	3.974629	1.356645	-0.029858
O	2.618420	1.044119	-0.059502
O	5.178867	-2.121880	-0.012661
H	-1.830254	2.682250	0.490007
H	-4.310556	2.586541	0.520208
H	-1.730907	-1.369623	-0.924070
H	0.300092	1.714705	-0.008147
H	0.329400	-1.357995	-0.104233
H	-3.826396	-2.485124	-0.350707
H	-6.029190	-0.422221	-0.196708
H	5.936939	0.465224	0.005161
O	4.259266	2.527346	-0.020971
H	2.610523	-2.294735	-0.057202
H	6.110949	-1.866963	0.008928
O	-2.138367	-1.925597	1.209599
H	-2.351711	-1.296215	1.917364

ENERGIES
SCF ENERGY: -953.182128837
SUM OF ELECTRONIC AND ZERO-POINT ENERGIES: -952.961610
SUM OF ELECTRONIC AND THERMAL ENERGIES: -952.944194
SUM OF ELECTRONIC AND THERMAL ENTHALPIES: -952.943249
SUM OF ELECTRONIC AND THERMAL FREE ENERGIES: -953.008400

==> AOX-I/PE/HIS_11C_HO <==
30
DATASET

C	-1.463854	0.509637	0.134097
C	-2.202459	1.702733	-0.033592
C	-3.583464	1.723010	0.007551
C	-4.276494	0.533223	0.215985
C	-3.559215	-0.686311	0.338498
C	-2.158815	-0.672467	0.358834
O	-4.287809	-1.778525	0.724145
O	-5.607055	0.528097	0.290141
C	-0.003010	0.584540	0.085598
C	0.844787	-0.454695	0.110283
C	2.288346	-0.304323	0.062290
C	3.153044	-1.346726	0.076039
C	4.551249	-1.064162	0.020639
C	5.000969	0.220537	-0.044380
C	4.071800	1.313211	-0.059887
O	2.723440	0.970195	-0.002042
O	5.356639	-2.134957	0.035969
H	-1.662778	2.629775	-0.194787
H	-4.139850	2.644996	-0.106351
H	-1.649078	-1.613720	0.531084
H	0.409472	1.586940	0.015945
H	0.496976	-1.480468	0.164151
H	-4.249691	-2.416284	-0.003476
H	-5.898298	-0.385517	0.433338
H	6.053722	0.467178	-0.086286
O	4.327694	2.489024	-0.117403
H	2.795424	-2.365066	0.127686
H	6.282349	-1.860041	-0.005659
O	-3.915369	-1.176287	-1.719333
H	-3.195871	-0.737400	-2.199186

ENERGIES
SCF ENERGY: -953.181502956
SUM OF ELECTRONIC AND ZERO-POINT ENERGIES: -952.961383
SUM OF ELECTRONIC AND THERMAL ENERGIES: -952.943829
SUM OF ELECTRONIC AND THERMAL ENTHALPIES: -952.942885
SUM OF ELECTRONIC AND THERMAL FREE ENERGIES: -953.008547

==> AOX-I/PE/HIS_11OH_OOH <==
31
DATASET

C	1.125466	-1.086474	-0.049135
C	1.696237	-2.360557	0.181325
C	3.063629	-2.569210	0.159759
C	3.905857	-1.492921	-0.092154
C	3.358124	-0.201622	-0.347176
C	1.971279	-0.016763	-0.320538
O	4.221532	0.759449	-0.615825
O	5.231366	-1.648841	-0.109132
C	-0.331011	-0.967099	-0.000378
C	-1.034128	0.167896	-0.137557
C	-2.483314	0.220229	-0.080095
C	-3.196147	1.365365	-0.203051
C	-4.619536	1.285252	-0.127216
C	-5.241161	0.087520	0.062399
C	-4.470768	-1.116182	0.190680
O	-3.088581	-0.969613	0.108814
O	-5.269284	2.449995	-0.254176
H	1.036473	-3.197090	0.383466
H	3.489362	-3.547838	0.343957
H	1.590315	0.977048	-0.526106
H	-0.872052	-1.894083	0.167114
H	-0.553504	1.126600	-0.297506
H	5.630410	-0.783955	-0.296622
H	-6.317875	-0.006105	0.122940
O	-4.885818	-2.234208	0.360492
H	-2.701789	2.314599	-0.352965
H	-6.223809	2.311513	-0.191103
H	4.008252	1.651989	0.013402
O	3.860373	2.558605	0.870782
O	4.131067	3.713152	0.200956
H	4.841811	4.126389	0.716954

ENERGIES
SCF ENERGY: -1028.32756134
SUM OF ELECTRONIC AND ZERO-POINT ENERGIES: -1028.106998
SUM OF ELECTRONIC AND THERMAL ENERGIES: -1028.088244
SUM OF ELECTRONIC AND THERMAL ENTHALPIES: -1028.087299
SUM OF ELECTRONIC AND THERMAL FREE ENERGIES: -1028.158708

==> AOX-I/PE/HIS_12C_HO <==
30
DATASET

C	1.442802	0.403759	-0.187088
C	2.197806	1.592272	-0.108742
C	3.574585	1.569021	-0.145271
C	4.254410	0.340201	-0.218125
C	3.492594	-0.855099	-0.358481
C	2.113122	-0.820702	-0.323357
O	4.230809	-1.969460	-0.533535
O	5.575629	0.331542	-0.443293
C	-0.010608	0.510049	-0.132347
C	-0.881089	-0.511625	-0.066049
C	-2.319862	-0.326364	-0.024278
C	-3.206091	-1.347612	0.059020

C -4.598249 -1.032574 0.093935
C -5.020664 0.261828 0.045350
C -4.068796 1.332133 -0.042126
O -2.728033 0.957766 -0.073542
O -5.424344 -2.084229 0.176352
H 1.677128 2.539214 -0.024872
H 4.162985 2.476909 -0.093667
H 1.565474 -1.751243 -0.427288
H -0.403106 1.522638 -0.139956
H -0.556453 -1.545668 -0.032214
H 3.681407 -2.760571 -0.474701
H 5.911099 -0.563171 -0.293590
H -6.067748 0.534289 0.069460
O -4.301189 2.512834 -0.091503
H -2.869713 -2.373700 0.099232
H -6.344438 -1.788628 0.196010
O 4.381149 -0.148440 1.883134
H 3.551420 0.218084 2.226023

ENERGIES
SCF ENERGY: -953.179861551
SUM OF ELECTRONIC AND ZERO-POINT ENERGIES: -952.960219
SUM OF ELECTRONIC AND THERMAL ENERGIES: -952.942363
SUM OF ELECTRONIC AND THERMAL ENTHALPIES: -952.941419
SUM OF ELECTRONIC AND THERMAL FREE ENERGIES: -953.008257

==> AOX-I/PE/HIS_12OH_HO <==
30
DATASET
C 1.403371 0.528292 -0.201699
C 2.146470 1.718620 0.024102
C 3.518993 1.711472 0.041581
C 4.220404 0.513727 -0.181400
C 3.474533 -0.670197 -0.511619
C 2.080980 -0.651848 -0.486023
O 4.187957 -1.712622 -0.939202
O 5.536952 0.447284 -0.102015
C -0.052405 0.613680 -0.141994
C -0.889327 -0.434942 -0.084330
C -2.333234 -0.302055 -0.021757
C -3.176940 -1.358017 0.051860
C -4.580847 -1.097438 0.108580
C -5.050697 0.180540 0.095891
C -4.141063 1.289031 0.018537
O -2.787553 0.966837 -0.040761
O -5.364348 -2.181342 0.175399
H 1.606258 2.639080 0.215307
H 4.091363 2.606112 0.254256
H 1.542877 -1.560636 -0.735822
H -0.467387 1.616816 -0.121021
H -0.525049 -1.456139 -0.062635
H 3.628454 -2.496127 -1.016069
H -6.106909 0.412087 0.140630
O -4.419488 2.459957 -0.001560
H -2.800477 -2.370857 0.066993
H -6.295064 -1.922995 0.215116
H 5.603007 -0.323378 0.589107
O 4.760008 -1.079616 1.624005
H 4.693263 -0.593139 2.456830

ENERGIES
SCF ENERGY: -953.170019541
SUM OF ELECTRONIC AND ZERO-POINT ENERGIES: -952.952448
SUM OF ELECTRONIC AND THERMAL ENERGIES: -952.935192
SUM OF ELECTRONIC AND THERMAL ENTHALPIES: -952.934248
SUM OF ELECTRONIC AND THERMAL FREE ENERGIES: -952.999275

==> AOX-I/PE/HIS_12OH_HOO <==
31
DATASET
C 1.051409 0.255099 -0.421631
C 1.826134 1.424352 -0.635120
C 3.198119 1.351472 -0.768664
C 3.837833 0.103913 -0.683166
C 3.066532 -1.065915 -0.472471
C 1.692564 -0.990965 -0.342828
O 3.693974 -2.275048 -0.398248
O 5.171513 -0.055123 -0.836557
C -0.394310 0.409824 -0.293996
C -1.286436 -0.585694 -0.132861
C -2.710541 -0.358614 -0.010585
C -3.643322 -1.328712 0.150810
C -5.015801 -0.946914 0.255272
C -5.401805 0.359525 0.193465
C -4.427953 1.394285 0.025971
O -3.086827 0.964715 -0.070519
O -5.886335 -1.985043 0.417937
H 1.324037 2.379696 -0.701162
H 3.799288 2.232839 -0.946592
H 1.143927 -1.906063 -0.174715
H -0.761650 1.428300 -0.340123
H -0.987992 -1.625373 -0.087098
H -6.434846 0.663749 0.267597
O -4.627947 2.605818 -0.042016
H -3.365014 -2.368934 0.199392
H -6.819833 -1.713196 0.487153
H 4.657232 -2.182732 -0.537629
O 5.933053 0.570500 1.359589
O 4.645329 0.358445 1.899573
H 4.225608 1.245178 1.967378
H 5.722628 0.242488 0.042549

ENERGIES
SCF ENERGY: -1028.02327150
SUM OF ELECTRONIC AND ZERO-POINT ENERGIES: -1027.802731
SUM OF ELECTRONIC AND THERMAL ENERGIES: -1027.784497
SUM OF ELECTRONIC AND THERMAL ENTHALPIES: -1027.783553
SUM OF ELECTRONIC AND THERMAL FREE ENERGIES: -1027.851834

==> AOX-I/PE/HIS_13C_HO <==
30
DATASET
C 1.469515 0.228900 -0.201513
C 2.253624 1.351302 -0.381219
C 3.666623 1.267752 -0.367958
C 4.261772 -0.004935 -0.321438
C 3.466055 -1.145541 -0.121818
C 2.098308 -1.036310 -0.059840
O 4.167663 -2.309429 -0.017698
O 5.597450 -0.107366 -0.402898
C 0.015237 0.385927 -0.175268
C -0.890613 -0.592058 -0.025224
C -2.323006 -0.352609 -0.003940
C -3.245745 -1.332662 0.148216
C -4.624926 -0.965288 0.158096
C -5.001046 0.337062 0.018658
C -4.011382 1.362438 -0.145116
O -2.685083 0.937949 -0.147442
O -5.489458 -1.977745 0.312080
H 1.789269 2.323997 -0.491302
H 4.265759 2.101005 -0.711370
H 1.509749 -1.934799 0.091805
H -0.340268 1.405858 -0.288694
H -0.606473 -1.631711 0.094227
H 3.575750 -3.059019 0.115109
H 5.854098 -1.033664 -0.288026
H -6.037704 0.648028 0.024470
O -4.200101 2.544407 -0.281091
H -2.947506 -2.364962 0.262916

H -6.397571 -1.646707 0.310781
O 4.049679 1.734966 1.565324
H 5.007215 1.585854 1.524069

ENERGIES
SCF ENERGY: -953.174816696
SUM OF ELECTRONIC AND ZERO-POINT ENERGIES: -952.954487
SUM OF ELECTRONIC AND THERMAL ENERGIES: -952.936867
SUM OF ELECTRONIC AND THERMAL ENTHALPIES: -952.935923
SUM OF ELECTRONIC AND THERMAL FREE ENERGIES: -953.001566

==> AOX-I/PE/HIS_14C_HO <==
30
DATASET
C 1.595368 0.202945 -0.174575
C 2.382022 1.366422 -0.375295
C 3.784146 1.267062 -0.489888
C 4.406458 0.059687 -0.287417
C 3.623906 -1.088936 -0.016370
C 2.245293 -1.019133 0.035960
O 4.341149 -2.222705 0.178318
O 5.752397 -0.042904 -0.352811
C 0.147703 0.355272 -0.154920
C -0.752830 -0.635749 -0.040920
C -2.186418 -0.411426 -0.038789
C -3.099710 -1.404993 0.082757
C -4.483667 -1.055092 0.071721
C -4.872241 0.244037 -0.059424
C -3.892081 1.283974 -0.191925
O -2.560843 0.876738 -0.170948
O -5.337911 -2.080157 0.197073
H 1.892936 2.275794 -0.696638
H 4.382425 2.145098 -0.699685
H 1.685248 -1.925957 0.238518
H -0.212270 1.375379 -0.246570
H -0.459682 -1.675736 0.052493
H 3.762325 -2.977764 0.340206
H 6.008761 -0.962639 -0.205527
H -5.912402 0.542822 -0.071166
O -4.094588 2.464150 -0.319759
H -2.790467 -2.435168 0.187407
H -6.250098 -1.760825 0.181886
O 2.130116 2.125711 1.561587
H 2.662957 1.447901 2.005900

ENERGIES
SCF ENERGY: -953.177765535
SUM OF ELECTRONIC AND ZERO-POINT ENERGIES: -952.957778
SUM OF ELECTRONIC AND THERMAL ENERGIES: -952.939962
SUM OF ELECTRONIC AND THERMAL ENTHALPIES: -952.939018
SUM OF ELECTRONIC AND THERMAL FREE ENERGIES: -953.006087

==> AOX-I/PE/HIS_3C_HO <==
30
DATASET
C 2.054818 -0.392948 0.065636
C 2.791745 -1.580852 0.039269
C 4.179626 -1.565230 -0.035246
C 4.852796 -0.355224 -0.083857
C 4.122811 0.845584 -0.056636
C 2.746199 0.830621 0.016913
O 4.886036 1.975088 -0.107400
O 6.201422 -0.328926 -0.155974
C 0.600744 -0.478836 0.141925
C -0.264879 0.550402 0.161549
C -1.698320 0.373639 0.235094
C -2.582892 1.410081 0.244170
C -3.964060 1.116568 0.288977
C -4.401634 -0.193718 0.296409
C -3.446409 -1.269341 0.374979
O -2.110507 -0.906424 0.298040
O -4.797447 2.154712 0.278764
H 2.268294 -2.529434 0.076450
H 4.752654 -2.484368 -0.056742
H 2.212496 1.775321 0.036664
H 0.200074 -1.487735 0.184252
H 0.064043 1.582258 0.116776
H 4.332677 2.764541 -0.100236
H 6.496140 0.590940 -0.186060
H -5.443850 -0.448851 0.434080
O -3.690140 -2.442816 0.480842
H -2.233751 2.431442 0.195926
H -5.714579 1.851594 0.227567
O -4.489856 -0.279079 -1.938108
H -4.873535 -1.170619 -1.913058

ENERGIES
SCF ENERGY: -953.183920313
SUM OF ELECTRONIC AND ZERO-POINT ENERGIES: -952.963829
SUM OF ELECTRONIC AND THERMAL ENERGIES: -952.946022
SUM OF ELECTRONIC AND THERMAL ENTHALPIES: -952.945078
SUM OF ELECTRONIC AND THERMAL FREE ENERGIES: -953.011513

==> AOX-I/PE/HIS_4C_HO <==
30
DATASET
C 2.064903 0.407821 -0.013037
C 2.830023 1.575189 0.059899
C 4.218977 1.525041 0.097823
C 4.864916 0.300141 0.062263
C 4.106233 -0.880233 -0.011927
C 2.728459 -0.830900 -0.049256
O 4.842990 -2.027995 -0.043421
O 6.214617 0.240177 0.098056
C 0.609863 0.531238 -0.050403
C -0.280157 -0.472642 -0.112038
C -1.714102 -0.258745 -0.152782
C -2.619934 -1.258746 -0.238539
C -4.016902 -0.933144 -0.254883
C -4.395496 0.417676 -0.321665
C -3.404872 1.454353 -0.211969
O -2.086981 1.044946 -0.093792
O -4.837204 -1.929436 -0.577747
H 2.328648 2.536023 0.087396
H 4.813286 2.429135 0.154592
H 2.173154 -1.761628 -0.106449
H 0.234502 1.550318 -0.026059
H 0.023426 -1.513192 -0.139052
H 4.270685 -2.802973 -0.079423
H 6.488080 -0.686103 0.067795
H -5.423926 0.719435 -0.463933
O -3.615229 2.640217 -0.218639
H -2.305900 -2.292028 -0.267095
H -5.757512 -1.646060 -0.484013
O -4.625126 -0.693656 1.628213
H -3.940452 -0.189238 2.093966

ENERGIES
SCF ENERGY: -953.175052278
SUM OF ELECTRONIC AND ZERO-POINT ENERGIES: -952.954459
SUM OF ELECTRONIC AND THERMAL ENERGIES: -952.937145
SUM OF ELECTRONIC AND THERMAL ENTHALPIES: -952.936200
SUM OF ELECTRONIC AND THERMAL FREE ENERGIES: -953.001031

==> AOX-I/PE/HIS_4OH_HO <==
30
DATASET
C 1.951296 0.497911 0.068645

C	2.765223	1.620245	0.295604
C	4.147848	1.520095	0.285130
C	4.747701	0.291462	0.047038
C	3.945340	-0.842875	-0.181768
C	2.572629	-0.746296	-0.172240
O	4.644300	-1.991823	-0.401414
O	6.090567	0.184403	0.036540
C	0.520603	0.672193	0.094232
C	-0.424731	-0.298935	-0.124731
C	-1.803417	-0.053886	-0.072421
C	-2.770627	-1.080032	-0.297604
C	-4.219449	-0.690130	-0.296195
C	-4.502956	0.645726	-0.407264
C	-3.507861	1.645717	-0.141120
O	-2.214159	1.206110	0.195559
O	-5.004717	-1.676831	-0.123052
H	2.298264	2.580942	0.480657
H	4.777616	2.384083	0.459543
H	1.984320	-1.640248	-0.350998
H	0.169907	1.677462	0.306615
H	-0.141664	-1.323732	-0.340827
H	4.049218	-2.733428	-0.561867
H	6.333274	-0.733149	-0.147886
H	-5.521985	0.996010	-0.498644
O	-3.673100	2.836493	-0.094363
H	-2.490009	-1.831892	-1.028431
H	-4.002936	-2.359148	0.642452
O	-2.973515	-2.331795	0.963600
H	-2.940605	-1.916524	1.840581

ENERGIES

SCF ENERGY: -953.179716477

SUM OF ELECTRONIC AND ZERO-POINT ENERGIES: -952.959801

SUM OF ELECTRONIC AND THERMAL ENERGIES: -952.943278

SUM OF ELECTRONIC AND THERMAL ENTHALPIES: -952.942334

SUM OF ELECTRONIC AND THERMAL FREE ENERGIES: -953.005477

==> AOX-I/PE/HIS_5C_HO <==

30

DATASET

C	1.952036	0.469296	-0.020997
C	2.735456	1.618315	0.132093
C	4.122697	1.545412	0.152969
C	4.749155	0.316358	0.019788
C	3.972494	-0.845443	-0.135437
C	2.596481	-0.774076	-0.155626
O	4.692978	-1.996746	-0.259051
O	6.096434	0.234346	0.040617
C	0.504006	0.614802	-0.035443
C	-0.403884	-0.370643	-0.188719
C	-1.820793	-0.131861	-0.187630
C	-2.751932	-1.136335	-0.318727
C	-4.133461	-0.763500	-0.408793
C	-4.515430	0.532393	-0.274485
C	-3.535485	1.555015	-0.036831
O	-2.201156	1.140484	-0.009176
O	-4.979666	-1.783403	-0.606281
H	2.248019	2.580886	0.237980
H	4.731872	2.433059	0.273810
H	2.026504	-1.689902	-0.273488
H	0.139902	1.630618	0.090246
H	-0.116195	-1.408071	-0.314700
H	4.110301	-2.762813	-0.316009
H	6.356321	-0.692087	-0.050341
H	-5.550733	0.844505	-0.317354
O	-3.732741	2.728123	0.134363
H	-2.436234	-2.134985	-0.583532
H	-5.891942	-1.464218	-0.634212
O	-2.759447	-1.662651	1.820078
H	-3.377064	-2.396610	1.670223

ENERGIES

SCF ENERGY: -953.180790063

SUM OF ELECTRONIC AND ZERO-POINT ENERGIES: -952.961065

SUM OF ELECTRONIC AND THERMAL ENERGIES: -952.943111

SUM OF ELECTRONIC AND THERMAL ENTHALPIES: -952.942166

SUM OF ELECTRONIC AND THERMAL FREE ENERGIES: -953.009008

==> AOX-I/PE/HIS_6C_HO <==

30

DATASET

C	-1.910830	0.431233	-0.037383
C	-2.669194	1.604587	-0.057177
C	-4.058849	1.563443	-0.082661
C	-4.711471	0.341682	-0.088789
C	-3.959228	-0.844889	-0.068339
C	-2.580843	-0.804338	-0.043172
O	-4.702554	-1.989014	-0.075032
O	-6.061820	0.290429	-0.113097
C	-0.454154	0.544712	-0.009925
C	0.428869	-0.464115	-0.012801
C	1.868588	-0.248067	0.004838
C	2.774175	-1.271404	-0.248883
C	4.147212	-0.935904	-0.411503
C	4.547692	0.362345	-0.335234
C	3.589121	1.413620	-0.115986
O	2.251039	1.034626	-0.046432
O	4.973498	-1.969790	-0.627763
H	-2.162131	2.562725	-0.051281
H	-4.648437	2.472260	-0.097417
H	-2.029798	-1.739211	-0.027676
H	-0.070973	1.560995	0.009958
H	0.124331	-1.504912	-0.034369
H	-4.134704	-2.767843	-0.058612
H	-6.340384	-0.634811	-0.111145
H	5.584241	0.659714	-0.427207
O	3.820911	2.589079	-0.022029
H	2.437941	-2.297035	-0.308719
H	5.883168	-1.657890	-0.724357
O	2.401984	-0.634774	1.967300
H	1.825751	-1.375884	2.209332

ENERGIES

SCF ENERGY: -953.177205062

SUM OF ELECTRONIC AND ZERO-POINT ENERGIES: -952.957220

SUM OF ELECTRONIC AND THERMAL ENERGIES: -952.939604

SUM OF ELECTRONIC AND THERMAL ENTHALPIES: -952.938660

SUM OF ELECTRONIC AND THERMAL FREE ENERGIES: -953.004174

==> AOX-I/PE/HIS_7C_HO <==

30

DATASET

C	1.766024	0.479584	-0.129194
C	2.522632	1.640408	-0.333846
C	3.908447	1.589020	-0.398313
C	4.560221	0.374349	-0.244408
C	3.810998	-0.794972	-0.020339
C	2.435650	-0.747455	0.037162
O	4.557149	-1.924839	0.137802
O	5.907071	0.316880	-0.295912
C	0.320950	0.584452	-0.094505
C	-0.552479	-0.460052	-0.114014
C	-1.995766	-0.271034	-0.147510
C	-2.874905	-1.256858	-0.434441
C	-4.266650	-0.937864	-0.425675
C	-4.690789	0.325524	-0.141118
C	-3.742127	1.359482	0.155771
O	-2.401322	0.982090	0.134988
O	-5.092145	-1.953084	-0.712648

H 2.014154 2.590598 -0.451840
H 4.499223 2.482133 -0.562302
H 1.881016 -1.657572 0.242378
H -0.083737 1.591576 -0.042788
H -0.220036 -1.471831 -0.306353
H 3.994149 -2.684481 0.327286
H 6.188843 -0.596388 -0.151061
H -5.738381 0.597133 -0.126005
O -3.974168 2.511886 0.419378
H -2.536083 -2.259268 -0.653329
H -6.011872 -1.657337 -0.682245
O -0.369304 -0.956049 2.038183
H -0.218761 -0.057143 2.374522

ENERGIES
SCF ENERGY: -953.182681165
SUM OF ELECTRONIC AND ZERO-POINT ENERGIES: -952.963039
SUM OF ELECTRONIC AND THERMAL ENERGIES: -952.945198
SUM OF ELECTRONIC AND THERMAL ENTHALPIES: -952.944253
SUM OF ELECTRONIC AND THERMAL FREE ENERGIES: -953.011056

==> AOX-I/PE/HIS_8C_HO <==
30
DATASET
C -1.748742 -0.104112 0.006198
C -2.273821 -1.370005 -0.270366
C -3.642707 -1.591944 -0.224819
C -4.508872 -0.554303 0.095908
C -3.992150 0.719112 0.362942
C -2.628791 0.942472 0.313740
O -4.924560 1.670173 0.660542
O -5.842344 -0.775810 0.141309
C -0.316018 0.185052 -0.018552
C 0.673627 -0.752986 0.047382
C 2.075364 -0.418612 0.112382
C 3.061263 -1.333671 0.282556
C 4.410250 -0.870778 0.339693
C 4.697466 0.456625 0.223383
C 3.645310 1.414127 0.034147
O 2.349997 0.898669 -0.017609
O 5.336551 -1.821961 0.513263
H -1.619087 -2.187861 -0.544971
H -4.060073 -2.567410 -0.444667
H -2.240082 1.936098 0.517890
H 0.450636 -1.813353 0.048562
H -4.507557 2.529662 0.790187
H -6.289164 0.052825 0.358963
H 5.709934 0.838158 0.262038
O 3.754880 2.606402 -0.089054
H 2.831098 -2.385479 0.376325
H 6.220512 -1.431523 0.539764
H -0.041457 1.228208 0.100621
O 0.070145 0.257036 -2.172747
H 0.874810 0.798748 -2.117897

ENERGIES
SCF ENERGY: -953.180150842
SUM OF ELECTRONIC AND ZERO-POINT ENERGIES: -952.960459
SUM OF ELECTRONIC AND THERMAL ENERGIES: -952.942716
SUM OF ELECTRONIC AND THERMAL ENTHALPIES: -952.941771
SUM OF ELECTRONIC AND THERMAL FREE ENERGIES: -953.008381

==> AOX-I/PE/HIS_8C_HOO <==
31
DATASET
C -1.789913 -0.203061 -0.324167
C -2.556637 -1.137758 -1.020660
C -3.943319 -1.039454 -1.053360
C -4.579519 -0.012705 -0.373831
C -3.815735 0.919954 0.344139
C -2.437822 0.829955 0.368577
O -4.540899 1.878981 0.989979
O -5.929068 0.083364 -0.395824
C -0.329460 -0.341851 -0.310373
C 0.524598 0.748819 -0.193380
C 1.943749 0.604258 -0.202971
C 2.831941 1.638414 -0.186115
C 4.223519 1.335678 -0.182532
C 4.660589 0.041403 -0.164489
C 3.717640 -1.036284 -0.133069
O 2.376034 -0.679771 -0.205800
O 5.043118 2.394672 -0.187585
H -2.063632 -1.948122 -1.545535
H -4.545082 -1.758047 -1.596839
H -1.871742 1.553175 0.948205
H 0.146218 1.749705 -0.024334
H -3.965769 2.448491 1.513588
H -6.197213 0.836779 0.146275
H 5.711005 -0.217669 -0.137009
O 3.943886 -2.217805 -0.041887
H 2.487732 2.662792 -0.177511
H 5.966202 2.107703 -0.180718
H 0.056401 -1.226579 -0.804233
O 0.069886 -1.227786 1.397153
O 0.545937 -2.473821 1.121469
H 1.506948 -2.355114 1.031451

ENERGIES
SCF ENERGY: -1028.32902456
SUM OF ELECTRONIC AND ZERO-POINT ENERGIES: -1028.103991
SUM OF ELECTRONIC AND THERMAL ENERGIES: -1028.085663
SUM OF ELECTRONIC AND THERMAL ENTHALPIES: -1028.084719
SUM OF ELECTRONIC AND THERMAL FREE ENERGIES: -1028.152217

==> AOX-I/PE/HIS_9C_HO <==
30
DATASET
C 1.641410 0.441835 -0.122182
C 2.392517 1.470659 0.516233
C 3.732081 1.309859 0.802916
C 4.360740 0.103140 0.503674
C 3.622818 -0.952296 -0.084028
C 2.291888 -0.804175 -0.362471
O 4.356475 -2.074942 -0.322289
O 5.665098 -0.058779 0.770317
C 0.173741 0.542528 -0.071058
C -0.666529 -0.496158 -0.009913
C -2.112846 -0.350799 0.045042
C -2.969584 -1.397676 0.093705
C -4.369511 -1.121715 0.143360
C -4.827137 0.161411 0.147601
C -3.904656 1.259361 0.101698
O -2.554563 0.922158 0.047725
O -5.167796 -2.197332 0.185606
H 1.892439 2.402393 0.753022
H 4.311268 2.100548 1.263823
H 1.745085 -1.618801 -0.825771
H -0.220487 1.553429 -0.070751
H -0.310748 -1.520192 0.013447
H 3.829326 -2.741204 -0.779043
H 5.938792 -0.945573 0.495795
H -5.881354 0.402974 0.185830
O -4.168544 2.434685 0.105588
H -2.605418 -2.414943 0.091607
H -6.094944 -1.926091 0.218018
O 1.691257 1.252287 -1.981456
H 2.631019 1.487002 -2.017364

ENERGIES

SCF ENERGY: -953.174514801
SUM OF ELECTRONIC AND ZERO-POINT ENERGIES: -952.954159
SUM OF ELECTRONIC AND THERMAL ENERGIES: -952.936618
SUM OF ELECTRONIC AND THERMAL ENTHALPIES: -952.935674
SUM OF ELECTRONIC AND THERMAL FREE ENERGIES: -953.000976

==> AOX-I/PE/ISOH_10C_HO <==
30
DATASET

C	-1.981725	-0.369623	-0.090269
C	-2.698106	-1.569283	-0.104483
C	-4.087278	-1.579667	-0.040554
C	-4.782525	-0.384108	0.036501
C	-4.073154	0.828536	0.047775
C	-2.695374	0.838917	-0.015213
O	-4.854462	1.944428	0.121068
O	-6.132939	-0.383817	0.097870
C	-0.522010	-0.424287	-0.154118
C	0.316003	0.622343	-0.097984
C	1.759213	0.512536	-0.165838
C	2.614914	1.550047	-0.090251
C	4.060896	1.361767	-0.172518
C	4.476884	-0.026487	-0.293450
C	3.519519	-1.018317	-0.305333
O	2.209746	-0.774029	-0.341226
O	4.860085	2.289956	-0.143748
H	-2.159131	-2.508113	-0.163460
H	-4.644107	-2.508985	-0.049558
H	-2.178965	1.793436	-0.008813
H	-0.105164	-1.422591	-0.251958
H	-0.045276	1.638545	0.010712
H	2.223727	2.551000	0.035168
H	-4.313294	2.742142	0.139383
H	-6.443865	0.529642	0.149872
H	5.522356	-0.296173	-0.356691
O	3.823301	-2.276767	-0.558415
O	3.720229	-0.870282	1.769807
H	4.625920	-1.164550	1.954246
H	3.066573	-2.844517	-0.347338

ENERGIES
SCF ENERGY: -953.167174893
SUM OF ELECTRONIC AND ZERO-POINT ENERGIES: -952.947013
SUM OF ELECTRONIC AND THERMAL ENERGIES: -952.929488
SUM OF ELECTRONIC AND THERMAL ENTHALPIES: -952.928544
SUM OF ELECTRONIC AND THERMAL FREE ENERGIES: -952.993702

==> AOX-I/PE/ISOH_11C_HO <==
30
DATASET

C	1.444188	0.490963	-0.139804
C	2.163843	1.697516	0.011598
C	3.544530	1.739394	-0.030278
C	4.256428	0.558185	-0.222037
C	3.558465	-0.674131	-0.327706
C	2.157716	-0.682688	-0.348510
O	4.304197	-1.758628	-0.701144
O	5.587010	0.572558	-0.295093
C	-0.018698	0.543625	-0.092385
C	-0.848991	-0.508477	-0.118820
C	-2.296433	-0.394359	-0.069002
C	-3.152277	-1.434940	-0.085042
C	-4.600232	-1.233394	-0.027143
C	-5.000503	0.161241	0.050314
C	-4.058779	1.127187	0.059508
O	-2.744182	0.893890	0.002777
O	-5.398009	-2.163952	-0.041948
H	1.610018	2.618261	0.160584
H	4.085968	2.671701	0.070834
H	1.663423	-1.634483	-0.507119
H	-0.442390	1.541378	-0.022658
H	-0.483866	-1.527937	-0.175540
H	4.272957	-2.390710	0.031801
H	5.892328	-0.338442	-0.425258
H	-6.050370	0.420339	0.098194
O	-4.252448	2.441454	0.124519
H	-5.199907	2.628425	0.166115
H	-2.763546	-2.443239	-0.142461
O	3.920404	-1.131963	1.732947
H	3.184082	-0.712898	2.204886

ENERGIES
SCF ENERGY: -953.166925221
SUM OF ELECTRONIC AND ZERO-POINT ENERGIES: -952.946783
SUM OF ELECTRONIC AND THERMAL ENERGIES: -952.929175
SUM OF ELECTRONIC AND THERMAL ENTHALPIES: -952.928230
SUM OF ELECTRONIC AND THERMAL FREE ENERGIES: -952.993924

==> AOX-I/PE/ISOH_2C_HO <==
30
DATASET

C	-1.981725	-0.369623	-0.090269
C	-2.698106	-1.569283	-0.104483
C	-4.087278	-1.579667	-0.040554
C	-4.782525	-0.384108	0.036501
C	-4.073154	0.828536	0.047775
C	-2.695374	0.838917	-0.015213
O	-4.854462	1.944428	0.121068
O	-6.132939	-0.383817	0.097870
C	-0.522010	-0.424287	-0.154118
C	0.316003	0.622343	-0.097984
C	1.759213	0.512536	-0.165838
C	2.614914	1.550047	-0.090251
C	4.060896	1.361767	-0.172518
C	4.476884	-0.026487	-0.293450
C	3.519519	-1.018317	-0.305333
O	2.209746	-0.774029	-0.341226
O	4.860085	2.289956	-0.143748
H	-2.159131	-2.508113	-0.163460
H	-4.644107	-2.508985	-0.049558
H	-2.178965	1.793436	-0.008813
H	-0.105164	-1.422591	-0.251958
H	-0.045276	1.638545	0.010712
H	2.223727	2.551000	0.035168
H	-4.313294	2.742142	0.139383
H	-6.443865	0.529642	0.149872
H	5.522356	-0.296173	-0.356691
O	3.823301	-2.276767	-0.558415
O	3.720229	-0.870282	1.769807
H	4.625920	-1.164550	1.954246
H	3.066573	-2.844517	-0.347338

ENERGIES
SCF ENERGY: -953.167174893
SUM OF ELECTRONIC AND ZERO-POINT ENERGIES: -952.947013
SUM OF ELECTRONIC AND THERMAL ENERGIES: -952.929488
SUM OF ELECTRONIC AND THERMAL ENTHALPIES: -952.928544
SUM OF ELECTRONIC AND THERMAL FREE ENERGIES: -952.993702

==> AOX-I/PE/ISOH_2OH_HOO <==
31
DATASET

C	2.343890	-0.333468	-0.132068
C	2.967242	-1.564021	-0.358705
C	4.348558	-1.697472	-0.275462
C	5.129226	-0.596912	0.038512
C	4.513703	0.644975	0.270768
C	3.143751	0.777808	0.187264

O 5.374767 1.658130 0.575163
O 6.472370 -0.716649 0.121512
C 0.889075 -0.258480 -0.236910
C 0.129879 0.837182 -0.069678
C -1.314419 0.836125 -0.192050
C -2.093877 1.929219 -0.030648
C -3.538272 1.863537 -0.193538
C -4.036236 0.537900 -0.544720
C -3.172970 -0.531492 -0.662121
O -1.842451 -0.387218 -0.484553
O -4.281762 2.829510 -0.064245
H 2.360821 -2.428268 -0.605066
H 4.833756 -2.649918 -0.452351
H 2.700275 1.750838 0.372819
H 0.396615 -1.196540 -0.477398
H 0.558735 1.804463 0.166362
H 4.899585 2.487634 0.700156
H 6.851453 0.144757 0.340841
H -5.083937 0.392368 -0.766472
O -3.522814 -1.734587 -0.921889
H -4.245777 -2.138180 -0.125314
H -1.633137 2.879336 0.205723
O -4.822837 -2.364278 0.927463
O -5.218969 -1.128622 1.307028
H -4.762121 -0.966196 2.153006

ENERGIES
SCF ENERGY: -1028.31342209
SUM OF ELECTRONIC AND ZERO-POINT ENERGIES: -1028.092971
SUM OF ELECTRONIC AND THERMAL ENERGIES: -1028.074352
SUM OF ELECTRONIC AND THERMAL ENTHALPIES: -1028.073408
SUM OF ELECTRONIC AND THERMAL FREE ENERGIES: -1028.142850

==> AOX-I/PE/ISOH_3C_HO <==
30
DATASET
C -2.057589 -0.381308 -0.052374
C -2.776454 -1.579765 -0.041136
C -4.165395 -1.585582 0.022842
C -4.857545 -0.386829 0.076060
C -4.145454 0.824506 0.064382
C -2.767834 0.830565 0.000683
O -4.924754 1.942891 0.118860
O -6.207587 -0.382044 0.137703
C -0.599387 -0.443239 -0.118937
C 0.244482 0.601291 -0.118467
C 1.685229 0.472825 -0.187405
C 2.560249 1.499576 -0.187191
C 3.996730 1.279677 -0.265726
C 4.399479 -0.132263 -0.256485
C 3.409458 -1.090167 -0.346120
O 2.116962 -0.827636 -0.259660
O 4.817823 2.186572 -0.307471
O 3.608357 -2.387443 -0.426443
H -2.238990 -2.520417 -0.081427
H -4.724380 -2.513566 0.032527
H -2.248692 1.783579 -0.008659
H -0.186175 -1.446565 -0.170938
H -0.108002 1.624737 -0.062490
H 2.186304 2.513886 -0.142688
H 4.551400 -2.580177 -0.315704
H -4.382396 2.739981 0.119699
H -6.516555 0.532918 0.172379
H 5.406949 -0.388401 -0.554927
O 4.773802 -0.478111 1.690683
H 5.227901 0.364462 1.842098

ENERGIES
SCF ENERGY: -953.166965665
SUM OF ELECTRONIC AND ZERO-POINT ENERGIES: -952.946529
SUM OF ELECTRONIC AND THERMAL ENERGIES: -952.928670
SUM OF ELECTRONIC AND THERMAL ENTHALPIES: -952.927726
SUM OF ELECTRONIC AND THERMAL FREE ENERGIES: -952.994308

==> AOX-I/PE/ISOH_7C_HO <==
30
DATASET
C -1.747005 0.461969 -0.142185
C -2.486287 1.632209 -0.354636
C -3.873241 1.602334 -0.411001
C -4.543477 0.399702 -0.243635
C -3.811610 -0.779593 -0.014297
C -2.435297 -0.752789 0.037085
O -4.574374 -1.896650 0.153214
O -5.891245 0.363278 -0.288929
C -0.299232 0.544877 -0.113552
C 0.556123 -0.513374 -0.096869
C 2.003467 -0.361328 -0.150222
C 2.869867 -1.348724 -0.438742
C 4.312680 -1.106576 -0.474419
C 4.689026 0.264228 -0.173848
C 3.733936 1.175456 0.104130
O 2.424867 0.907519 0.120862
O 5.124608 -1.986288 -0.737286
H -1.964015 2.573377 -0.484330
H -4.450488 2.503260 -0.580384
H -1.894530 -1.671299 0.241453
H 0.117841 1.548091 -0.097097
H -4.022397 -2.665574 0.337979
H -6.186913 -0.544922 -0.139830
H 5.732996 0.549525 -0.170835
O 3.907129 2.460768 0.399887
H 4.851005 2.669148 0.399405
H 2.496111 -2.343219 -0.644373
H 0.204393 -1.525913 -0.245915
O 0.409894 -0.896156 2.083278
H 0.095251 -0.022591 2.368860

ENERGIES
SCF ENERGY: -953.168041114
SUM OF ELECTRONIC AND ZERO-POINT ENERGIES: -952.948195
SUM OF ELECTRONIC AND THERMAL ENERGIES: -952.930314
SUM OF ELECTRONIC AND THERMAL ENTHALPIES: -952.929369
SUM OF ELECTRONIC AND THERMAL FREE ENERGIES: -952.995982

==> AOX-I/WATER/HIS(-)_C10_OH <==
29
DATASET
C -1.532126 0.566647 -0.125531
C -2.276517 1.719212 0.100129
C -3.673128 1.687297 0.104805
C -4.349346 0.502091 -0.127095
C -3.620861 -0.668312 -0.376267
C -2.223611 -0.654499 -0.333652
O -4.346358 -1.786828 -0.619135
O -5.714236 0.488550 -0.127383
C -0.075074 0.638998 -0.097267
C 0.763117 -0.408927 -0.138451
C 2.212086 -0.284670 -0.100716
C 3.056687 -1.336671 -0.118127
C 4.499324 -1.130024 -0.076896
C 4.925067 0.213855 -0.015352
C 4.028102 1.286021 0.003994
O 2.664090 0.995324 -0.042594
O 5.289570 -2.117394 -0.095906
H -1.764293 2.657804 0.277093
H -4.248612 2.586882 0.289446
H 0.336195 1.641012 -0.018418
H 0.400055 -1.429733 -0.194904

H -3.764903 -2.544928 -0.767140
H -6.028378 -0.412573 -0.285066
H 5.981020 0.448039 0.019424
O 4.279555 2.496983 0.058401
H 2.658203 -2.342134 -0.163837
H -1.698495 -1.549661 -0.644831
O -2.143799 -1.473892 1.723105
H -2.503684 -0.662751 2.118885

ENERGIES
SCF ENERGY: -952.738070345
SUM OF ELECTRONIC AND ZERO-POINT ENERGIES: -952.532243
SUM OF ELECTRONIC AND THERMAL ENERGIES: -952.514614
SUM OF ELECTRONIC AND THERMAL ENTHALPIES: -952.513670
SUM OF ELECTRONIC AND THERMAL FREE ENERGIES: -952.580001

==> AOX-I/WATER/HIS(-)_C11_OH <==
29
DATASET
C 1.426030 0.510747 -0.141174
C 2.179823 1.676546 0.153546
C 3.557266 1.667574 0.154593
C 4.238639 0.484243 -0.138662
C 3.499645 -0.700200 -0.421265
C 2.097150 -0.655749 -0.464936
O 4.224634 -1.743548 -0.869868
O 5.577159 0.483063 -0.192360
C -0.035838 0.602442 -0.103092
C -0.873895 -0.443395 -0.063840
C -2.323593 -0.321395 -0.029490
C -3.161144 -1.375248 0.052200
C -4.605273 -1.174013 0.085200
C -5.038207 0.167355 0.026251
C -4.147537 1.241897 -0.060432
O -2.781525 0.956202 -0.084965
O -5.387830 -2.164263 0.162901
H 1.650150 2.594393 0.382372
H 4.132535 2.559570 0.369398
H 1.571197 -1.562304 -0.745036
H -0.441471 1.609569 -0.083095
H -0.506945 -1.464223 -0.040834
H 3.660614 -2.513794 -1.024740
H 5.903350 -0.406102 -0.399588
H -6.095277 0.398288 0.044911
O -4.406396 2.450623 -0.121811
H -2.756637 -2.378470 0.094429
O 3.869514 -1.043866 1.728448
H 3.239643 -0.427448 2.130033

ENERGIES
SCF ENERGY: -952.738473754
SUM OF ELECTRONIC AND ZERO-POINT ENERGIES: -952.531593
SUM OF ELECTRONIC AND THERMAL ENERGIES: -952.514521
SUM OF ELECTRONIC AND THERMAL ENTHALPIES: -952.513577
SUM OF ELECTRONIC AND THERMAL FREE ENERGIES: -952.578139

==> AOX-I/WATER/HIS(-)_C12_OH <==
29
DATASET
C 1.407881 0.381231 -0.224883
C 2.155202 1.571967 -0.303668
C 3.534265 1.547740 -0.373323
C 4.211112 0.325118 -0.337911
C 3.467501 -0.876256 -0.274279
C 2.084806 -0.844116 -0.214165
O 4.205580 -2.014585 -0.291833
O 5.555374 0.300452 -0.494603
C -0.049774 0.487424 -0.159077
C -0.918076 -0.534584 -0.109477
C -2.361267 -0.360459 -0.046105
C -3.240089 -1.383502 -0.012761
C -4.674387 -1.127664 0.047551
C -5.054335 0.230642 0.072088
C -4.122320 1.272418 0.039884
O -2.769819 0.935046 -0.022840
O -5.496281 -2.088675 0.074249
H 1.633152 2.521646 -0.316336
H 4.115578 2.459278 -0.443614
H 1.552397 -1.787667 -0.161091
H -0.436097 1.502321 -0.153838
H -0.589502 -1.568382 -0.116623
H 3.636546 -2.791806 -0.213970
H 5.874847 -0.608835 -0.402623
H -6.101093 0.501200 0.118951
O -4.333629 2.491821 0.059477
H -2.876089 -2.402743 -0.032807
O 4.241430 0.083858 2.038904
H 3.325900 0.371899 2.191872

ENERGIES
SCF ENERGY: -952.739007247
SUM OF ELECTRONIC AND ZERO-POINT ENERGIES: -952.533343
SUM OF ELECTRONIC AND THERMAL ENERGIES: -952.515912
SUM OF ELECTRONIC AND THERMAL ENTHALPIES: -952.514968
SUM OF ELECTRONIC AND THERMAL FREE ENERGIES: -952.580652

==> AOX-I/WATER/HIS(-)_C13_OH <==
29
DATASET
C -1.432096 0.255586 -0.213664
C -2.216772 1.379141 -0.405170
C -3.624397 1.287133 -0.409984
C -4.228483 0.020121 -0.341604
C -3.432472 -1.121865 -0.129980
C -2.064914 -1.005905 -0.066681
O -4.118976 -2.297138 -0.014234
O -5.564588 -0.074353 -0.441221
C 0.024225 0.406543 -0.179146
C 0.918133 -0.580538 -0.020734
C 2.358180 -0.370335 0.005593
C 3.257161 -1.364584 0.155935
C 4.686323 -1.077663 0.168778
C 5.040351 0.279989 0.021621
C 4.087925 1.291587 -0.134455
O 2.741289 0.925412 -0.136918
O 5.525836 -2.014223 0.303638
H -1.752013 2.351043 -0.521392
H -1.481818 -1.906314 0.093689
H 0.382098 1.425126 -0.297021
H 0.616804 -1.615601 0.098765
H -3.507549 -3.034019 0.116044
H -5.840022 -0.995711 -0.319996
H 6.082022 0.573183 0.020958
O 4.276478 2.506872 -0.276689
H 2.912993 -2.385021 0.265803
H -4.235189 2.132132 -0.697937
O -4.116880 1.687006 1.634227
H -3.361575 1.191315 1.987610

ENERGIES
SCF ENERGY: -952.736010893
SUM OF ELECTRONIC AND ZERO-POINT ENERGIES: -952.529396
SUM OF ELECTRONIC AND THERMAL ENERGIES: -952.512074
SUM OF ELECTRONIC AND THERMAL ENTHALPIES: -952.511130
SUM OF ELECTRONIC AND THERMAL FREE ENERGIES: -952.576747

==> AOX-I/WATER/HIS(-)_C14_OH <==
29

DATASET
C -1.558706 0.234137 -0.212882
C -2.344556 1.394952 -0.394400
C -3.743756 1.309921 -0.470050
C -4.366845 0.097743 -0.288561
C -3.587883 -1.061249 -0.060368
C -2.208400 -0.992533 -0.027348
O -4.292621 -2.208438 0.111627
O -5.724954 0.009466 -0.336796
C -0.106119 0.378278 -0.200839
C 0.780250 -0.618234 -0.046898
C 2.221660 -0.425010 -0.041224
C 3.108902 -1.429112 0.114153
C 4.542232 -1.160709 0.110519
C 4.911493 0.189836 -0.059448
C 3.970473 1.211976 -0.220963
O 2.619195 0.863601 -0.205543
O 5.369993 -2.106265 0.252802
H -4.343845 2.194057 -0.647734
H -1.653015 -1.908744 0.140626
H 0.258732 1.392849 -0.330231
H 0.467135 -1.648397 0.084840
H -3.697085 -2.955755 0.259103
H -5.993836 -0.910107 -0.201179
H 5.956683 0.469869 -0.073652
O 4.173794 2.422038 -0.382022
H 2.752335 -2.443204 0.241932
H -1.853995 2.330973 -0.628717
O -2.032959 1.961716 1.793623
H -2.505165 1.161525 2.075349

ENERGIES
SCF ENERGY: -952.737777657
SUM OF ELECTRONIC AND ZERO-POINT ENERGIES: -952.532211
SUM OF ELECTRONIC AND THERMAL ENERGIES: -952.514580
SUM OF ELECTRONIC AND THERMAL ENTHALPIES: -952.513636
SUM OF ELECTRONIC AND THERMAL FREE ENERGIES: -952.580051

==> AOX-I/WATER/HIS(-)_C5_OH <==
29
DATASET
C -1.895286 0.462738 -0.063332
C -2.662671 1.624973 0.060489
C -4.051919 1.566454 0.094464
C -4.692383 0.341351 0.007208
C -3.933838 -0.833053 -0.116810
C -2.554713 -0.774496 -0.153896
O -4.655777 -1.994330 -0.191663
O -6.055629 0.284118 0.047381
C -0.438061 0.588367 -0.089241
C 0.446859 -0.412307 -0.224713
C 1.883984 -0.209874 -0.240902
C 2.781413 -1.217064 -0.405197
C 4.213362 -0.935384 -0.458188
C 4.578807 0.414098 -0.276141
C 3.635812 1.427194 -0.077498
O 2.282010 1.070570 -0.074120
O 5.038368 -1.874474 -0.643668
H -2.164442 2.585074 0.132204
H -4.650120 2.464742 0.191079
H -2.002304 -1.703429 -0.249538
H -0.064151 1.603422 0.011364
H 0.136386 -1.445783 -0.334116
H -4.064103 -2.756065 -0.240699
H -6.337925 -0.638608 -0.022014
H 5.621706 0.702188 -0.289591
O 3.831102 2.634467 0.099357
H 2.426196 -2.227628 -0.559938
O 2.528102 -1.292845 2.130677
H 3.485146 -1.461949 2.080081

ENERGIES
SCF ENERGY: -952.740355804
SUM OF ELECTRONIC AND ZERO-POINT ENERGIES: -952.534961
SUM OF ELECTRONIC AND THERMAL ENERGIES: -952.517075
SUM OF ELECTRONIC AND THERMAL ENTHALPIES: -952.516131
SUM OF ELECTRONIC AND THERMAL FREE ENERGIES: -952.583662

==> AOX-I/WATER/HIS(-)_C6_OH <==
29
DATASET
C -1.866105 0.441228 -0.036321
C -2.618154 1.618381 -0.084471
C -4.008152 1.579621 -0.118640
C -4.663199 0.359410 -0.104703
C -3.919283 -0.830073 -0.054292
C -2.539441 -0.791572 -0.019788
O -4.657260 -1.983363 -0.043253
O -6.026735 0.320750 -0.140534
C -0.406878 0.548240 -0.005634
C 0.465291 -0.468900 0.009301
C 1.908691 -0.269577 0.022609
C 2.797638 -1.284306 -0.309372
C 4.215200 -0.999765 -0.503787
C 4.584393 0.351006 -0.363512
C 3.659319 1.367599 -0.084288
O 2.307231 1.012829 0.056889
O 5.011931 -1.939107 -0.779323
H -2.107795 2.574652 -0.096155
H -4.595564 2.489307 -0.156478
H -1.998100 -1.731097 0.019478
H -0.021875 1.564115 -0.003330
H 0.153523 -1.507269 0.001691
H -4.076241 -2.754207 -0.016421
H -6.319473 -0.601090 -0.118815
H 5.618226 0.648007 -0.480371
O 3.867648 2.571764 0.050559
H 2.425771 -2.294570 -0.415876
O 2.255935 -0.910241 1.991420
H 3.195606 -0.696665 2.097597

ENERGIES
SCF ENERGY: -952.737787466
SUM OF ELECTRONIC AND ZERO-POINT ENERGIES: -952.530772
SUM OF ELECTRONIC AND THERMAL ENERGIES: -952.513887
SUM OF ELECTRONIC AND THERMAL ENTHALPIES: -952.512943
SUM OF ELECTRONIC AND THERMAL FREE ENERGIES: -952.576501

==> AOX-I/WATER/HIS(-)_C7_OH <==
29
DATASET
C -1.728160 0.415669 -0.149196
C -2.471575 1.598440 -0.228017
C -3.860988 1.570006 -0.246800
C -4.525185 0.355408 -0.185978
C -3.790380 -0.839284 -0.105833
C -2.411011 -0.811767 -0.085036
O -4.538262 -1.984199 -0.054245
O -5.887284 0.326909 -0.207235
C -0.275412 0.512619 -0.133327
C 0.599011 -0.521248 -0.079210
C 2.043511 -0.351420 -0.135685
C 2.915082 -1.374746 -0.235173
C 4.350931 -1.120925 -0.283125
C 4.735980 0.234680 -0.218825
C 3.808996 1.275993 -0.110387
O 2.454956 0.940772 -0.070666
O 5.167544 -2.081804 -0.374476
H -1.952825 2.548979 -0.276309

H	-4.442032	2.482350	-0.308937
H	-1.876913	-1.753992	-0.020572
H	0.122545	1.522727	-0.174593
H	-3.963969	-2.760222	-0.029231
H	-6.188194	-0.591282	-0.155196
H	5.783847	0.503393	-0.247525
O	4.025484	2.492333	-0.040404
H	2.545724	-2.391283	-0.279563
H	0.263613	-1.552036	-0.074959
O	0.337934	-0.225333	2.305509
H	0.880414	0.581406	2.278566

ENERGIES
SCF ENERGY: -952.740096250
SUM OF ELECTRONIC AND ZERO-POINT ENERGIES: -952.534179
SUM OF ELECTRONIC AND THERMAL ENERGIES: -952.516657
SUM OF ELECTRONIC AND THERMAL ENTHALPIES: -952.515713
SUM OF ELECTRONIC AND THERMAL FREE ENERGIES: -952.581936

==> AOX-I/WATER/HIS(-)_C8_OH <==
29
DATASET

C	-1.728147	0.415677	-0.149214
C	-2.471503	1.598453	-0.228363
C	-3.860923	1.570085	-0.247247
C	-4.525181	0.355542	-0.186163
C	-3.790430	-0.839162	-0.105613
C	-2.411062	-0.811706	-0.084752
O	-4.538366	-1.984018	-0.053590
O	-5.887283	0.327121	-0.207473
C	-0.275384	0.512617	-0.133287
C	0.599046	-0.521231	-0.079398
C	2.043536	-0.351346	-0.135843
C	2.915145	-1.374602	-0.235775
C	4.350982	-1.120714	-0.283627
C	4.735992	0.234867	-0.218697
C	3.808976	1.276098	-0.109781
O	2.454944	0.940830	-0.070322
O	5.167623	-2.081531	-0.375411
H	-1.952706	2.548957	-0.276830
H	-4.441913	2.482443	-0.309667
H	-1.877044	-1.753958	-0.020029
H	-3.964117	-2.760091	-0.029255
H	-6.188270	-0.591044	-0.155453
H	5.783853	0.503622	-0.247226
O	4.025432	2.492419	-0.039174
H	2.545820	-2.391132	-0.280569
H	0.263658	-1.552024	-0.075591
H	0.122526	1.522758	-0.174328
O	0.338041	-0.227055	2.305522
H	0.878784	0.580876	2.279089

ENERGIES
SCF ENERGY: -952.740096410
SUM OF ELECTRONIC AND ZERO-POINT ENERGIES: -952.534178
SUM OF ELECTRONIC AND THERMAL ENERGIES: -952.516657
SUM OF ELECTRONIC AND THERMAL ENTHALPIES: -952.515713
SUM OF ELECTRONIC AND THERMAL FREE ENERGIES: -952.581930

==> AOX-I/WATER/HIS(-)_C9_OH <==
29
DATASET

C	1.590631	-0.411811	0.062667
C	2.349799	-1.484039	-0.494356
C	3.699390	-1.355389	-0.732881
C	4.333134	-0.143557	-0.459230
C	3.589421	0.955555	0.045125
C	2.246621	0.835923	0.271609
O	4.322180	2.084255	0.261344
O	5.653337	-0.022362	-0.676613
C	0.126019	-0.512830	0.008437
C	-0.717402	0.525554	-0.021942
C	-2.167401	0.386188	-0.064337
C	-3.019341	1.430414	-0.093812
C	-4.460953	1.209784	-0.126170
C	-4.874427	-0.138880	-0.127095
C	-3.968177	-1.203360	-0.099560
O	-2.606475	-0.898866	-0.066607
O	-5.258931	2.190438	-0.150755
H	1.843203	-2.418199	-0.706263
H	4.286916	-2.173571	-1.130519
H	1.699951	1.686293	0.664121
H	-0.263746	-1.525597	-0.018549
H	-0.362334	1.550398	-0.022487
H	3.773123	2.779129	0.648363
H	5.946953	0.869944	-0.436825
H	-5.928270	-0.383749	-0.150700
O	-4.209445	-2.417285	-0.099597
H	-2.629246	2.440182	-0.090830
O	1.677568	-1.207061	2.002612
H	2.643699	-1.219103	2.069901

ENERGIES
SCF ENERGY: -952.734821217
SUM OF ELECTRONIC AND ZERO-POINT ENERGIES: -952.528667
SUM OF ELECTRONIC AND THERMAL ENERGIES: -952.511305
SUM OF ELECTRONIC AND THERMAL ENTHALPIES: -952.510361
SUM OF ELECTRONIC AND THERMAL FREE ENERGIES: -952.576206

==> AOX-I/WATER/HIS(2-)_11OH_HOO <==
30
DATASET

C	-1.031570	0.669717	-0.237187
C	-1.720380	1.893638	-0.057022
C	-3.093919	1.980977	-0.167270
C	-3.829708	0.838020	-0.474518
C	-3.162809	-0.408631	-0.658474
C	-1.770420	-0.469884	-0.524094
O	-3.871337	-1.485345	-0.979572
O	-5.166587	0.823268	-0.603638
C	0.427107	0.676414	-0.105125
C	1.231375	-0.389542	-0.231926
C	2.678694	-0.331586	-0.098060
C	3.484338	-1.405093	-0.230405
C	4.929825	-1.270427	-0.091624
C	5.399442	0.029641	0.190035
C	4.541410	1.125899	0.324484
O	3.171688	0.905159	0.171978
O	5.680303	-2.279281	-0.225348
H	-1.148180	2.785097	0.175144
H	-3.611928	2.922895	-0.025406
H	-1.302326	-1.436702	-0.667456
H	0.867884	1.644472	0.113463
H	0.841539	-1.378497	-0.448772
H	-5.530963	1.706163	-0.445689
H	6.459719	0.211213	0.307780
O	4.833573	2.303786	0.567088
H	3.052727	-2.374181	-0.445890
H	-4.531365	-1.726911	-0.154527
O	-5.076958	-1.844338	1.051476
O	-4.401609	-0.908683	1.771285
H	-4.950013	-0.104931	1.732313

ENERGIES
SCF ENERGY: -1027.88425716
SUM OF ELECTRONIC AND ZERO-POINT ENERGIES: -1027.676794
SUM OF ELECTRONIC AND THERMAL ENERGIES: -1027.658897
SUM OF ELECTRONIC AND THERMAL ENTHALPIES: -1027.657953
SUM OF ELECTRONIC AND THERMAL FREE ENERGIES: -

1027.725949

==> AOX-I/WATER/HIS(2-)_12OH_HOO <==
30
DATASET
C -0.993286 0.547713 -0.343025
C -1.711271 1.763639 -0.282255
C -3.083706 1.781979 -0.382274
C -3.796214 0.586220 -0.571556
C -3.073526 -0.638625 -0.638829
C -1.695311 -0.650716 -0.521226
O -3.819926 -1.755765 -0.799367
O -5.115873 0.589035 -0.723206
C 0.457928 0.604583 -0.204462
C 1.291827 -0.449031 -0.232419
C 2.732687 -0.339948 -0.078119
C 3.567852 -1.399088 -0.108106
C 5.005853 -1.214556 0.056616
C 5.433297 0.115923 0.245742
C 4.544748 1.196299 0.274056
O 3.186041 0.927297 0.104143
O 5.785834 -2.208817 0.025984
H -1.163941 2.689223 -0.146803
H -3.644322 2.707640 -0.331192
H -1.182766 -1.605089 -0.568538
H 0.871564 1.598401 -0.063333
H 0.931589 -1.462684 -0.371887
H -5.566505 0.030132 0.072180
H 6.484799 0.334863 0.377889
O 4.801097 2.395709 0.434587
H 3.166083 -2.393489 -0.255278
H -3.252347 -2.538835 -0.816933
O -5.818923 -0.523354 1.281747
O -4.582703 -0.591672 1.835336
H -4.213801 -1.445967 1.545247

ENERGIES
SCF ENERGY: -1027.88602482
SUM OF ELECTRONIC AND ZERO-POINT ENERGIES: -1027.678553
SUM OF ELECTRONIC AND THERMAL ENERGIES: -1027.660659
SUM OF ELECTRONIC AND THERMAL ENTHALPIES: -1027.659714
SUM OF ELECTRONIC AND THERMAL FREE ENERGIES: -1027.726835

==> AOX-I/WATER/HIS(2-)_C10_OH <==
28
DATASET
C -1.544380 0.523937 -0.160985
C -2.297660 1.691496 0.008786
C -3.691496 1.710057 0.030546
C -4.433006 0.527777 -0.149120
C -3.648355 -0.682044 -0.349015
C -2.244269 -0.703659 -0.266120
O -4.326299 -1.804522 -0.586821
O -5.705324 0.411735 -0.183424
C -0.097286 0.589288 -0.123305
C 0.757356 -0.453567 -0.149984
C 2.200552 -0.314533 -0.104781
C 3.067935 -1.352233 -0.145963
C 4.502229 -1.123865 -0.093231
C 4.911137 0.225319 0.006169
C 3.998275 1.280348 0.051170
O 2.639686 0.970384 -0.012108
O 5.313587 -2.096171 -0.134405
H -1.763733 2.630023 0.127554
H -4.227076 2.642259 0.168075
H 0.315793 1.592634 -0.056322
H 0.404349 -1.477917 -0.205481
H -5.272088 -1.563975 -0.539868
H 5.963672 0.472295 0.053897
O 4.229208 2.495332 0.142976
H 2.687292 -2.362901 -0.219680
H -1.738117 -1.622227 -0.534031
O -2.495062 -1.392536 1.746118
H -2.502656 -0.493886 2.102177

ENERGIES
SCF ENERGY: -952.277265555
SUM OF ELECTRONIC AND ZERO-POINT ENERGIES: -952.083449
SUM OF ELECTRONIC AND THERMAL ENERGIES: -952.066834
SUM OF ELECTRONIC AND THERMAL ENTHALPIES: -952.065889
SUM OF ELECTRONIC AND THERMAL FREE ENERGIES: -952.129791

==> AOX-I/WATER/HIS(2-)_C11_OH <==
28
DATASET
C 1.463223 0.440893 -0.123331
C 2.201393 1.667414 -0.084354
C 3.565835 1.703014 -0.187259
C 4.316909 0.493482 -0.318763
C 3.558533 -0.759503 -0.179273
C 2.151601 -0.755544 -0.226733
O 4.213724 -1.893921 -0.502686
O 5.555869 0.427349 -0.526409
C 0.007730 0.531743 -0.075731
C -0.853517 -0.500830 -0.106383
C -2.297715 -0.349383 -0.061118
C -3.165214 -1.383520 -0.105444
C -4.602165 -1.149336 -0.057089
C -5.002030 0.200924 0.038806
C -4.083879 1.253510 0.087451
O -2.726243 0.937517 0.030930
O -5.413370 -2.119661 -0.099770
H 1.644167 2.594757 -0.000271
H 4.105926 2.642613 -0.211912
H 1.643474 -1.711668 -0.277712
H -0.392195 1.539632 -0.010368
H -0.510364 -1.527971 -0.170388
H 5.159659 -1.671167 -0.533274
H -6.053185 0.454492 0.082096
O -4.311448 2.467614 0.177036
H -2.787513 -2.395360 -0.178380
O 3.957277 -0.840815 1.860342
H 3.419334 -0.107163 2.179974

ENERGIES
SCF ENERGY: -952.291872424
SUM OF ELECTRONIC AND ZERO-POINT ENERGIES: -952.097651
SUM OF ELECTRONIC AND THERMAL ENERGIES: -952.080959
SUM OF ELECTRONIC AND THERMAL ENTHALPIES: -952.080014
SUM OF ELECTRONIC AND THERMAL FREE ENERGIES: -952.143922

==> AOX-I/WATER/HIS(2-)_C12_OH <==
28
DATASET
C 1.416198 0.366134 -0.174339
C 2.187813 1.576952 -0.203534
C 3.542456 1.570195 -0.243748
C 4.290196 0.310663 -0.216756
C 3.450252 -0.914318 -0.257645
C 2.089371 -0.882466 -0.227533
O 4.133384 -2.073018 -0.338196
O 5.484658 0.213434 -0.610594
C -0.012151 0.484289 -0.119801
C -0.898802 -0.542902 -0.067045
C -2.332372 -0.363198 -0.020002
C -3.218014 -1.384967 0.040156
C -4.650051 -1.125843 0.082052
C -5.028708 0.233327 0.059100
C -4.095122 1.272476 -0.003677

O -2.742792 0.933840 -0.041732
O -5.475182 -2.084259 0.133837
H 1.647996 2.518346 -0.210459
H 4.120904 2.486172 -0.285089
H 1.541405 -1.816724 -0.257500
H -0.399709 1.498847 -0.118854
H -0.573177 -1.577016 -0.055646
H 5.072783 -1.833402 -0.434241
H -6.075518 0.506259 0.087306
O -4.306930 2.491884 -0.032576
H -2.855483 -2.404838 0.055733
O 4.419950 0.217324 1.821776
H 4.969707 -0.573331 1.875271

ENERGIES
SCF ENERGY: -952.290200456
SUM OF ELECTRONIC AND ZERO-POINT ENERGIES: -952.096801
SUM OF ELECTRONIC AND THERMAL ENERGIES: -952.080005
SUM OF ELECTRONIC AND THERMAL ENTHALPIES: -952.079061
SUM OF ELECTRONIC AND THERMAL FREE ENERGIES: -952.143656

==> AOX-I/WATER/HIS(2-)_C13_OH <==
28
DATASET
C -1.468483 0.230737 -0.175133
C -2.252094 1.350003 -0.396468
C -3.662265 1.273950 -0.382054
C -4.301168 -0.020849 -0.369102
C -3.445221 -1.162854 -0.101690
C -2.093827 -1.047465 -0.002724
O -4.067793 -2.372840 0.010095
O -5.539347 -0.200155 -0.529220
C -0.019855 0.386132 -0.150996
C 0.884766 -0.594952 0.017969
C 2.321420 -0.375888 0.026278
C 3.231375 -1.362887 0.171651
C 4.657314 -1.066547 0.161201
C 5.001065 0.292641 0.000135
C 4.039867 1.296391 -0.149498
O 2.696153 0.921680 -0.130833
O 5.506480 -1.996310 0.288532
H -1.776237 2.314417 -0.535738
H -1.504113 -1.937631 0.182332
H 0.336186 1.403247 -0.288975
H 0.589642 -1.629320 0.155502
H -5.016991 -2.205228 -0.110437
H 6.040756 0.592270 -0.017986
O 4.218664 2.512353 -0.302715
H 2.896146 -2.384980 0.293128
H -4.253283 2.103795 -0.745894
O -4.207199 1.796982 1.562652
H -3.525120 1.259271 1.982577

ENERGIES
SCF ENERGY: -952.287999444
SUM OF ELECTRONIC AND ZERO-POINT ENERGIES: -952.093579
SUM OF ELECTRONIC AND THERMAL ENERGIES: -952.076772
SUM OF ELECTRONIC AND THERMAL ENTHALPIES: -952.075828
SUM OF ELECTRONIC AND THERMAL FREE ENERGIES: -952.140334

==> AOX-I/WATER/HIS(2-)_C14_OH <==
28
DATASET
C -1.586655 0.198371 -0.105143
C -2.396075 1.380351 -0.336194
C -3.768784 1.269726 -0.580507
C -4.436803 0.057800 -0.364997
C -3.600747 -1.094290 0.012713
C -2.233206 -1.027219 0.117466
O -4.262880 -2.245721 0.223564
O -5.685024 -0.160546 -0.485678
C -0.155184 0.357412 -0.096482
C 0.749943 -0.639712 0.032288
C 2.183544 -0.436001 0.002436
C 3.084367 -1.438968 0.101354
C 4.513859 -1.161872 0.049713
C 4.869492 0.195058 -0.098372
C 3.917854 1.215329 -0.199577
O 2.570268 0.859543 -0.142728
O 5.353016 -2.105201 0.132123
H -4.345186 2.141206 -0.867321
H -1.680936 -1.928433 0.356442
H 0.204768 1.373762 -0.225353
H 0.445363 -1.673634 0.151981
H -5.204090 -2.041699 0.062810
H 5.911927 0.481542 -0.146656
O 4.110213 2.429939 -0.338684
H 2.738201 -2.458452 0.214000
H -1.874679 2.265740 -0.675559
O -2.139490 2.120138 1.598156
H -2.613824 1.398853 2.027434

ENERGIES
SCF ENERGY: -952.282695568
SUM OF ELECTRONIC AND ZERO-POINT ENERGIES: -952.088853
SUM OF ELECTRONIC AND THERMAL ENERGIES: -952.072157
SUM OF ELECTRONIC AND THERMAL ENTHALPIES: -952.071213
SUM OF ELECTRONIC AND THERMAL FREE ENERGIES: -952.135557

==> AOX-I/WATER/HIS(2-)_C2_OH <==
28
DATASET
C -1.989875 -0.363585 -0.118294
C -2.722375 -1.557136 -0.196176
C -4.109495 -1.571338 -0.132038
C -4.848424 -0.376894 0.017388
C -4.075681 0.832876 0.092102
C -2.708052 0.849012 0.027305
O -4.791907 1.998331 0.237386
O -6.135061 -0.294190 0.093007
C -0.545793 -0.423477 -0.180268
C 0.315523 0.617505 -0.120656
C 1.749686 0.467788 -0.178947
C 2.610453 1.520945 -0.117467
C 4.035338 1.327319 -0.202638
C 4.469160 -0.041034 -0.272273
C 3.532008 -1.144039 -0.217520
O 2.176049 -0.814022 -0.328350
O 4.856112 2.273508 -0.214848
H -2.183128 -2.492925 -0.308049
H -4.654573 -2.507346 -0.193933
H -2.193229 1.801817 0.093616
H -0.131501 -1.423425 -0.281714
H -0.032219 1.639605 -0.020766
H 2.211808 2.521894 -0.016942
H -5.722891 1.717655 0.262707
H 5.523281 -0.282842 -0.311117
O 3.799062 -2.319172 -0.473855
O 3.652684 -0.893785 1.744211
H 4.452095 -1.407430 1.912696

ENERGIES
SCF ENERGY: -952.279516010
SUM OF ELECTRONIC AND ZERO-POINT ENERGIES: -952.085422
SUM OF ELECTRONIC AND THERMAL ENERGIES: -952.069047
SUM OF ELECTRONIC AND THERMAL ENTHALPIES: -952.068102
SUM OF ELECTRONIC AND THERMAL FREE ENERGIES: -952.131172

==> AOX-I/WATER/HIS(2-)_C3_OH <==
28
DATASET

C	-2.065196	-0.390342	-0.068127
C	-2.809299	-1.577724	-0.037239
C	-4.196995	-1.572518	0.043876
C	-4.922782	-0.363398	0.098251
C	-4.138501	0.839109	0.059046
C	-2.770833	0.836598	-0.020266
O	-4.843866	2.019496	0.108571
O	-6.209613	-0.258905	0.180992
C	-0.619630	-0.469028	-0.140236
C	0.249449	0.565476	-0.161148
C	1.682692	0.410383	-0.222137
C	2.565172	1.443401	-0.242440
C	3.985456	1.211787	-0.269051
C	4.396862	-0.164599	-0.125184
C	3.442704	-1.212891	-0.345073
O	2.106658	-0.887812	-0.275675
O	4.826084	2.143565	-0.349785
O	3.696206	-2.396633	-0.532237
H	-2.280297	-2.525246	-0.074966
H	-4.750519	-2.505355	0.069086
H	-2.247578	1.786849	-0.043489
H	-0.216332	-1.478023	-0.175477
H	-0.090737	1.594556	-0.124427
H	2.186672	2.457489	-0.237818
H	-5.776339	1.747393	0.165723
H	5.419581	-0.415342	-0.375447
O	4.599162	-0.327033	1.810389
H	5.563901	-0.341263	1.837138

ENERGIES
SCF ENERGY: -952.287963460
SUM OF ELECTRONIC AND ZERO-POINT ENERGIES: -952.093587
SUM OF ELECTRONIC AND THERMAL ENERGIES: -952.076979
SUM OF ELECTRONIC AND THERMAL ENTHALPIES: -952.076035
SUM OF ELECTRONIC AND THERMAL FREE ENERGIES: -952.139712

==> AOX-I/WATER/HIS(2-)_C5_OH <==
28
DATASET

C	-1.947618	0.469654	0.011495
C	-2.765296	1.627851	0.174418
C	-4.130282	1.556759	0.175450
C	-4.799566	0.301441	0.010854
C	-3.950077	-0.870782	-0.152948
C	-2.592766	-0.793534	-0.153428
O	-4.595117	-2.060405	-0.307649
O	-6.051426	0.150773	-0.000807
C	-0.545702	0.635988	0.017387
C	0.396520	-0.362579	-0.133868
C	1.784224	-0.140437	-0.136095
C	2.709325	-1.159458	-0.265945
C	4.125167	-0.843324	-0.493601
C	4.470875	0.512237	-0.371511
C	3.532412	1.510770	-0.065711
O	2.194984	1.152857	0.055505
O	4.935027	-1.769023	-0.773847
H	-2.271956	2.586232	0.299393
H	-4.742118	2.443011	0.299167
H	-2.016722	-1.702886	-0.279991
H	-0.187902	1.652761	0.150477
H	0.098022	-1.396123	-0.269099
H	-5.546288	-1.864071	-0.267220
H	5.499462	0.825386	-0.493992
O	3.747013	2.715256	0.109580
H	2.350937	-2.148909	-0.510849
O	3.125537	-1.872204	1.825622
H	2.245117	-1.600942	2.105895

ENERGIES
SCF ENERGY: -952.285553060
SUM OF ELECTRONIC AND ZERO-POINT ENERGIES: -952.091496
SUM OF ELECTRONIC AND THERMAL ENERGIES: -952.074794
SUM OF ELECTRONIC AND THERMAL ENTHALPIES: -952.073850
SUM OF ELECTRONIC AND THERMAL FREE ENERGIES: -952.137637

==> AOX-I/WATER/HIS(2-)_C6_OH <==
28
DATASET

C	-1.899733	0.432575	-0.022164
C	-2.665840	1.606782	-0.046350
C	-4.053729	1.575276	-0.085407
C	-4.757594	0.350843	-0.101644
C	-3.950115	-0.838187	-0.072088
C	-2.581978	-0.809052	-0.034234
O	-4.635451	-2.030470	-0.084748
O	-6.041768	0.221958	-0.140482
C	-0.457372	0.538456	0.010786
C	0.431784	-0.477745	0.005440
C	1.863826	-0.272689	0.027893
C	2.763685	-1.300232	-0.269161
C	4.178404	-1.020439	-0.469436
C	4.546484	0.334042	-0.376084
C	3.619398	1.356024	-0.127542
O	2.265568	1.011939	0.002236
O	4.979029	-1.968677	-0.708882
H	-2.152679	2.563598	-0.035847
H	-4.626148	2.496629	-0.105098
H	-2.039038	-1.748184	-0.014097
H	-0.070731	1.554496	0.032224
H	0.122533	-1.516586	-0.021282
H	-5.574156	-1.776059	-0.114722
H	5.581423	0.627552	-0.491861
O	3.830710	2.564248	-0.022154
H	2.395955	-2.316307	-0.329179
O	2.389844	-0.705887	1.991023
H	1.836067	-1.483949	2.143874

ENERGIES
SCF ENERGY: -952.273957111
SUM OF ELECTRONIC AND ZERO-POINT ENERGIES: -952.080126
SUM OF ELECTRONIC AND THERMAL ENERGIES: -952.063652
SUM OF ELECTRONIC AND THERMAL ENTHALPIES: -952.062708
SUM OF ELECTRONIC AND THERMAL FREE ENERGIES: -952.125787

==> AOX-I/WATER/HIS(2-)_C7_OH <==
28
DATASET

C	-1.752128	0.455443	-0.125852
C	-2.521308	1.645894	-0.316658
C	-3.884451	1.622900	-0.385638
C	-4.605025	0.389693	-0.261010
C	-3.806073	-0.813009	-0.060121
C	-2.447199	-0.782960	0.005953
O	-4.495892	-1.977059	0.064784
O	-5.859444	0.284196	-0.311006
C	-0.345439	0.564788	-0.067132
C	0.529342	-0.495328	0.078160
C	1.968344	-0.315575	-0.053078
C	2.819477	-1.300470	-0.403994
C	4.245766	-1.032377	-0.539615
C	4.646010	0.296192	-0.283717
C	3.740740	1.297480	0.082241
O	2.394285	0.950452	0.191856
O	5.041813	-1.958125	-0.870000
H	-1.988149	2.586143	-0.410658
H	-4.459117	2.530138	-0.533292

H	-1.910246	-1.710387	0.166838
H	0.066847	1.567733	-0.112930
H	-5.437965	-1.750570	-0.013255
H	5.688624	0.574685	-0.364513
O	3.973549	2.487910	0.330158
H	2.439158	-2.297758	-0.584524
H	0.194650	-1.515801	-0.047057
O	0.577992	-0.932039	2.269891
H	0.819443	-0.022897	2.476698

ENERGIES
SCF ENERGY: -952.290535504
SUM OF ELECTRONIC AND ZERO-POINT ENERGIES: -952.096251
SUM OF ELECTRONIC AND THERMAL ENERGIES: -952.079471
SUM OF ELECTRONIC AND THERMAL ENTHALPIES: -952.078527
SUM OF ELECTRONIC AND THERMAL FREE ENERGIES: -952.142836

==> AOX-I/WATER/HIS(2-)_C8_OH <==
28
DATASET

C	-1.728744	0.359970	-0.100588
C	-2.499549	1.505274	-0.420112
C	-3.867090	1.450679	-0.528504
C	-4.572189	0.230422	-0.302843
C	-3.766207	-0.924580	0.040370
C	-2.409247	-0.867743	0.140089
O	-4.448997	-2.089790	0.263906
O	-5.831264	0.094845	-0.383078
C	-0.319399	0.503828	0.003752
C	0.575026	-0.582032	-0.009162
C	1.983134	-0.421270	-0.084782
C	2.857622	-1.468556	-0.106725
C	4.293822	-1.240368	-0.180128
C	4.690467	0.110810	-0.227089
C	3.774399	1.173198	-0.205136
O	2.418712	0.868042	-0.128024
O	5.098086	-2.216482	-0.198297
H	-1.980428	2.443482	-0.589053
H	-4.445800	2.331162	-0.784058
H	-1.867651	-1.766831	0.411615
H	-5.388249	-1.876009	0.134415
H	5.740353	0.366902	-0.284380
O	4.016179	2.383234	-0.245283
H	2.473265	-2.479896	-0.067173
H	0.219161	-1.601913	0.063340
H	0.080242	1.478064	-0.246467
O	0.017951	1.056334	2.093039
H	0.931506	1.357783	2.028807

ENERGIES
SCF ENERGY: -952.279937423
SUM OF ELECTRONIC AND ZERO-POINT ENERGIES: -952.087079
SUM OF ELECTRONIC AND THERMAL ENERGIES: -952.070178
SUM OF ELECTRONIC AND THERMAL ENTHALPIES: -952.069234
SUM OF ELECTRONIC AND THERMAL FREE ENERGIES: -952.133667

==> AOX-I/WATER/HIS(2-)_C9_OH <==
28
DATASET

C	1.646512	-0.476700	0.234783
C	2.380243	-1.481688	-0.496174
C	3.688912	-1.309542	-0.849378
C	4.378697	-0.090475	-0.565221
C	3.599931	0.954192	0.078223
C	2.295388	0.793993	0.422002
O	4.253898	2.131197	0.308327
O	5.597537	0.138577	-0.848882
C	0.178067	-0.552961	0.132451
C	-0.652049	0.496480	0.088330
C	-2.100924	0.380146	-0.011604
C	-2.938340	1.436607	-0.057979
C	-4.378903	1.237651	-0.155147
C	-4.811994	-0.104857	-0.196756
C	-3.922454	-1.181982	-0.150712
O	-2.559628	-0.898194	-0.054317
O	-5.162559	2.229977	-0.197899
H	1.865520	-2.407507	-0.730622
H	4.234912	-2.089983	-1.368273
H	1.765525	1.609983	0.900288
H	-0.225449	-1.559785	0.077851
H	-0.281983	1.515615	0.115893
H	5.155674	2.006906	-0.032588
H	-5.867220	-0.333420	-0.269836
O	-4.179835	-2.392876	-0.184485
H	-2.535217	2.440632	-0.022670
O	1.683890	-1.265747	2.025208
H	2.643293	-1.331103	2.109422

ENERGIES
SCF ENERGY: -952.285415169
SUM OF ELECTRONIC AND ZERO-POINT ENERGIES: -952.090919
SUM OF ELECTRONIC AND THERMAL ENERGIES: -952.074284
SUM OF ELECTRONIC AND THERMAL ENTHALPIES: -952.073339
SUM OF ELECTRONIC AND THERMAL FREE ENERGIES: -952.137250

==> AOX-II/HIS- <==
27
DATASET

C	1.700869	0.416824	-0.000010
C	2.456353	1.592866	-0.000013
C	3.842677	1.554378	-0.000002
C	4.500741	0.337765	0.000009
C	3.757522	-0.850611	0.000008
C	2.379528	-0.811055	0.000000
O	4.490907	-2.001161	0.000014
O	5.860702	0.306544	0.000018
C	0.250980	0.518616	-0.000019
C	-0.626694	-0.497907	0.000030
C	-2.061355	-0.340569	0.000018
C	-2.930795	-1.375554	0.000093
C	-4.361329	-1.148303	0.000079
C	-4.761928	0.203774	-0.000014
C	-3.857030	1.262956	-0.000106
O	-2.493291	0.946255	-0.000075
O	-5.173137	-2.118740	0.000157
H	1.943939	2.550737	-0.000023
H	4.431259	2.466622	-0.000002
H	1.837710	-1.754003	-0.000002
H	-0.135645	1.536792	-0.000067
H	-0.291519	-1.532979	0.000087
H	3.909966	-2.772347	0.000013
H	6.158964	-0.614013	0.000023
H	-5.818380	0.453111	-0.000025
O	-4.088362	2.476831	-0.000191
H	-2.548086	-2.390839	0.000166

ENERGIES
SCF ENERGY: -876.781635977
SUM OF ELECTRONIC AND ZERO-POINT ENERGIES: -876.588505
SUM OF ELECTRONIC AND THERMAL ENERGIES: -876.573171
SUM OF ELECTRONIC AND THERMAL ENTHALPIES: -876.572227
SUM OF ELECTRONIC AND THERMAL FREE ENERGIES: -876.632355

==> AOX-II/HIS-/AIP <==
27
DATASET

C	1.681848	0.408286	0.000005
C	2.447213	1.604995	-0.000041

C 3.815240 1.570320 -0.000055
C 4.469047 0.335979 -0.000025
C 3.720713 -0.874696 0.000017
C 2.355362 -0.835477 0.000035
O 4.474492 -1.991720 0.000030
O 5.792970 0.304778 -0.000040
C 0.272990 0.532749 0.000018
C -0.629563 -0.501300 0.000063
C -2.030885 -0.313760 0.000069
C -2.906384 -1.366889 0.000107
C -4.337550 -1.162354 0.000064
C -4.729252 0.192096 -0.000007
C -3.821723 1.268780 0.000115
O -2.459386 0.968449 0.000038
O -5.140143 -2.132223 -0.000035
H 1.923566 2.555555 -0.000065
H 4.414191 2.474164 -0.000089
H 1.806832 -1.772485 0.000066
H -0.116021 1.548702 -0.000015
H -0.302808 -1.537201 0.000098
H 3.925313 -2.787732 0.000035
H 6.111893 -0.613315 -0.000020
H -5.783306 0.449819 -0.000182
O -4.082585 2.463859 -0.000261
H -2.504771 -2.375017 0.000125

ENERGIES
SCF ENERGY: -876.588153699
SUM OF ELECTRONIC AND ZERO-POINT ENERGIES: -876.394634
SUM OF ELECTRONIC AND THERMAL ENERGIES: -876.379607
SUM OF ELECTRONIC AND THERMAL ENTHALPIES: -876.378662
SUM OF ELECTRONIC AND THERMAL FREE ENERGIES: -876.438550

==> AOX-II/HIS-/CU2+/BIS <==
55
DATASET
C -5.174371 -0.604558 0.188013
C -5.143619 -1.997658 0.327042
C -3.943563 -2.687856 0.385198
C -2.756804 -1.984361 0.300278
C -2.774582 -0.606874 0.157846
C -3.959883 0.087454 0.106856
O -1.532747 0.002280 0.097699
O -1.503853 -2.549781 0.358964
C -6.403084 0.175472 0.130047
C -7.656725 -0.300398 0.144035
C -8.839430 0.527756 0.089935
C -10.097127 0.037216 0.084837
C -11.244415 0.922613 0.028304
C -10.945644 2.299301 -0.017965
C -9.642316 2.793974 -0.010333
O -8.598476 1.861826 0.043615
O -12.418938 0.454465 0.022704
H -6.069456 -2.559361 0.394878
H -3.917731 -3.767818 0.492056
H -3.942611 1.168165 -0.005593
H -6.260204 1.252999 0.068874
H -7.859942 -1.367884 0.194613
H -1.544356 0.849964 -0.373083
H -1.495710 -3.494366 0.147211
H -11.751145 3.025643 -0.060144
O -9.262408 3.968398 -0.045146
H -10.249112 -1.036353 0.123083
CU -0.012492 -1.225121 -0.154512
C 5.146700 -0.488004 -0.309997
C 5.115203 -1.884007 -0.206941
C 3.919249 -2.578793 -0.246567
C 2.743380 -1.872078 -0.391756
C 2.752365 -0.492561 -0.513447
C 3.939699 0.204515 -0.478063
O 1.506861 0.083650 -0.659949
O 1.494509 -2.465267 -0.430934
C 6.383582 0.280022 -0.230814
C 7.600955 -0.226779 0.014119
C 8.818533 0.541753 0.115436
C 10.021712-0.013341 0.374927
C 11.215269 0.802529 0.477203
C 11.018565 2.185542 0.292506
C 9.771359 2.746961 0.021226
O 8.674585 1.878585 -0.062188
O 12.338482 0.274140 0.717448
H 6.036094 -2.444400 -0.087692
H 3.887308 -3.659899 -0.154569
H 3.928664 1.288233 -0.558809
H 6.278381 1.354538 -0.370666
H 7.743622 -1.293894 0.169885
H 1.492754 1.017335 -0.404397
H 1.470965 -3.326101 0.015025
H 11.863410 2.864074 0.355537
O 9.485574 3.933957 -0.163174
H 10.092586-1.087436 0.510792

ENERGIES
SCF ENERGY: -1950.64981505
SUM OF ELECTRONIC AND ZERO-POINT ENERGIES: -1950.259103
SUM OF ELECTRONIC AND THERMAL ENERGIES: -1950.225547
SUM OF ELECTRONIC AND THERMAL ENTHALPIES: -1950.224602
SUM OF ELECTRONIC AND THERMAL FREE ENERGIES: -1950.329219

==> AOX-II/HIS-/CU2+/BIS/AEA <==
55
DATASET
C -5.427428 -0.825932 0.257502
C -5.510816 -2.221975 0.297959
C -4.366495 -2.994319 0.378566
C -3.108120 -2.404590 0.423490
C -3.017475 -1.014592 0.369742
C -4.156698 -0.241378 0.290075
O -1.764179 -0.444634 0.428500
O -1.956363 -3.115099 0.521845
C -6.584368 0.052731 0.191150
C -7.875016 -0.315733 0.190704
C -8.982072 0.610110 0.132517
C -10.279498 0.234070 0.145418
C -11.342123 1.217918 0.089436
C -10.923012 2.562698 0.023891
C -9.581545 2.939030 0.013187
O -8.625063 1.917520 0.064159
O -12.555360 0.860630 0.102544
H -6.477198 -2.715228 0.268209
H -4.431633 -4.078684 0.413898
H -4.055970 0.841482 0.256234
H -6.351814 1.115544 0.145631
H -8.165922 -1.362865 0.239970
H -1.815392 0.516693 0.333166
H -2.152533 -4.061321 0.518581
H -11.661941 3.356734 -0.017106
O -9.097000 4.074447 -0.036302
H -10.526455 -0.820996 0.201099
CU -0.096344 -1.242623 -0.250043
C 5.326894 -0.325941 -0.388885
C 5.152143 -1.697079 -0.174613
C 3.907244 -2.276946 -0.338834
C 2.821016 -1.508776 -0.723858
C 2.970761 -0.142614 -0.952028
C 4.217655 0.433303 -0.776037
O 1.873546 0.559709 -1.342850
O 1.573520 -2.061312 -0.917289
C 6.606544 0.350993 -0.232289
C 7.769855 -0.222071 0.112185

C 9.022167 0.482593 0.253291
C 10.186271 -0.120692 0.578141
C 11.413413 0.640251 0.701820
C 11.294487 2.024781 0.465335
C 10.087115 2.637357 0.134639
O 8.954364 1.819676 0.032261
O 12.500517 0.068033 1.002286
H 5.987813 -2.321558 0.123767
H 3.762357 -3.341092 -0.170869
H 4.327321 1.501097 -0.954087
H 6.591120 1.423128 -0.421495
H 7.835748 -1.290381 0.307104
H 2.099993 1.494331 -1.440423
H 1.596907 -3.017111 -0.766745
H 12.169365 2.662697 0.541790
O 9.863114 3.831870 -0.086381
H 10.200058 -1.192300 0.747323

ENERGIES
SCF ENERGY: -1950.83902318
SUM OF ELECTRONIC AND ZERO-POINT ENERGIES: -1950.448905
SUM OF ELECTRONIC AND THERMAL ENERGIES: -1950.414679
SUM OF ELECTRONIC AND THERMAL ENTHALPIES: -1950.413735
SUM OF ELECTRONIC AND THERMAL FREE ENERGIES: -1950.521756

==> AOX-II/HIS-/CU2+/BIS/AIP <==
55
DATASET
C -5.358162 -0.645842 0.124563
C -5.338377 -2.015783 0.411613
C -4.142648 -2.710111 0.473195
C -2.942015 -2.052817 0.250867
C -2.948517 -0.690684 -0.039860
C -4.138610 0.002687 -0.105366
O -1.724740 -0.102930 -0.237447
O -1.725463 -2.661947 0.290730
C -6.578452 0.144726 0.060636
C -7.821844 -0.282833 0.329282
C -9.004136 0.542838 0.258680
C -10.244407 0.100407 0.559900
C -11.392052 0.981771 0.479482
C -11.115737 2.300857 0.065673
C -9.832058 2.745437 -0.245618
O -8.784306 1.822863 -0.133824
O -12.549404 0.563749 0.771126
H -6.264560 -2.553782 0.586775
H -4.123933 -3.774264 0.691859
H -4.118018 1.065894 -0.333491
H -6.439811 1.185891 -0.226749
H -8.013240 -1.309775 0.632596
H -1.804228 0.791782 -0.595358
H -1.812962 -3.611855 0.444434
H -11.924029 3.020266 -0.019798
O -9.472524 3.867596 -0.616097
H -10.380259 -0.930234 0.870463
CU -0.024252 -1.282232 -0.377170
C 5.339260 -0.376731 -0.264225
C 5.277695 -1.799683 -0.053352
C 4.097086 -2.458036 -0.155142
C 2.934399 -1.738893 -0.491177
C 2.962942 -0.317506 -0.686798
C 4.151168 0.335638 -0.578931
O 1.779936 0.237215 -0.958950
O 1.768117 -2.312872 -0.655349
C 6.523056 0.343858 -0.151584
C 7.771628 -0.208588 0.138431
C 8.931415 0.557553 0.248325
C 10.146859 -0.047770 0.502946
C 11.376100 0.702691 0.612255
C 11.183828 2.091173 0.452829
C 9.926270 2.709095 0.197681
O 8.809918 1.890278 0.092486
O 12.475417 0.147719 0.828875
H 6.176041 -2.354618 0.187947
H 4.017961 -3.529591 -0.004351
H 4.194125 1.410721 -0.725594
H 6.466452 1.417966 -0.309291
H 7.899391 -1.276253 0.285361
H 1.842785 1.198440 -1.067998
H 1.778230 -3.275848 -0.501538
H 12.031588 2.764948 0.523267
O 9.726196 3.897222 0.057509
H 10.164463 -1.126829 0.621098

ENERGIES
SCF ENERGY: -1950.42010968
SUM OF ELECTRONIC AND ZERO-POINT ENERGIES: -1950.030079
SUM OF ELECTRONIC AND THERMAL ENERGIES: -1949.995744
SUM OF ELECTRONIC AND THERMAL ENTHALPIES: -1949.994799
SUM OF ELECTRONIC AND THERMAL FREE ENERGIES: -1950.100961

==> AOX-II/HIS-/CU2+/BIS/HAT/C3B <==
54
DATASET
C 5.146865 0.137418 -0.435148
C 4.774432 0.660486 -1.744643
C 3.503348 0.626178 -2.215440
C 2.501567 0.068683 -1.403940
C 2.832347 -0.490256 -0.055634
C 4.181507 -0.413334 0.364664
O 1.932584 -0.999177 0.638891
O 1.267008 -0.003192 -1.736305
C 6.505229 0.184161 0.007952
C 7.545603 0.705270 -0.686806
C 8.896051 0.718651 -0.211358
C 9.911667 1.255810 -0.928706
C 11.272073 1.254219 -0.420045
C 11.428491 0.655796 0.843839
C 10.369802 0.100022 1.573331
O 9.093327 0.155130 1.002915
O 12.210623 1.762367 -1.090644
H 5.541668 1.098039 -2.374613
H 3.237255 1.022451 -3.189516
H 4.426607 -0.815032 1.342356
H 6.693627 -0.245550 0.989048
H 7.415755 1.153316 -1.667514
H 1.085292 0.368789 -2.620382
H 12.412442 0.608080 1.299069
O 10.408260 -0.443027 2.675061
H 9.700010 1.694514 -1.898132
CU 0.057968 -1.220651 0.435363
C -5.249304 -0.710346 0.092372
C -5.124624 -1.969800 -0.505043
C -3.893076 -2.597850 -0.590073
C -2.766794 -1.977020 -0.078601
C -2.874335 -0.725401 0.518707
C -4.098726 -0.098123 0.605356
O -1.706605 -0.194398 1.000164
O -1.508145 -2.513123 -0.120337
C -6.513379 0.002254 0.211273
C -7.710952 -0.415588 -0.226196
C -8.938160 0.332511 -0.085209
C -10.133105 -0.096218 -0.546267
C -11.330038 0.703590 -0.376974
C -11.149134 1.931235 0.292009
C -9.911217 2.362200 0.765774
O -8.810358 1.522849 0.553126
O -12.445296 0.296435 -0.812408
H -5.995816 -2.471911 -0.913166
H -3.792274 -3.573788 -1.056402

H -4.159737 0.881503 1.073517
H -6.452899 0.969332 0.708022
H -7.826858 -1.372747 -0.729915
H -1.795978 0.739439 1.232656
H -1.480895 -3.344289 -0.613771
H -11.998896 2.585540 0.459190
O -9.635454 3.407165 1.363844
H -10.194744 -1.054343 -1.051589

ENERGIES
SCF ENERGY: -1950.00937603
SUM OF ELECTRONIC AND ZERO-POINT ENERGIES: -1949.631508
SUM OF ELECTRONIC AND THERMAL ENERGIES: -1949.598464
SUM OF ELECTRONIC AND THERMAL ENTHALPIES: -1949.597520
SUM OF ELECTRONIC AND THERMAL FREE ENERGIES: -1949.700595

==> AOX-II/HIS-/CU2+/BIS/HAT/C4B <==
54
DATASET
C -5.368311 -0.190156 0.228432
C -5.134392 -1.182565 1.261541
C -3.891767 -1.599164 1.547164
C -2.747297 -1.072569 0.833182
C -3.003032 -0.071450 -0.228243
C -4.276602 0.338918 -0.490452
O -1.924050 0.355109 -0.848263
O -1.602917 -1.448780 1.108089
C -6.655660 0.271075 -0.090128
C -7.810515 -0.152170 0.520219
C -9.099908 0.327466 0.184484
C -10.214438 -0.139257 0.811336
C -11.538281 0.346484 0.462124
C -11.543298 1.321980 -0.549627
C -10.383581 1.800479 -1.187153
O -9.157132 1.264712 -0.787270
O -12.560859 -0.098367 1.043272
H -5.975126 -1.592137 1.808421
H -3.689377 -2.336979 2.315511
H -4.454404 1.078457 -1.265902
H -6.727988 1.016744 -0.877579
H -7.796306 -0.897235 1.310717
H -2.136373 1.013150 -1.530488
H -12.484344 1.750332 -0.878957
O -10.310484 2.646169 -2.068994
H -10.103552 -0.891477 1.585617
CU 0.121395 -1.044505 0.400336
C 5.350785 -0.394135 -0.402592
C 5.150981 -1.455359 -1.292725
C 3.878910 -1.938862 -1.547872
C 2.785376 -1.370039 -0.917197
C 2.967937 -0.313288 -0.031316
C 4.232901 0.171743 0.223744
O 1.827698 0.188528 0.542786
O 1.493397 -1.778072 -1.102884
C 6.663453 0.152149 -0.089224
C 7.839364 -0.269407 -0.579032
C 9.120904 0.300264 -0.235577
C 10.294364 -0.142175 -0.737266
C 11.549970 0.470492 -0.352201
C 11.448501 1.537888 0.563101
C 10.231874 1.985531 1.074959
O 9.070374 1.331307 0.644759
O 12.645272 0.050065 -0.824508
H 5.995677 -1.915747 -1.794962
H 3.720274 -2.765166 -2.235060
H 4.354278 0.995259 0.923669
H 6.662561 0.981480 0.616453
H 7.893603 -1.094893 -1.285571
H 2.026919 0.784295 1.277705
H 1.444730 -2.615940 -1.582990
H 12.345768 2.047644 0.899576
O 10.021242 2.901776 1.876109
H 10.293457 -0.969945 -1.438767

ENERGIES
SCF ENERGY: -1950.02313726
SUM OF ELECTRONIC AND ZERO-POINT ENERGIES: -1949.644807
SUM OF ELECTRONIC AND THERMAL ENERGIES: -1949.610791
SUM OF ELECTRONIC AND THERMAL ENTHALPIES: -1949.609847
SUM OF ELECTRONIC AND THERMAL FREE ENERGIES: -1949.717743

==> AOX-II/HIS-/CU2+/BIS/RAF/C1B <==
57
DATASET
C -5.602079 -1.991887 1.301138
C -5.791404 -2.867702 0.112819
C -4.772715 -3.279438 -0.651471
C -3.433673 -2.935236 -0.299043
C -3.154013 -2.158413 0.872416
C -4.168394 -1.755258 1.651647
O -1.835973 -1.923024 1.128027
O -2.420753 -3.321105 -0.991338
C -6.148744 -0.601047 1.050357
C -6.606448 -0.125781 -0.109260
C -7.113802 1.219542 -0.285761
C -7.582221 1.684292 -1.459926
C -8.080463 3.044863 -1.576498
C -8.036631 3.804721 -0.392063
C -7.550443 3.307135 0.817666
O -7.085119 1.985195 0.830437
O -8.515175 3.476170 -2.680496
H -6.815624 -3.146005 -0.119084
H -4.918036 -3.898717 -1.531085
H -3.977965 -1.188002 2.560776
H -6.124703 0.025230 1.941646
H -6.628197 -0.738175 -1.008043
H -1.725091 -1.457486 1.971897
H -2.670484 -3.847140 -1.773975
H -8.392511 4.830077 -0.395950
O -7.466852 3.877647 1.907550
H -7.587067 1.033310 -2.327733
CU -0.555465 -1.234148 -0.235969
C 4.714152 0.195322 -0.512388
C 4.417836 -0.472253 -1.705695
C 3.104166 -0.726369 -2.057383
C 2.067157 -0.318284 -1.235253
C 2.335629 0.363076 -0.049388
C 3.652891 0.606578 0.300720
O 1.278011 0.753101 0.711869
O 0.747045 -0.559012 -1.557025
C 6.071531 0.476093 -0.065882
C 7.202628 0.169568 -0.719050
C 8.532742 0.451602 -0.233163
C 9.657526 0.142006 -0.913709
C 10.966572 0.445108 -0.370818
C 10.968795 1.071428 0.891991
C 9.801361 1.386694 1.585077
O 8.581884 1.057120 0.980158
O 12.015588 0.152702 -1.013826
H 5.212274 -0.806013 -2.365167
H 2.866534 -1.252590 -2.978228
H 3.854806 1.130577 1.232901
H 6.147652 0.980252 0.896261
H 7.178595 -0.329220 -1.685355
H 1.593642 1.170914 1.523933
H 0.685804 -1.026653 -2.402385
H 11.911128 1.328624 1.365372
O 9.682448 1.930813 2.687590
H 9.576064 -0.342208 -1.881222
O -6.294871 -2.613595 2.371818

H -6.279949 -2.022749 3.137942

ENERGIES
SCF ENERGY: -2026.43531094
SUM OF ELECTRONIC AND ZERO-POINT ENERGIES: -2026.029098
SUM OF ELECTRONIC AND THERMAL ENERGIES: -2025.993558
SUM OF ELECTRONIC AND THERMAL ENTHALPIES: -2025.992613
SUM OF ELECTRONIC AND THERMAL FREE ENERGIES: -2026.102316

==> AOX-II/HIS-/CU2+/BIS/RAF/C1P <==
57
DATASET
C -5.306251 -0.933112 -0.044114
C -5.209625 -1.158320 1.366373
C -3.992314 -1.204803 1.969340
C -2.815132 -1.042120 1.214373
C -2.888321 -0.846239 -0.191011
C -4.097984 -0.786466 -0.800238
O -1.703396 -0.688763 -0.850086
O -1.620179 -1.053275 1.749821
C -6.490703 -0.802043 -0.718135
C -7.889655 -0.846896 -0.236309
C -8.349467 0.595704 -0.174208
C -9.252506 1.140941 -0.996588
C -9.620745 2.544496 -0.846554
C -8.971347 3.228343 0.198709
C -8.032973 2.632877 1.042502
O -7.734809 1.278908 0.812137
O -10.468274 3.067243 -1.619031
H -6.114839 -1.275232 1.946453
H -3.894993 -1.356526 3.039680
H -4.157589 -0.617408 -1.872109
H -6.427471 -0.560769 -1.778792
H -8.481339 -1.327077 -1.030523
H -1.851878 -0.499399 -1.788695
H -1.652618 -1.183316 2.712496
H -9.195482 4.275562 0.375076
O -7.410029 3.130126 1.981346
H -9.716073 0.538802 -1.770357
CU -0.082258 -1.790121 -0.556359
C 5.248447 -0.740373 -0.315299
C 4.953256 -1.664523 0.692962
C 3.746261 -2.340517 0.689944
C 2.820519 -2.112723 -0.313808
C 3.095445 -1.208441 -1.336774
C 4.303759 -0.532376 -1.325924
O 2.154227 -1.039194 -2.303049
O 1.614388 -2.782968 -0.351547
C 6.492208 0.015431 -0.367823
C 7.462447 0.011887 0.558825
C 8.686019 0.773311 0.469542
C 9.638896 0.769233 1.426728
C 10.846353 1.557456 1.282210
C 10.942301 2.306987 0.092234
C 9.954519 2.299783 -0.891010
O 8.820220 1.510362 -0.661350
O 11.734121 1.549862 2.182825
H 5.665507 -1.863799 1.486787
H 3.506445 -3.057926 1.470467
H 4.511640 0.174504 -2.126660
H 6.618716 0.640301 -1.250649
H 7.375638 -0.583901 1.464858
H 2.439748 -0.350766 -2.917875
H 1.561419 -3.412008 0.382430
H 11.818835 2.920682 -0.090058
O 9.932464 2.903952 -1.968154
H 9.494715 0.165999 2.316960
O -8.040216 -1.513409 0.989508
H -8.983464 -1.561932 1.189888

ENERGIES
SCF ENERGY: -2026.46206316
SUM OF ELECTRONIC AND ZERO-POINT ENERGIES: -2026.054149
SUM OF ELECTRONIC AND THERMAL ENERGIES: -2026.018868
SUM OF ELECTRONIC AND THERMAL ENTHALPIES: -2026.017923
SUM OF ELECTRONIC AND THERMAL FREE ENERGIES: -2026.128168

==> AOX-II/HIS-/CU2+/BIS/RAF/C2 <==
57
DATASET
C 5.015382 -0.852962 -0.380730
C 4.960003 -2.172541 -0.845154
C 3.747787 -2.819688 -0.978321
C 2.556412 -2.176571 -0.656839
C 2.604803 -0.863123 -0.186852
C 3.813302 -0.214178 -0.052570
O 1.413064 -0.240099 0.109416
O 1.342616 -2.761345 -0.780538
C 6.251414 -0.112249 -0.228787
C 7.489989 -0.563972 -0.500068
C 8.680193 0.221811 -0.340223
C 9.919278 -0.257992 -0.647503
C 11.111230 0.497308 -0.431066
C 10.886094 1.931366 -0.080278
C 9.520254 2.359275 0.193295
O 8.469512 1.460328 0.166912
O 12.254293 0.043905 -0.495351
H 5.869553 -2.701817 -1.109740
H 3.699863 -3.842723 -1.341351
H 3.823547 0.810676 0.311972
H 6.141127 0.907880 0.135678
H 7.661666 -1.571437 -0.869220
H 1.562998 0.642518 0.476270
H 1.440418 -3.675457 -1.080626
H 11.728230 2.485782 0.328484
O 10.078014 2.670247 -1.043179
H 9.999128 -1.280550 -0.998951
CU -0.231710 -1.119222 0.744263
C -5.644937 -0.289589 0.535734
C -5.433338 -1.665367 0.395550
C -4.194492 -2.213244 0.674000
C -3.150752 -1.407827 1.097729
C -3.337506 -0.035841 1.252711
C -4.578869 0.507225 0.965829
O -2.282216 0.705530 1.683849
O -1.910017 -1.931312 1.398023
C -6.920784 0.354685 0.257374
C -8.042881 -0.254856 -0.154310
C -9.292830 0.417341 -0.419394
C -10.412614 -0.220027 -0.824609
C -11.639482 0.510108 -1.073191
C -11.568884 1.902587 -0.865111
C -10.406816 2.549914 -0.449094
O -9.270444 1.760663 -0.230997
O -12.684937 -0.093413 -1.450414
H -6.235618 -2.317073 0.065337
H -4.019960 -3.280485 0.563953
H -4.716845 1.579884 1.086983
H -6.938738 1.433033 0.406877
H -8.072580 -1.330342 -0.314828
H -2.534967 1.637242 1.726316
H -1.907417 -2.890120 1.262864
H -12.446954 2.518448 -1.032194
O -10.227024 3.753910 -0.238998
H -10.390480 -1.295653 -0.965064
O 9.141447 3.362716 1.017102
H 9.870419 3.989045 1.128271

ENERGIES
SCF ENERGY: -2026.46214964

SUM OF ELECTRONIC AND ZERO-POINT ENERGIES: -2026.054575
SUM OF ELECTRONIC AND THERMAL ENERGIES: -2026.019448
SUM OF ELECTRONIC AND THERMAL ENTHALPIES: -2026.018504
SUM OF ELECTRONIC AND THERMAL FREE ENERGIES: -2026.127717

==> AOX-II/HIS-/CU2+/BIS/RAF/C2B <==
57
DATASET

C	-5.120989	-0.631311	-0.041846
C	-5.083167	-1.793862	0.690841
C	-3.915750	-2.557193	0.779252
C	-2.711608	-2.201842	0.194394
C	-2.659400	-1.028915	-0.537827
C	-3.927577	-0.338821	-0.891968
O	-1.545652	-0.554246	-1.007979
O	-1.573266	-2.927467	0.375540
C	-6.241921	0.249757	-0.150354
C	-7.405068	0.081851	0.515380
C	-8.527871	0.971959	0.427863
C	-9.674733	0.765285	1.111695
C	-10.791427	1.687862	0.994795
C	-10.572865	2.780139	0.135643
C	-9.379848	2.982198	-0.564944
O	-8.361150	2.037094	-0.392024
O	-11.859858	1.486031	1.634836
H	-5.951258	-2.128773	1.246770
H	-3.930294	-3.464287	1.379658
H	-4.195899	-0.902287	-1.821055
H	-6.126489	1.113587	-0.798590
H	-7.555711	-0.762922	1.183141
H	-1.717390	0.321900	-1.412087
H	-1.704000	-3.637240	1.020371
H	-11.355732	3.518243	-0.006626
O	-9.090828	3.898721	-1.334466
H	-9.758757	-0.102778	1.756815
CU	0.334408	-2.537937	-0.108580
C	5.360962	-0.760987	-0.145656
C	5.624061	-2.121068	0.052008
C	4.606068	-3.056741	-0.014476
C	3.308772	-2.651282	-0.281307
C	3.031602	-1.302339	-0.492075
C	4.045475	-0.368854	-0.423210
O	1.725041	-0.991431	-0.752981
O	2.239401	-3.503937	-0.348134
C	6.386063	0.269971	-0.067661
C	7.687983	0.074995	0.191854
C	8.677437	1.123327	0.269113
C	9.985688	0.897862	0.518787
C	10.933774	1.991874	0.580896
C	10.392307	3.276115	0.370168
C	9.039949	3.497184	0.116121
O	8.199300	2.377472	0.070312
O	12.158278	1.773052	0.810127
H	6.633056	-2.458887	0.265532
H	4.804046	-4.112662	0.147616
H	3.808980	0.681448	-0.578583
H	6.035209	1.287021	-0.235602
H	8.083157	-0.923690	0.364384
H	1.569596	-0.038456	-0.704677
H	2.454593	-4.378092	0.004704
H	11.039396	4.146986	0.403250
O	8.456870	4.568378	-0.079777
H	10.329537	-0.119671	0.672357
O	-3.659759	0.998516	-1.192183
H	-4.074394	1.240304	-2.029636

ENERGIES
SCF ENERGY: -2026.44959347
SUM OF ELECTRONIC AND ZERO-POINT ENERGIES: -2026.043488
SUM OF ELECTRONIC AND THERMAL ENERGIES: -2026.007882
SUM OF ELECTRONIC AND THERMAL ENTHALPIES: -2026.006938
SUM OF ELECTRONIC AND THERMAL FREE ENERGIES: -2026.116260

==> AOX-II/HIS-/CU2+/BIS/RAF/C2P <==
57
DATASET

C	-5.246306	1.230440	-0.678761
C	-5.072874	2.080589	0.408829
C	-3.810918	2.260044	0.954624
C	-2.713158	1.596246	0.429212
C	-2.898346	0.734887	-0.657543
C	-4.148285	0.562215	-1.213925
O	-1.790086	0.088573	-1.156051
O	-1.457018	1.733439	0.914034
C	-7.193913	2.343349	-1.494498
C	-6.623264	1.010005	-1.289279
C	-7.490067	0.035013	-0.551160
C	-8.038197	-1.047522	-1.114469
C	-8.844332	-1.960520	-0.313510
C	-8.986209	-1.608845	1.042409
C	-8.406235	-0.472401	1.604145
O	-7.642200	0.343976	0.751734
O	-9.360186	-2.984318	-0.838360
H	-5.919924	2.601276	0.846419
H	-3.663258	2.923792	1.801773
H	-4.266082	-0.106618	-2.063267
H	-6.669046	3.054386	-2.138370
H	-6.468250	0.631565	-2.312608
H	-2.032338	-0.480629	-1.900777
H	-1.456844	2.330090	1.675173
H	-9.572247	-2.240652	1.702324
O	-8.462864	-0.063970	2.765334
H	-7.881497	-1.249852	-2.168445
CU	-0.419291	-0.755127	-0.005642
C	4.980888	-0.733947	0.271689
C	4.494475	-0.600512	1.576572
C	3.170068	-0.879377	1.861910
C	2.312560	-1.299711	0.859835
C	2.773906	-1.449422	-0.446405
C	4.099023	-1.160106	-0.727341
O	1.887021	-1.876906	-1.384484
O	0.986343	-1.592913	1.107884
C	6.360883	-0.449616	-0.097711
C	7.335619	-0.054999	0.735464
C	8.695957	0.224766	0.341549
C	9.654774	0.620162	1.206859
C	11.005109	0.888773	0.754847
C	11.228244	0.710534	-0.625508
C	10.231416	0.300290	-1.509200
O	8.956057	0.063158	-0.980407
O	11.898488	1.256709	1.570928
H	5.146155	-0.271238	2.379093
H	2.783698	-0.772099	2.871955
H	4.452311	-1.274603	-1.750148
H	6.596487	-0.576343	-1.153221
H	7.145904	0.080091	1.798132
H	2.314179	-1.907958	-2.250492
H	0.792553	-1.507230	2.053004
H	12.214506	0.892056	-1.040840
O	10.312065	0.109433	-2.726887
H	9.406948	0.737423	2.256639
O	-8.260449	2.690641	-0.933256
H	-8.526072	3.610640	-1.138285

ENERGIES
SCF ENERGY: -2026.46769898
SUM OF ELECTRONIC AND ZERO-POINT ENERGIES: -2026.059993
SUM OF ELECTRONIC AND THERMAL ENERGIES: -2026.024663
SUM OF ELECTRONIC AND THERMAL ENTHALPIES: -2026.023719
SUM OF ELECTRONIC AND THERMAL FREE ENERGIES: -

2026.134626

==> AOX-II/HIS-/CU2+/BIS/RAF/C3 <==
57
DATASET
C -5.046460 -1.047043 0.252705
C -5.103422 -2.446028 0.281389
C -3.946093 -3.192781 0.376833
C -2.701966 -2.573099 0.447801
C -2.636602 -1.179273 0.401735
C -3.789732 -0.431229 0.309414
O -1.395530 -0.588692 0.480824
O -1.541798 -3.257102 0.563609
C -6.217310 -0.200143 0.179536
C -7.501225 -0.607557 0.139940
C -8.615241 0.286749 0.072228
C -9.909478 -0.116501 0.051613
C -10.987889 0.821614 -0.023822
C -10.564192 2.221205 -0.433202
C -9.204300 2.600285 0.088809
O -8.262719 1.617699 0.082555
O -12.167829 0.552341 0.147490
H -6.058159 -2.959350 0.231634
H -3.984548 -4.278448 0.404786
H -3.714547 0.653675 0.284356
H -6.014792 0.869260 0.162027
H -7.768273 -1.660685 0.156182
H -1.460801 0.370963 0.376561
H -1.716220 -4.207959 0.558772
H -10.408235 2.135682 -1.527983
O -8.886969 3.702703 0.424922
H -10.129433 -1.174779 0.134430
CU 0.292201 -1.360855 -0.195358
C 5.685073 -0.293721 -0.360285
C 5.579659 -1.686008 -0.273294
C 4.353463 -2.306657 -0.427948
C 3.215138 -1.557361 -0.674418
C 3.294528 -0.169248 -0.769858
C 4.524443 0.446229 -0.609961
O 2.145418 0.514858 -1.019529
O 1.982559 -2.145533 -0.853303
C 6.944688 0.421738 -0.209184
C 8.138603 -0.129003 0.058224
C 9.375131 0.604328 0.189867
C 10.563878 0.017674 0.449041
C 11.776310 0.802866 0.562686
C 11.616139 2.192397 0.389149
C 10.384255 2.787076 0.121528
O 9.266887 1.947313 0.028585
O 12.886318 0.245270 0.800372
H 6.457030 -2.296041 -0.085750
H 4.265006 -3.387754 -0.360540
H 4.577045 1.530446 -0.686336
H 6.883956 1.502021 -0.331391
H 8.244591 -1.203455 0.192550
H 2.323258 1.464790 -1.016663
H 2.036510 -3.104519 -0.735430
H 12.478196 2.848342 0.458476
O 10.127755 3.982872 -0.052424
H 10.608978 -1.059460 0.571132
O -11.543634 3.157559 -0.115882
H -11.341436 3.988702 -0.562334

ENERGIES
SCF ENERGY: -2026.50241972
SUM OF ELECTRONIC AND ZERO-POINT ENERGIES: -2026.094938
SUM OF ELECTRONIC AND THERMAL ENERGIES: -2026.059383
SUM OF ELECTRONIC AND THERMAL ENTHALPIES: -2026.058439
SUM OF ELECTRONIC AND THERMAL FREE ENERGIES: -2026.167578

==> AOX-II/HIS-/CU2+/BIS/RAF/C3B <==
57
DATASET
C 5.217139 -0.755111 -0.002055
C 5.233966 -1.870676 -0.925787
C 4.224691 -2.779673 -1.046803
C 3.100561 -2.650238 -0.237102
C 2.939384 -1.476670 0.718874
C 4.137368 -0.594254 0.788179
O 1.849649 -0.754810 0.145357
O 2.109439 -3.461781 -0.217740
C 6.336142 0.169222 0.087482
C 7.469448 0.105642 -0.624923
C 8.560209 1.045410 -0.499589
C 9.706932 0.944393 -1.201799
C 10.776041 1.911727 -1.027372
C 10.513193 2.938379 -0.100123
C 9.319718 3.032493 0.616649
O 8.350226 2.047038 0.388159
O 11.853143 1.803544 -1.678264
H 6.105390 -2.006216 -1.559739
H 4.285784 -3.613408 -1.738132
H 4.065918 0.221452 1.504050
H 6.208121 0.971416 0.810705
H 7.644939 -0.678556 -1.356906
H 1.853938 0.161897 0.467262
H 2.201823 -4.206049 -0.843788
H 11.261629 3.702349 0.084762
O 8.986196 3.880904 1.447526
H 9.831847 0.125296 -1.902208
CU 0.016543 -1.516089 0.333721
C -5.320810 -0.452389 0.138600
C -5.281780 -1.811724 -0.191515
C -4.092376 -2.517227 -0.129872
C -2.927383 -1.878749 0.258568
C -2.943016 -0.527904 0.595640
C -4.132876 0.172926 0.535121
O -1.752348 0.021688 0.966425
O -1.719869 -2.529415 0.341789
C -6.533773 0.351626 0.085913
C -7.748000 -0.062108 -0.307260
C -8.923229 0.775707 -0.352302
C -10.134886 0.343386 -0.763645
C -11.276383 1.236134 -0.792504
C -11.025288 2.555711 -0.364602
C -9.770445 2.990327 0.058793
O -8.727047 2.056075 0.050810
O -12.407003 0.825340 -1.182535
H -6.183707 -2.330432 -0.500007
H -4.052665 -3.572426 -0.386095
H -4.132983 1.229044 0.795799
H -6.414421 1.388816 0.395240
H -7.918629 -1.086964 -0.629666
H -1.833792 0.977999 1.081633
H -1.763553 -3.406027 -0.063610
H -11.830630 3.283415 -0.357880
O -9.433640 4.112197 0.450735
H -10.252055 -0.688102 -1.079067
O 2.648951 -1.906768 2.002100
H 1.834437 -2.437191 1.985437

ENERGIES
SCF ENERGY: -2026.44374772
SUM OF ELECTRONIC AND ZERO-POINT ENERGIES: -2026.037235
SUM OF ELECTRONIC AND THERMAL ENERGIES: -2026.001727
SUM OF ELECTRONIC AND THERMAL ENTHALPIES: -2026.000782
SUM OF ELECTRONIC AND THERMAL FREE ENERGIES: -2026.110861

==> AOX-II/HIS-/CU2+/BIS/RAF/C4 <==
57

DATASET
C -4.923674 -0.243255 0.617563
C -4.678942 -1.477572 1.228604
C -3.383600 -1.871821 1.511529
C -2.317709 -1.045270 1.199791
C -2.534185 0.191886 0.595682
C -3.833120 0.576281 0.308330
O -1.448276 0.961124 0.320023
O -1.014602 -1.400608 1.483081
C -6.258533 0.235318 0.286377
C -7.410360 -0.413672 0.512511
C -8.716063 0.084405 0.163600
C -9.861778 -0.570710 0.386192
C -11.126348 -0.015089 -0.092107
C -11.102268 1.342865 -0.652292
C -9.803548 2.060944 -0.696626
O -8.689596 1.338990 -0.435849
O -11.197291 0.194666 -1.497866
H -5.498085 -2.141921 1.483150
H -3.182895 -2.830522 1.982178
H -3.997431 1.542365 -0.164905
H -6.295144 1.213208 -0.190272
H -7.426268 -1.393965 0.983150
H -1.725593 1.776422 -0.118525
H -0.992749 -2.233234 1.976661
H -11.982105 1.980793 -0.617085
O -9.690087 3.231247 -0.946834
H -9.849816 -1.532267 0.885049
CU 0.355048 -1.369177 0.076435
C 5.634660 -0.207429 -0.547240
C 5.389409 -1.429437 -1.182899
C 4.092333 -1.835212 -1.440520
C 3.023536 -1.033213 -1.077817
C 3.240209 0.192842 -0.452026
C 4.541216 0.588651 -0.190319
O 2.152457 0.941978 -0.130263
O 1.718352 -1.401517 -1.334918
C 6.971822 0.280529 -0.238889
C 8.126400 -0.351757 -0.498156
C 9.436499 0.164194 -0.178802
C 10.583851 -0.493656 -0.452874
C 11.871689 0.074799 -0.109066
C 11.828939 1.336534 0.517888
C 10.638554 2.005759 0.797121
O 9.442226 1.378569 0.426588
O 12.942017 -0.547138 -0.368048
H 6.210456 -2.074358 -1.478131
H 3.891835 -2.784142 -1.930911
H 4.704998 1.545798 0.300926
H 7.009424 1.253132 0.249290
H 8.139270 -1.325385 -0.983036
H 2.432570 1.752781 0.314914
H 1.693517 -2.233270 -1.829626
H 12.752920 1.827255 0.807174
O 10.481065 3.103753 1.340615
H 10.537254 -1.462928 -0.938140
O -12.229423 -0.547078 0.511378
H -13.027196 -0.087025 0.214925

ENERGIES
SCF ENERGY: -2026.46310303
SUM OF ELECTRONIC AND ZERO-POINT ENERGIES: -2026.055663
SUM OF ELECTRONIC AND THERMAL ENERGIES: -2026.020644
SUM OF ELECTRONIC AND THERMAL ENTHALPIES: -2026.019700
SUM OF ELECTRONIC AND THERMAL FREE ENERGIES: -2026.127618

==> AOX-II/HIS-/CU2+/BIS/RAF/C4B <==
57
DATASET
C 5.288218 -0.247348 0.382505
C 5.102838 -1.631979 -0.001945
C 3.926049 -2.236118 0.160319
C 2.733204 -1.551490 0.743283
C 3.005394 -0.119078 1.144139
C 4.221969 0.466693 0.950499
O 1.965353 0.465189 1.673553
O 1.718919 -1.475430 -0.258636
C 6.522183 0.428820 0.216801
C 7.634993 -0.116453 -0.344460
C 8.872274 0.579843 -0.498501
C 9.952545 0.004143 -1.076807
C 11.205211 0.731302 -1.217339
C 11.182955 2.039746 -0.705577
C 10.056704 2.622347 -0.107482
O 8.895022 1.846883 -0.025376
O 12.203392 0.186824 -1.759436
H 5.932619 -2.186605 -0.422394
H 3.766672 -3.275425 -0.120687
H 4.363540 1.501666 1.248142
H 6.560181 1.457877 0.563051
H 7.645266 -1.136065 -0.718798
H 2.149593 1.390413 1.912167
H 1.697622 -2.300349 -0.768847
H 12.075337 2.654717 -0.762611
O 9.948124 3.749727 0.366823
H 9.877976 -1.016343 -1.437556
CU -0.127352 -0.986252 0.259575
C -5.473869 -0.706973 -0.116075
C -5.442016 -2.008258 -0.629726
C -4.262444 -2.729359 -0.654540
C -3.083895 -2.177999 -0.168412
C -3.107591 -0.885138 0.350011
C -4.282050 -0.162086 0.375741
O -1.925327 -0.376274 0.841224
O -1.895789 -2.835761 -0.170004
C -6.678590 0.107876 -0.067235
C -7.891846 -0.237461 -0.525329
C -9.065437 0.600158 -0.455385
C -10.277718 0.225958 -0.919276
C -11.421939 1.109147 -0.818326
C -11.172045 2.358405 -0.214621
C -9.916544 2.734754 0.259166
O -8.870436 1.813737 0.119494
O -12.554440 0.750587 -1.252299
H -6.345461 -2.470172 -1.014828
H -4.235743 -3.740019 -1.053323
H -4.271750 0.846628 0.783038
H -6.556053 1.089623 0.387832
H -8.064714 -1.204281 -0.992966
H -2.016561 0.556723 1.079103
H -1.988744 -3.683615 -0.624805
H -11.979398 3.075282 -0.102898
O -9.581660 3.793309 0.800732
H -10.393869 -0.752924 -1.372535
O 2.275235 -2.199426 1.886449
H 2.278249 -3.157438 1.742491

ENERGIES
SCF ENERGY: -2026.45894295
SUM OF ELECTRONIC AND ZERO-POINT ENERGIES: -2026.052083
SUM OF ELECTRONIC AND THERMAL ENERGIES: -2026.016605
SUM OF ELECTRONIC AND THERMAL ENTHALPIES: -2026.015661
SUM OF ELECTRONIC AND THERMAL FREE ENERGIES: -2026.125350

==> AOX-II/HIS-/CU2+/BIS/RAF/C5 <==
57
DATASET
C -5.027071 -0.768487 0.303880
C -5.114287 -2.172728 0.388932
C -3.982795 -2.926439 0.578782

C -2.732570 -2.312654 0.694865
C -2.635437 -0.914556 0.603556
C -3.759971 -0.158382 0.411423
O -1.379919 -0.367036 0.725919
O -1.602280 -2.995294 0.887989
C -6.151361 0.070055 0.106357
C -7.466492 -0.321760 0.003469
C -8.480108 0.603354 -0.201310
C -9.902926 0.220089 -0.311880
C -10.822675 1.236135 0.378470
C -10.421444 2.568385 0.299811
C -9.167753 2.945371 -0.172809
O -8.192064 1.868204 -0.372486
O -11.864375 0.779952 0.874135
H -6.074312 -2.669715 0.301189
H -4.032702 -4.009032 0.648170
H -3.674016 0.923016 0.340245
H -5.938723 1.136098 0.035601
H -7.765070 -1.359921 0.084301
H -1.422200 0.599629 0.678744
H -1.781841 -3.946115 0.931651
H -11.085149 3.363786 0.619713
O -8.688780 4.033802 -0.400726
H -10.134950 0.344412 -1.390533
CU 0.170200 -1.055569 -0.316886
C 5.546736 -0.237642 -0.525652
C 5.331312 -1.618599 -0.586912
C 4.067462 -2.118596 -0.846270
C 3.004863 -1.255685 -1.052409
C 3.193074 0.123616 -0.999127
C 4.459203 0.617185 -0.734926
O 2.109458 0.916421 -1.214263
O 1.733448 -1.716214 -1.332473
C 6.848408 0.358590 -0.260480
C 7.989254 -0.301154 -0.009122
C 9.262891 0.332595 0.238936
C 10.401211 -0.348317 0.493578
C 11.650446 0.347036 0.731074
C 11.581672 1.753907 0.674273
C 10.401270 2.445830 0.408934
O 9.242783 1.688438 0.195533
O 12.712633 -0.296705 0.969281
H 6.151364 -2.312345 -0.432827
H 3.890302 -3.189713 -0.893768
H 4.600589 1.695482 -0.697848
H 6.870144 1.447130 -0.274365
H 8.017372 -1.388273 0.020292
H 2.363583 1.845805 -1.141579
H 1.734438 -2.681356 -1.405674
H 12.476862 2.345152 0.839388
O 10.222273 3.665616 0.331685
H 10.377043 -1.432764 0.519682
O -10.157868 -1.084674 0.107095
H -11.028489 -1.042747 0.541127

ENERGIES
SCF ENERGY: -2026.48220662
SUM OF ELECTRONIC AND ZERO-POINT ENERGIES: -2026.076104
SUM OF ELECTRONIC AND THERMAL ENERGIES: -2026.040652
SUM OF ELECTRONIC AND THERMAL ENTHALPIES: -2026.039707
SUM OF ELECTRONIC AND THERMAL FREE ENERGIES: -2026.149794

==> AOX-II/HIS-/CU2+/BIS/RAF/C5B <==
57
DATASET
C -5.352730 -0.688561 0.277726
C -5.491558 -2.032172 0.411570
C -4.308510 -2.906612 0.360483
C -2.998429 -2.263290 0.579160
C -2.885210 -0.880804 0.380753
C -4.027624 -0.141306 0.256663
O -1.641137 -0.337319 0.455993
O -1.974230 -2.978461 0.885601
C -6.464871 0.254902 0.200422
C -7.737844 -0.067287 -0.057278
C -8.823037 0.886216 -0.108020
C -10.097466 0.547808 -0.390770
C -11.145330 1.552498 -0.425396
C -10.728433 2.869000 -0.147874
C -9.406066 3.204764 0.142520
O -8.465011 2.166892 0.153275
O -12.337694 1.230265 -0.690876
H -6.456749 -2.526355 0.461587
H -3.923231 0.938865 0.167560
H -6.202496 1.298341 0.359874
H -8.030931 -1.095836 -0.256547
H -1.665153 0.627200 0.364230
H -2.255630 -3.899404 1.073206
H -11.453439 3.676717 -0.154229
O -8.930670 4.313952 0.399142
H -10.340098 -0.489944 -0.593699
CU 0.044123 -1.163487 -0.208481
C 5.469526 -0.254687 -0.369971
C 5.351819 -1.638790 -0.537397
C 4.108650 -2.221546 -0.703103
C 2.965779 -1.439792 -0.706786
C 3.057250 -0.058599 -0.552270
C 4.303515 0.518761 -0.380256
O 1.898549 0.654016 -0.575731
O 1.714102 -1.986017 -0.877918
C 6.746737 0.419685 -0.183164
C 7.955208 -0.163227 -0.171282
C 9.204349 0.535314 0.018452
C 10.408989 -0.075646 0.005650
C 11.631229 0.677772 0.200541
C 11.463336 2.062681 0.401545
C 10.214816 2.682177 0.415274
O 9.089211 1.872959 0.214543
O 12.756293 0.100204 0.185688
H 6.232528 -2.272463 -0.537221
H 4.008266 -3.296206 -0.830574
H 4.365651 1.597388 -0.250948
H 6.687378 1.497863 -0.042830
H 8.064054 -1.236545 -0.311453
H 2.081731 1.587884 -0.408027
H 1.748986 -2.952388 -0.847383
H 12.331973 2.694808 0.556740
O 9.949633 3.876742 0.584324
H 10.459063 -1.147548 -0.154608
H -4.217262 -3.075712 -0.748161
O -4.359638 -4.122548 1.041356
H -4.794053 -4.786573 0.492114

ENERGIES
SCF ENERGY: -2026.44143837
SUM OF ELECTRONIC AND ZERO-POINT ENERGIES: -2026.035337
SUM OF ELECTRONIC AND THERMAL ENERGIES: -2025.999688
SUM OF ELECTRONIC AND THERMAL ENTHALPIES: -2025.998743
SUM OF ELECTRONIC AND THERMAL FREE ENERGIES: -2026.107821

==> AOX-II/HIS-/CU2+/BIS/RAF/C6 <==
57
DATASET
C 5.038278 -0.715389 -0.616083
C 5.089165 -2.123600 -0.642006
C 3.933131 -2.857217 -0.736500
C 2.691888 -2.218577 -0.806747
C 2.629186 -0.816099 -0.757345
C 3.780440 -0.079884 -0.671915
O 1.382300 -0.240765 -0.821144

O	1.540032	-2.881825	-0.921216
C	6.194920	0.098867	-0.540157
C	7.499524	-0.332511	-0.463078
C	8.555655	0.564601	-0.397399
C	9.965515	0.131315	-0.300160
C	10.783992	1.043333	0.621705
C	10.438221	2.393047	0.603406
C	9.289781	2.862886	-0.027187
O	8.333329	1.850651	-0.487044
O	11.715812	0.501927	1.236595
H	6.041661	-2.639489	-0.591536
H	3.955807	-3.942649	-0.765377
H	3.721541	1.005315	-0.644610
H	6.019672	1.174053	-0.540040
H	7.755380	-1.384909	-0.442120
H	1.448771	0.724434	-0.776078
H	1.695986	-3.837357	-0.937134
H	11.060497	3.128201	1.101204
O	8.883943	3.986864	-0.222371
H	10.381920	0.333738	-1.309277
CU	-0.158182	-0.906134	0.251712
C	-5.554695	-0.207983	0.600992
C	-5.308720	-1.579584	0.726671
C	-4.025981	-2.040691	0.964146
C	-2.974104	-1.148601	1.085338
C	-3.194301	0.222607	0.976350
C	-4.478868	0.676513	0.731459
O	-2.123203	1.048935	1.116660
O	-1.683027	-1.571690	1.326673
C	-6.873096	0.349932	0.334959
C	-8.013970	-0.339625	0.183769
C	-9.300103	0.257576	-0.089571
C	-10.440624	-0.452049	-0.232197
C	-11.700472	0.208037	-0.510567
C	-11.638928	1.612376	-0.617727
C	-10.455698	2.333758	-0.470983
O	-9.287906	1.609720	-0.200140
O	-12.765686	-0.460537	-0.644552
H	-6.118068	-2.296509	0.634426
H	-3.822530	-3.104238	1.058250
H	-4.643337	1.748638	0.642925
H	-6.907655	1.435061	0.251759
H	-8.031548	-1.424661	0.260016
H	-2.401177	1.966976	0.998200
H	-1.656981	-2.534350	1.423070
H	-12.541624	2.177389	-0.827659
O	-10.280771	3.554144	-0.548397
H	-10.409520	-1.532459	-0.137440
O	10.106233	-1.213928	0.038677
H	10.889230	-1.245480	0.616786

ENERGIES
SCF ENERGY: -2026.48227121
SUM OF ELECTRONIC AND ZERO-POINT ENERGIES: -2026.075653
SUM OF ELECTRONIC AND THERMAL ENERGIES: -2026.040463
SUM OF ELECTRONIC AND THERMAL ENTHALPIES: -2026.039519
SUM OF ELECTRONIC AND THERMAL FREE ENERGIES: -2026.148606

==> AOX-II/HIS-/CU2+/BIS/RAF/C6B <==
57
DATASET

C	-4.702862	-0.489560	0.479800
C	-4.981183	-1.902852	0.059263
C	-3.883658	-2.871899	0.309520
C	-2.684665	-2.498007	0.764107
C	-2.448112	-1.115682	1.091177
C	-3.452034	-0.156061	0.936044
O	-1.264137	-0.839049	1.547939
O	-1.636735	-3.341550	0.997562
C	-5.692151	0.532170	0.371262
C	-6.891836	0.392716	-0.237491
C	-7.863365	1.442684	-0.353443
C	-9.039993	1.273198	-0.995351
C	-10.004666	2.357399	-1.088115
C	-9.608631	3.557263	-0.471157
C	-8.385506	3.718806	0.188562
O	-7.522851	2.616711	0.228864
O	-11.101572	2.188773	-1.687349
H	-4.092062	-3.914477	0.083506
H	-3.221437	0.869593	1.211832
H	-5.417317	1.498712	0.786606
H	-7.181251	-0.546305	-0.699982
H	-1.151007	0.106656	1.747660
H	-1.911541	-4.263401	0.877159
H	-10.268974	4.418249	-0.494641
O	-7.949443	4.721238	0.753137
H	-9.265546	0.311816	-1.444539
CU	0.140252	-3.052382	0.116945
C	5.026031	-0.619364	-0.395965
C	5.274752	-1.925509	-0.831490
C	4.234480	-2.823285	-0.993881
C	2.933452	-2.434192	-0.725236
C	2.661228	-1.139215	-0.289960
C	3.703884	-0.245434	-0.127952
O	1.357775	-0.838040	-0.044795
O	1.859943	-3.279169	-0.874529
C	6.076211	0.371020	-0.204163
C	7.385598	0.192773	-0.436601
C	8.402385	1.195796	-0.226275
C	9.716232	0.986897	-0.460096
C	10.692465	2.031343	-0.223948
C	10.171820	3.250907	0.254070
C	8.813515	3.455513	0.490702
O	7.944747	2.386923	0.234494
O	11.921715	1.827274	-0.441230
H	6.287520	-2.252786	-1.042310
H	4.420811	-3.840486	-1.327570
H	3.481285	0.762928	0.214445
H	5.740054	1.339239	0.163560
H	7.767554	-0.755288	-0.809002
H	1.282888	0.028837	0.376114
H	2.137050	-4.179827	-1.093304
H	10.840836	4.081641	0.455352
O	8.247698	4.471513	0.906780
H	10.043346	0.020061	-0.828116
H	-5.075462	-1.846441	-1.045505
O	-6.194623	-2.342881	0.632830
H	-6.504518	-3.118142	0.147426

ENERGIES
SCF ENERGY: -2026.45495463
SUM OF ELECTRONIC AND ZERO-POINT ENERGIES: -2026.048286
SUM OF ELECTRONIC AND THERMAL ENERGIES: -2026.012634
SUM OF ELECTRONIC AND THERMAL ENTHALPIES: -2026.011690
SUM OF ELECTRONIC AND THERMAL FREE ENERGIES: -2026.121486

==> AOX-II/HIS-/CU2+/MONO/BIDENTATE <==
34
DATASET

C	-0.396364	-0.394724	-0.073368
C	-0.015504	-1.734830	0.065200
C	1.318412	-2.104009	0.065012
C	2.285502	-1.126988	-0.071650
C	1.927246	0.204517	-0.216558
C	0.601562	0.578760	-0.214876
O	2.982253	1.081672	-0.366528
O	3.637513	-1.381056	-0.084799
C	-1.789085	0.035213	-0.061524
C	-2.858120	-0.774591	-0.070477
C	-4.232647	-0.334927	-0.028228

C -5.282802 -1.182205 -0.072751
C -6.644850 -0.689570 -0.014943
C -6.777136 0.709507 0.090811
C -5.684603 1.574892 0.136644
O -4.405499 1.006770 0.068622
O -7.621238 -1.491947 -0.056519
H -0.768113 -2.506411 0.188849
H 1.619287 -3.140319 0.183476
H 0.340175 1.628856 -0.316687
H -1.946029 1.112495 -0.045515
H -2.742727 -1.855544 -0.110910
H 2.756478 1.994799 -0.135523
H 3.877454 -2.213493 0.348001
H -7.764973 1.155998 0.145399
O -5.680647 2.805927 0.232653
H -5.100710 -2.249048 -0.149449
CU 4.840553 0.283036 0.048242
O 6.479124 -0.767449 0.412068
O 5.741038 2.060812 0.060194
H 5.247279 2.672159 -0.504556
H 6.621506 1.992211 -0.334880
H 6.454998 -1.594471 -0.089616
H 7.259304 -0.289397 0.097118

ENERGIES
SCF ENERGY: -1226.75274065
SUM OF ELECTRONIC AND ZERO-POINT ENERGIES: -1226.504969
SUM OF ELECTRONIC AND THERMAL ENERGIES: -1226.483328
SUM OF ELECTRONIC AND THERMAL ENTHALPIES: -1226.482383
SUM OF ELECTRONIC AND THERMAL FREE ENERGIES: -1226.556415

==> AOX-II/HIS-/CU2+/MONO/BIDENTATE/AEA <==
34
DATASET
C 0.374363 0.853147 -0.525415
C 0.890956 0.364971 -1.730896
C 2.242428 0.473144 -2.008492
C 3.104675 1.072115 -1.101245
C 2.606371 1.576574 0.103142
C 1.254476 1.464403 0.376944
O 3.498219 2.155207 0.954887
O 4.451149 1.171204 -1.310049
C -1.029117 0.741487 -0.152070
C -1.984302 0.098250 -0.841405
C -3.366612 0.001778 -0.435697
C -4.309494 -0.654713 -1.146201
C -5.681696 -0.720438 -0.685333
C -5.943897 -0.063036 0.533763
C -4.962869 0.611475 1.258641
O -3.664909 0.624777 0.732169
O -6.559807 -1.335908 -1.356098
H 0.238723 -0.106427 -2.459158
H 2.650854 0.089730 -2.939993
H 0.876762 1.856766 1.319099
H -1.299031 1.224580 0.785857
H -1.759364 -0.405731 -1.778999
H 3.043939 2.452466 1.754092
H 4.668130 0.856401 -2.198196
H -6.948685 -0.068120 0.944356
O -5.074978 1.217188 2.329381
H -4.031756 -1.139848 -2.076111
CU 3.320078 -1.456904 0.710472
O 1.493232 -2.085069 0.826024
O 5.148471 -0.837573 0.581804
H 5.407870 -0.430582 1.418587
H 5.144751 -0.109522 -0.069897
H 0.889816 -1.371113 0.569803
H 1.333013 -2.791916 0.187493

ENERGIES
SCF ENERGY: -1226.94582855
SUM OF ELECTRONIC AND ZERO-POINT ENERGIES: -1226.700023
SUM OF ELECTRONIC AND THERMAL ENERGIES: -1226.677644
SUM OF ELECTRONIC AND THERMAL ENTHALPIES: -1226.676700
SUM OF ELECTRONIC AND THERMAL FREE ENERGIES: -1226.753223

==> AOX-II/HIS-/CU2+/MONO/BIDENTATE/AIP <==
34
DATASET
C -0.270200 -0.095939 -0.454087
C 0.161864 -1.469251 -0.573344
C 1.459494 -1.764584 -0.810560
C 2.396896 -0.716750 -0.957167
C 1.994181 0.663168 -0.819615
C 0.685570 0.945452 -0.572308
O 2.979005 1.546444 -0.937851
O 3.652143 -0.919610 -1.192699
C -1.601833 0.244621 -0.241719
C -2.646582 -0.671166 -0.129276
C -3.982250 -0.294250 0.027455
C -4.973536 -1.249409 0.098984
C -6.371228 -0.900605 0.224256
C -6.588742 0.491042 0.271347
C -5.560121 1.469588 0.208774
O -4.248513 1.025682 0.083610
O -7.266223 -1.774277 0.276738
H -0.553971 -2.276078 -0.475300
H 1.808574 -2.787161 -0.906500
H 0.366247 1.978221 -0.468997
H -1.839739 1.303180 -0.173484
H -2.468294 -1.740781 -0.174037
H 2.653116 2.451309 -0.812589
H 3.872790 -1.867428 -1.266016
H -7.599252 0.875524 0.363526
O -5.705735 2.674789 0.252962
H -4.681910 -2.293939 0.047914
CU 4.994053 0.101962 0.787925
O 3.665280 -0.540574 2.044868
O 6.291169 0.799499 -0.473058
H 5.926881 0.729697 -1.365652
H 7.102639 0.275980 -0.480849
H 3.424314 -1.453830 1.839665
H 4.002971 -0.558724 2.949503

ENERGIES
SCF ENERGY: -1226.52677405
SUM OF ELECTRONIC AND ZERO-POINT ENERGIES: -1226.279862
SUM OF ELECTRONIC AND THERMAL ENERGIES: -1226.257235
SUM OF ELECTRONIC AND THERMAL ENTHALPIES: -1226.256291
SUM OF ELECTRONIC AND THERMAL FREE ENERGIES: -1226.333578

==> AOX-II/HIS-/CU2+/MONO/BIDENTATE/HAT/C3B <==
33
DATASET
C 0.387569 -0.431037 -0.077167
C 0.080934 -1.852319 -0.019219
C -1.188751 -2.331660 -0.029632
C -2.253322 -1.419692 -0.090081
C -1.993777 0.046093 -0.158077
C -0.641461 0.469277 -0.147060
O -2.954256 0.838883 -0.229856
O -3.492005 -1.759825 -0.089799
C 1.746520 0.024930 -0.065364
C 2.840179 -0.768081 0.006768
C 4.189058 -0.282933 0.017474
C 5.256914 -1.110533 0.096330
C 6.611942 -0.588030 0.107640
C 6.705844 0.813762 0.027593
C 5.592579 1.658514 -0.055610

O 4.326315 1.061231 -0.056173
O 7.599854 -1.367856 0.184598
H 0.895844 -2.566706 0.034617
H -1.404806 -3.393837 0.016016
H -0.448844 1.536178 -0.194915
H 1.880338 1.102990 -0.119206
H 2.759238 -1.849571 0.064218
H -3.625728 -2.725897 -0.043468
H 7.681321 1.289398 0.028321
O 5.571860 2.885613 -0.130835
H 5.092521 -2.181345 0.153149
CU -4.836116 0.652538 -0.017368
O -6.485309 0.520601 -1.232958
O -5.916243 0.792295 1.734690
H -5.462023 0.356589 2.465679
H -6.748861 0.315117 1.631157
H -6.348704 -0.129155 -1.932938
H -7.230025 0.185374 -0.717854

ENERGIES
SCF ENERGY: -1226.10384023
SUM OF ELECTRONIC AND ZERO-POINT ENERGIES: -1225.870523
SUM OF ELECTRONIC AND THERMAL ENERGIES: -1225.848655
SUM OF ELECTRONIC AND THERMAL ENTHALPIES: -1225.847711
SUM OF ELECTRONIC AND THERMAL FREE ENERGIES: -1225.923422

==> AOX-II/HIS-/CU2+/MONO/BIDENTATE/HAT/C4B <==
33
DATASET
C 0.476366 -0.132018 0.120228
C 0.036216 -1.508875 0.231765
C -1.270764 -1.813549 0.268098
C -2.277542 -0.778106 0.193689
C -1.814897 0.619603 0.072501
C -0.478521 0.900962 0.055557
O -2.779361 1.510927 -0.009661
O -3.482165 -1.057780 0.241509
C 1.839884 0.218591 0.069946
C 2.873289 -0.680214 0.053358
C 4.243787 -0.315954 0.001583
C 5.227005 -1.254370 -0.039108
C 6.629274 -0.877669 -0.090481
C 6.854345 0.509502 -0.094892
C 5.828792 1.470300 -0.051280
O 4.510505 1.009076 -0.005312
O 7.529085 -1.756591 -0.124623
H 0.771486 -2.302112 0.295022
H -1.625605 -2.834556 0.355457
H -0.149577 1.933131 -0.027190
H 2.071573 1.279569 0.027112
H 2.692352 -1.751041 0.070028
H -2.427128 2.412965 -0.088067
H 7.870032 0.889803 -0.130611
O 5.943545 2.689942 -0.047947
H 4.951531 -2.303783 -0.030930
CU -5.112599 -0.103045 0.001244
O -6.182405 0.111393 -1.769927
O -6.499340 0.843252 1.185154
H -6.572463 0.425089 2.051280
H -7.372748 0.752214 0.784207
H -6.152458 -0.698649 -2.292854
H -7.113934 0.234681 -1.548875

ENERGIES
SCF ENERGY: -1226.11702979
SUM OF ELECTRONIC AND ZERO-POINT ENERGIES: -1225.883376
SUM OF ELECTRONIC AND THERMAL ENERGIES: -1225.860719
SUM OF ELECTRONIC AND THERMAL ENTHALPIES: -1225.859774
SUM OF ELECTRONIC AND THERMAL FREE ENERGIES: -1225.937205

==> AOX-II/HIS-/CU2+/MONO/BIDENTATE/HAT/H2O <==
33
DATASET
C 0.409215 -0.272791 0.139040
C -0.012351 -1.641199 0.218941
C -1.334081 -1.963249 0.254364
C -2.284371 -0.931136 0.241087
C -1.891151 0.431469 0.079386
C -0.562965 0.747646 0.077632
O -2.902299 1.293905 -0.059844
O -3.573159 -1.131037 0.435659
C 1.765442 0.103797 0.077026
C 2.834984 -0.760132 0.048619
C 4.177744 -0.328423 -0.008009
C 5.212590 -1.222866 -0.037873
C 6.593073 -0.790743 -0.094800
C 6.749571 0.609445 -0.115006
C 5.674379 1.524883 -0.083681
O 4.381798 1.005876 -0.030005
O 7.535522 -1.619827 -0.120573
H 0.724584 -2.433887 0.272519
H -1.675927 -2.988865 0.344888
H -0.257016 1.785018 -0.018487
H 1.970744 1.170956 0.041359
H 2.698329 -1.837360 0.066491
H -2.613864 2.219892 -0.030533
H -3.815911 -2.070893 0.528578
H 7.744126 1.041162 -0.157031
O 5.742186 2.743368 -0.098216
H 4.981415 -2.282891 -0.016626
CU -4.996658 0.285772 -0.007561
O -6.300015 -1.039869 -1.004828
O -6.110545 1.766971 0.273759
H -5.540441 2.451098 0.638934
H -6.307834 -1.866322 -0.505014
H -7.185512 -0.670589 -0.893771

ENERGIES
SCF ENERGY: -1226.04941674
SUM OF ELECTRONIC AND ZERO-POINT ENERGIES: -1225.816252
SUM OF ELECTRONIC AND THERMAL ENERGIES: -1225.794071
SUM OF ELECTRONIC AND THERMAL ENTHALPIES: -1225.793126
SUM OF ELECTRONIC AND THERMAL FREE ENERGIES: -1225.868384

==> AOX-II/HIS-/CU2+/MONO/BIDENTATE/RAF/C1B <==
36
DATASET
C 0.587111 -0.277369 -0.826587
C 0.781769 0.970750 -1.617622
C 1.277657 2.094531 -1.081546
C 1.679227 2.109058 0.287945
C 1.644219 0.926444 1.092708
C 1.203825 -0.222578 0.536381
O 2.097925 1.072355 2.352515
O 2.113024 3.178999 0.866390
C -0.883699 -0.509756 -0.532939
C -1.862878 0.388455 -0.640386
C -3.244679 0.126192 -0.292979
C -4.217871 1.052514 -0.389168
C -5.585007 0.738356 -0.010004
C -5.797651 -0.579688 0.437262
C -4.779172 -1.529281 0.527235
O -3.489892 -1.130130 0.148408
O -6.489986 1.615752 -0.087339
H 0.465867 0.933160 -2.656528
H 1.392169 3.012457 -1.650332
H 1.171122 -1.141045 1.119172
H -1.075714 -1.515799 -0.164537
H -1.673452 1.398898 -0.996539

H	2.013238	0.237355	2.834431
H	2.113922	3.954284	0.277059
H	-6.792330	-0.892870	0.738360
O	-4.849747	-2.700833	0.905555
H	-3.974317	2.046840	-0.748143
CU	3.974095	-0.897074	-0.056475
O	3.708292	-0.639372	-1.959915
O	4.242753	-1.196955	1.836880
H	4.267364	-0.356997	2.313966
H	5.098605	-1.613418	2.001953
H	3.739599	0.294737	-2.207619
H	4.367914	-1.089760	-2.503843
O	1.023444	-1.410219	-1.552425
H	1.973640	-1.295229	-1.741984

ENERGIES
SCF ENERGY: -1302.54573062
SUM OF ELECTRONIC AND ZERO-POINT ENERGIES: -1302.282127
SUM OF ELECTRONIC AND THERMAL ENERGIES: -1302.258847
SUM OF ELECTRONIC AND THERMAL ENTHALPIES: -1302.257903
SUM OF ELECTRONIC AND THERMAL FREE ENERGIES: -1302.335760

==> AOX-II/HIS-/CU2+/MONO/BIDENTATE/RAF/C1P <==
36
DATASET

C	-0.404522	1.724242	-0.402240
C	-0.662456	1.869152	1.000670
C	-1.906022	1.633417	1.494872
C	-2.954325	1.264937	0.631064
C	-2.733050	1.129260	-0.769215
C	-1.487924	1.348390	-1.265268
O	-3.810351	0.754010	-1.496448
O	-4.170898	1.037199	1.058079
C	0.838648	1.847806	-0.961136
C	2.150404	2.152451	-0.340183
C	2.880388	0.836316	-0.186755
C	4.062982	0.543235	-0.738004
C	4.689906	-0.747475	-0.473449
C	3.956481	-1.615867	0.364044
C	2.715398	-1.287579	0.895490
O	2.209598	-0.016087	0.614425
O	5.804143	-1.032586	-0.982175
H	0.144757	2.155986	1.660486
H	-2.125093	1.726482	2.553909
H	-1.302402	1.228580	-2.329519
H	0.922839	1.630864	-2.026117
H	2.720768	2.742791	-1.072980
H	-3.581496	0.700852	-2.435342
H	-4.238823	1.155046	2.020295
H	4.346785	-2.603542	0.586444
O	1.953033	-1.978341	1.589232
H	4.560138	1.267302	-1.374069
CU	-2.115453	-1.863727	-0.112350
O	-0.201642	-1.884604	-0.149821
O	-4.033360	-1.870655	-0.358085
H	-4.243808	-1.032827	-0.810084
H	-4.479710	-1.816114	0.496846
H	0.132310	-1.129130	-0.650610
H	0.356646	-1.929439	0.655800
O	2.040335	2.831860	0.886109
H	2.932915	3.035159	1.193237

ENERGIES
SCF ENERGY: -1302.57558015
SUM OF ELECTRONIC AND ZERO-POINT ENERGIES: -1302.310564
SUM OF ELECTRONIC AND THERMAL ENERGIES: -1302.287952
SUM OF ELECTRONIC AND THERMAL ENTHALPIES: -1302.287007
SUM OF ELECTRONIC AND THERMAL FREE ENERGIES: -1302.362476

==> AOX-II/HIS-/CU2+/MONO/BIDENTATE/RAF/C2 <==
36
DATASET

C	0.578851	-0.876695	-0.553671
C	1.092089	-1.905797	0.243875
C	2.437596	-2.218147	0.198580
C	3.297145	-1.517957	-0.636509
C	2.804362	-0.486005	-1.440969
C	1.455281	-0.184525	-1.399880
O	3.698142	0.172209	-2.229471
O	4.637397	-1.766011	-0.685918
C	-0.818492	-0.490585	-0.545775
C	-1.782656	-1.015907	0.232681
C	-3.156044	-0.601435	0.201265
C	-4.099844	-1.142160	1.024048
C	-5.480372	-0.786371	0.948412
C	-5.774029	0.391402	0.078462
C	-4.688337	0.928963	-0.731308
O	-3.436070	0.341639	-0.730532
O	-6.394207	-1.383256	1.518269
H	0.442225	-2.468583	0.905713
H	2.846191	-3.012740	0.817205
H	1.078420	0.619413	-2.030067
H	-1.086699	0.304701	-1.239491
H	-1.568196	-1.804243	0.948846
H	3.247493	0.869822	-2.722971
H	4.834347	-2.580671	-0.202747
H	-6.809816	0.554484	-0.211611
O	-5.041292	1.601425	0.434313
H	-3.794166	-1.927880	1.705819
CU	3.699275	1.259286	0.983699
O	5.259763	0.168425	1.286574
O	2.141605	2.366675	0.730264
H	1.874994	2.372757	-0.198545
H	2.326785	3.288301	0.952353
H	5.310288	-0.484190	0.562509
H	6.066481	0.694843	1.224683
O	-4.809538	1.563877	-1.919632
H	-5.687018	1.966151	-1.985154

ENERGIES
SCF ENERGY: -1302.56623360
SUM OF ELECTRONIC AND ZERO-POINT ENERGIES: -1302.304205
SUM OF ELECTRONIC AND THERMAL ENERGIES: -1302.280290
SUM OF ELECTRONIC AND THERMAL ENTHALPIES: -1302.279345
SUM OF ELECTRONIC AND THERMAL FREE ENERGIES: -1302.360318

==> AOX-II/HIS-/CU2+/MONO/BIDENTATE/RAF/C2B <==
36
DATASET

C	-0.667568	-0.197168	-0.464139
C	-0.124049	-1.452005	-0.591823
C	1.256853	-1.640271	-0.727005
C	2.185682	-0.610225	-0.713299
C	1.707989	0.679091	-0.548537
C	0.257659	0.959288	-0.660781
O	2.522128	1.683539	-0.377875
O	3.522762	-0.779637	-0.768896
C	-2.058982	0.096324	-0.299443
C	-3.007679	-0.825561	-0.031766
C	-4.405709	-0.526978	0.113214
C	-5.330583	-1.469634	0.397173
C	-6.737343	-1.125003	0.516095
C	-7.036773	0.234894	0.314748
C	-6.068907	1.199851	0.022360
O	-4.738495	0.773438	-0.069583
O	-7.594587	-2.012427	0.780824
H	-0.756897	-2.332410	-0.577045
H	1.638791	-2.655121	-0.813602
H	0.174463	1.103786	-1.767548

H -2.354071 1.138990 -0.378365
H -2.754370 -1.874712 0.101390
H 2.002564 2.489129 -0.174996
H 3.754872 -1.718674 -0.751438
H -8.064749 0.576214 0.383212
O -6.225489 2.406142 -0.171483
H -5.015440 -2.498603 0.534114
CU 5.986609 -0.052485 0.536519
O 6.784028 -1.760621 0.107157
O 5.093031 1.596575 0.997158
H 4.248202 1.630568 0.515797
H 5.597842 2.358768 0.686695
H 6.359408 -2.124875 -0.680518
H 7.716682 -1.662636 -0.123557
O -0.038165 2.148043 0.011626
H -0.584216 2.715612 -0.545389

ENERGIES
SCF ENERGY: -1302.55095273
SUM OF ELECTRONIC AND ZERO-POINT ENERGIES: -1302.289107
SUM OF ELECTRONIC AND THERMAL ENERGIES: -1302.264853
SUM OF ELECTRONIC AND THERMAL ENTHALPIES: -1302.263909
SUM OF ELECTRONIC AND THERMAL FREE ENERGIES: -1302.345745

==> AOX-II/HIS-/CU2+/MONO/BIDENTATE/RAF/C2P <==
36
DATASET
C -0.178094 0.220480 0.345817
C 0.110341 0.044543 -1.005268
C 1.199153 0.697923 -1.566409
C 2.009663 1.504625 -0.786738
C 1.741001 1.657571 0.581712
C 0.653741 1.011702 1.137981
O 2.583198 2.447560 1.292094
O 3.103684 2.151039 -1.269388
C -0.954667 -1.889981 0.850114
C -1.360304 -0.493519 1.006581
C -2.730087 -0.131322 0.521744
C -3.714252 0.257680 1.341337
C -5.023595 0.603411 0.803454
C -5.148638 0.496714 -0.594805
C -4.108738 0.084478 -1.426330
O -2.882708 -0.225709 -0.812808
O -5.953949 0.967613 1.572883
H -0.509099 -0.593532 -1.626301
H 1.436042 0.579448 -2.620158
H 0.449535 1.143063 2.197917
H -1.303765 -0.279525 2.081231
H 2.308878 2.470611 2.218572
H 3.173454 2.016031 -2.224768
H -6.094496 0.736846 -1.069921
O -4.101685 -0.054499 -2.651008
H -3.538794 0.318089 2.409837
CU 3.687982 -0.935413 0.087065
O 5.006248 0.478629 0.039705
O 2.509496 -2.470305 0.077250
H 1.720026 -2.284352 -0.450029
H 2.183074 -2.652688 0.968066
H 4.568064 1.230287 -0.403239
H 5.191853 0.784184 0.936897
H -0.088888 -2.255429 1.409253
O -1.548951 -2.659807 0.051150
H -1.143160 -3.548056 -0.006410

ENERGIES
SCF ENERGY: -1302.57354727
SUM OF ELECTRONIC AND ZERO-POINT ENERGIES: -1302.308762
SUM OF ELECTRONIC AND THERMAL ENERGIES: -1302.285701
SUM OF ELECTRONIC AND THERMAL ENTHALPIES: -1302.284757
SUM OF ELECTRONIC AND THERMAL FREE ENERGIES: -1302.362300

==> AOX-II/HIS-/CU2+/MONO/BIDENTATE/RAF/C3 <==
36
DATASET
C 0.078391 -0.925374 -0.373294
C 0.406952 -2.230131 0.012923
C 1.715944 -2.667427 -0.052118
C 2.720761 -1.825153 -0.508459
C 2.409350 -0.524060 -0.914437
C 1.101315 -0.084993 -0.832209
O 3.428404 0.245747 -1.379458
O 4.028839 -2.203961 -0.568074
C -1.269796 -0.398961 -0.315579
C -2.381436 -1.096623 -0.010926
C -3.693910 -0.532123 0.045686
C -4.806037 -1.246553 0.346440
C -6.100421 -0.638417 0.393827
C -6.166044 0.738388 -0.243161
C -4.894229 1.527477 -0.082296
O -3.731787 0.825004 -0.181959
O -7.109328 -1.168398 0.836222
H -0.357032 -2.906313 0.381781
H 1.982593 -3.674045 0.258726
H 0.870637 0.932699 -1.140478
H -1.368159 0.659565 -0.550241
H -2.342472 -2.160646 0.206758
H 3.111890 1.142722 -1.549790
H 4.112475 -3.139161 -0.333928
H -6.201100 0.520859 -1.330005
O -4.842049 2.714762 0.045224
H -4.702410 -2.295936 0.597613
CU 5.351519 1.100830 0.592113
O 5.295356 -0.641571 1.411889
O 5.240695 2.840065 -0.238163
H 5.016906 2.739225 -1.172670
H 6.085564 3.307405 -0.222810
H 4.963927 -1.265083 0.734855
H 6.181193 -0.948366 1.641172
O -7.291332 1.436459 0.186202
H -7.423501 2.201835 -0.385700

ENERGIES
SCF ENERGY: -1302.60330666
SUM OF ELECTRONIC AND ZERO-POINT ENERGIES: -1302.340398
SUM OF ELECTRONIC AND THERMAL ENERGIES: -1302.316509
SUM OF ELECTRONIC AND THERMAL ENTHALPIES: -1302.315565
SUM OF ELECTRONIC AND THERMAL FREE ENERGIES: -1302.395494

==> AOX-II/HIS-/CU2+/MONO/BIDENTATE/RAF/C3B <==
36
DATASET
C 0.581103 -0.632139 -0.008862
C 0.319043 -2.051137 -0.104601
C -0.929326 -2.600541 -0.154876
C -2.037613 -1.761289 -0.121586
C -1.893684 -0.249845 -0.011710
C -0.472611 0.205181 0.053793
O -2.515824 0.227643 -1.171251
O -3.256625 -2.162492 -0.168914
C 1.939765 -0.114165 0.033943
C 3.060805 -0.843333 -0.043869
C 4.394690 -0.287417 -0.005108
C 5.512066 -1.034643 -0.113367
C 6.826460 -0.418623 -0.080342
C 6.832473 0.981106 0.077757
C 5.668380 1.740848 0.193732
O 4.445694 1.058278 0.144247
O 7.870014 -1.121656 -0.191631
H 1.164954 -2.732578 -0.135203

H -1.078001 -3.672794 -0.223773
H -0.343075 1.281793 0.140909
H 2.012786 0.966333 0.134019
H 3.034535 -1.924909 -0.151053
H -2.305985 1.167529 -1.280437
H -3.346128 -3.132864 -0.241550
H 7.776532 1.515417 0.113296
O 5.554161 2.960837 0.337687
H 5.424608 -2.109450 -0.232192
CU -5.424253 0.851382 0.010739
O -5.964275 -0.863796 0.700763
O -4.857030 2.543166 -0.722210
H -3.931799 2.706502 -0.496756
H -5.349326 3.282228 -0.342377
H -5.242159 -1.493721 0.561270
H -6.080548 -0.813011 1.658541
O -2.501003 0.196833 1.164197
H -3.443255 -0.045073 1.151342

ENERGIES
SCF ENERGY: -1302.54383958
SUM OF ELECTRONIC AND ZERO-POINT ENERGIES: -1302.280076
SUM OF ELECTRONIC AND THERMAL ENERGIES: -1302.256376
SUM OF ELECTRONIC AND THERMAL ENTHALPIES: -1302.255432
SUM OF ELECTRONIC AND THERMAL FREE ENERGIES: -1302.335650

==> AOX-II/HIS-/CU2+/MONO/BIDENTATE/RAF/C4 <==
36
DATASET
C -0.044204 -0.873656 0.322993
C -0.333949 -2.194465 -0.033975
C -1.630092 -2.671859 0.043009
C -2.661278 -1.853872 0.480666
C -2.389438 -0.535845 0.852749
C -1.093826 -0.058264 0.761233
O -3.431146 0.218909 1.297196
O -3.960444 -2.274730 0.553396
C 1.293080 -0.302796 0.258538
C 2.424709 -0.967309 -0.020915
C 3.733274 -0.369361 -0.086646
C 4.864444 -1.040141 -0.336883
C 6.149989 -0.345277 -0.299188
C 6.127746 1.117538 -0.161300
C 4.808680 1.792642 -0.066648
O 3.725119 1.005411 0.124930
O 6.465839 0.274437 0.942373
H 0.450098 -2.856028 -0.387665
H -1.863165 -3.694203 -0.243494
H -0.897263 0.972912 1.048354
H 1.352499 0.764047 0.466902
H 2.420002 -2.038020 -0.212178
H -3.130531 1.121740 1.465164
H -4.013109 -3.216971 0.340111
H 6.934584 1.730885 -0.555139
O 4.653369 2.981902 -0.149742
H 4.832859 -2.105537 -0.530946
CU -5.261069 1.031942 -0.545497
O -5.215707 -0.699735 -1.401498
O -5.260806 2.777959 0.299477
H -4.896222 2.714429 1.191869
H -6.157572 3.118309 0.410754
H -4.897533 -1.342605 -0.735864
H -6.103188 -0.989363 -1.645317
O 7.159455 -1.011898 -0.932124
H 7.959294 -0.467974 -0.946995

ENERGIES
SCF ENERGY: -1302.56306286
SUM OF ELECTRONIC AND ZERO-POINT ENERGIES: -1302.300129
SUM OF ELECTRONIC AND THERMAL ENERGIES: -1302.276646
SUM OF ELECTRONIC AND THERMAL ENTHALPIES: -1302.275702
SUM OF ELECTRONIC AND THERMAL FREE ENERGIES: -1302.354680

==> AOX-II/HIS-/CU2+/MONO/BIDENTATE/RAF/C4B <==
36
DATASET
C -0.700350 -0.381984 -0.152769
C -0.351007 -1.750890 0.169759
C 0.922811 -2.142048 0.202645
C 2.067479 -1.219019 -0.068245
C 1.624644 0.181364 -0.430111
C 0.309480 0.545455 -0.450418
O 2.626347 0.972288 -0.699966
O 2.859033 -1.073776 1.079498
C -2.043874 0.071984 -0.178812
C -3.124453 -0.702826 0.104788
C -4.473120 -0.231824 0.094815
C -5.516602 -1.039101 0.397046
C -6.881748 -0.535156 0.388701
C -7.002692 0.822784 0.048444
C -5.912142 1.646893 -0.262826
O -4.637308 1.071253 -0.229057
O -7.845484 -1.295008 0.672880
H -1.135454 -2.466068 0.385288
H 1.205314 -3.164924 0.444065
H 0.046509 1.568760 -0.702741
H -2.198010 1.116391 -0.436165
H -3.020038 -1.751132 0.369182
H 2.329547 1.870777 -0.924577
H 2.975969 -1.938790 1.498100
H -7.984587 1.283984 0.017919
O -5.923245 2.836001 -0.569117
H -5.327584 -2.076822 0.650729
CU 5.651808 0.627940 0.150597
O 5.960143 1.739029 1.691922
O 5.331504 -0.441727 -1.412401
H 4.463605 -0.889422 -1.340805
H 5.986596 -1.146239 -1.487920
H 5.962264 1.215282 2.503164
H 6.829684 2.157275 1.652679
O 2.815338 -1.677031 -1.169583
H 2.962553 -2.631357 -1.087754

ENERGIES
SCF ENERGY: -1302.55918596
SUM OF ELECTRONIC AND ZERO-POINT ENERGIES: -1302.296487
SUM OF ELECTRONIC AND THERMAL ENERGIES: -1302.272655
SUM OF ELECTRONIC AND THERMAL ENTHALPIES: -1302.271711
SUM OF ELECTRONIC AND THERMAL FREE ENERGIES: -1302.351672

==> AOX-II/HIS-/CU2+/MONO/BIDENTATE/RAF/C5 <==
36
DATASET
C 0.533145 0.327007 -1.059606
C 1.041063 -0.920572 -1.477019
C 2.384117 -1.073026 -1.713798
C 3.260402 0.002788 -1.540254
C 2.771532 1.256163 -1.121704
C 1.426667 1.408242 -0.895778
O 3.688406 2.242034 -0.955604
O 4.577594 -0.091379 -1.747264
C -0.836544 0.546402 -0.788351
C -1.852270 -0.383808 -0.862386
C -3.162264 -0.048749 -0.567782
C -4.274763 -1.019653 -0.639324
C -5.276887 -0.822980 0.505005
C -5.485854 0.494162 0.907352
C -4.687565 1.546666 0.465669
O -3.476131 1.189888 -0.268738

O -5.850817 -1.846598 0.909234
H 0.375171 -1.765030 -1.616264
H 2.794547 -2.026043 -2.035550
H 1.047425 2.372867 -0.565919
H -1.103673 1.557568 -0.483554
H -1.666178 -1.413220 -1.144170
H 3.250656 3.050948 -0.658630
H 4.822176 -0.992981 -2.003170
H -6.285275 0.739829 1.597339
O -4.780550 2.743479 0.633102
H -4.843067 -0.719148 -1.544434
CU 3.424945 -0.427201 1.575770
O 4.371543 -2.036083 1.090094
O 2.404121 1.087388 2.212473
H 2.663298 1.900063 1.757872
H 2.580185 1.248920 3.148465
H 4.558439 -2.079190 0.143180
H 5.234653 -2.071247 1.522376
O -3.847747 -2.343788 -0.733918
H -4.487792 -2.849863 -0.202036

ENERGIES
SCF ENERGY: -1302.58546177
SUM OF ELECTRONIC AND ZERO-POINT ENERGIES: -1302.322741
SUM OF ELECTRONIC AND THERMAL ENERGIES: -1302.299054
SUM OF ELECTRONIC AND THERMAL ENTHALPIES: -1302.298110
SUM OF ELECTRONIC AND THERMAL FREE ENERGIES: -1302.377993

==> AOX-II/HIS-/CU2+/MONO/BIDENTATE/RAF/C5B <==
36
DATASET
C 0.318600 -0.640105 -0.635673
C 0.793001 -1.758234 -0.030294
C 2.168163 -2.209775 -0.301493
C 3.091978 -1.194814 -0.844302
C 2.564927 -0.087691 -1.536480
C 1.228010 0.169734 -1.395161
O 3.447839 0.699134 -2.180932
O 4.364242 -1.368740 -0.757761
C -1.063855 -0.178701 -0.533492
C -2.105254 -0.940235 -0.177499
C -3.464719 -0.461900 -0.065261
C -4.505203 -1.251069 0.271338
C -5.849952 -0.712194 0.366788
C -5.970497 0.662177 0.083022
C -4.885165 1.466866 -0.263495
O -3.625054 0.858027 -0.327934
O -6.820308 -1.456301 0.684285
H 0.173599 -2.414742 0.572581
H 0.837741 1.071556 -1.865083
H -1.229668 0.865714 -0.789768
H -1.979601 -1.998751 0.041270
H 3.013332 1.497051 -2.516417
H 4.554209 -2.127490 -0.166169
H -6.943987 1.139621 0.132572
O -4.873019 2.672172 -0.527751
H -4.331468 -2.302733 0.473444
CU 2.932680 1.372689 1.135487
O 2.706253 -0.306964 2.048848
O 3.200789 2.945648 0.051343
H 3.862536 2.758502 -0.627941
H 3.554867 3.683478 0.564121
H 3.347806 -0.423069 2.761396
H 1.836929 -0.381468 2.463780
H 2.015499 -2.800102 -1.245538
O 2.801227 -2.996771 0.661132
H 2.704520 -3.929528 0.434815

ENERGIES
SCF ENERGY: -1302.54797968
SUM OF ELECTRONIC AND ZERO-POINT ENERGIES: -1302.286296
SUM OF ELECTRONIC AND THERMAL ENERGIES: -1302.262304
SUM OF ELECTRONIC AND THERMAL ENTHALPIES: -1302.261359
SUM OF ELECTRONIC AND THERMAL FREE ENERGIES: -1302.341162

==> AOX-II/HIS-/CU2+/MONO/BIDENTATE/RAF/C6 <==
36
DATASET
C 0.427026 -0.528469 -0.601271
C 0.859289 -1.756847 -0.064103
C 2.150949 -2.179120 -0.263549
C 3.042397 -1.400679 -1.003098
C 2.632420 -0.168878 -1.545967
C 1.339470 0.250734 -1.342893
O 3.560636 0.531506 -2.244928
O 4.317466 -1.764102 -1.212077
C -0.893351 -0.039797 -0.438009
C -1.938870 -0.688266 0.178470
C -3.197057 -0.108073 0.274409
C -4.353828 -0.823820 0.881089
C -5.686773 -0.464741 0.204199
C -5.771862 0.836281 -0.305111
C -4.688321 1.698041 -0.402234
O -3.371041 1.123169 -0.125860
O -6.603297 -1.295364 0.234929
H 0.180157 -2.374298 0.513173
H 2.505494 -3.119551 0.148399
H 1.019474 1.202634 -1.761697
H -1.092157 0.942275 -0.866143
H -1.828730 -1.685055 0.588539
H 3.171637 1.348500 -2.585008
H 4.474055 -2.659088 -0.875537
H -6.727782 1.214154 -0.651207
O -4.610892 2.862044 -0.734688
H -4.456095 -0.382706 1.890853
CU 3.849138 0.924440 1.106614
O 4.949048 -0.618286 1.463873
O 2.751627 2.476893 0.774431
H 2.647155 2.611416 -0.177192
H 3.177565 3.279347 1.102671
H 5.206952 -1.011942 0.615995
H 5.774790 -0.363050 1.894834
O -4.173007 -2.207450 0.949862
H -3.770755 -2.436881 1.794589

ENERGIES
SCF ENERGY: -1302.58221450
SUM OF ELECTRONIC AND ZERO-POINT ENERGIES: -1302.320188
SUM OF ELECTRONIC AND THERMAL ENERGIES: -1302.296225
SUM OF ELECTRONIC AND THERMAL ENTHALPIES: -1302.295281
SUM OF ELECTRONIC AND THERMAL FREE ENERGIES: -1302.374756

==> AOX-II/HIS-/CU2+/MONO/BIDENTATE/RAF/C6B <==
36
DATASET
C -0.718061 -0.107625 0.100636
C -0.289339 -1.539243 0.212050
C 1.172606 -1.764510 0.336075
C 2.075509 -0.780754 0.206401
C 1.600469 0.565577 0.013692
C 0.238617 0.869078 -0.025203
O 2.526813 1.478185 -0.110806
O 3.414804 -0.906084 0.261872
C -2.094990 0.262380 0.061065
C -3.126200 -0.604105 -0.059148
C -4.506302 -0.215500 -0.124197
C -5.501702 -1.111190 -0.303611
C -6.890594 -0.685110 -0.361570
C -7.091796 0.699052 -0.216786

C -6.050874 1.614812 -0.028937
O -4.745480 1.110702 0.009478
O -7.812445 -1.529522 -0.529195
H 1.493988 -2.789249 0.504157
H -0.051454 1.908745 -0.151211
H -2.299115 1.330165 0.085656
H -2.952404 -1.673320 -0.137843
H 2.159572 2.371253 -0.233245
H 3.659778 -1.828189 0.421936
H -8.098814 1.102783 -0.245885
O -6.124990 2.835054 0.111678
H -5.256727 -2.163302 -0.403445
CU 6.334189 0.343491 -0.127415
O 7.484963 -0.752575 -1.214327
O 5.228232 1.426319 1.015647
H 4.312917 1.401050 0.687935
H 5.492826 2.354033 0.982658
H 7.103025 -1.629190 -1.350528
H 7.582857 -0.373780 -2.097366
H -0.571308 -1.980929 -0.767677
O -1.002269 -2.172125 1.254874
H -0.927721 -3.128107 1.143316

ENERGIES
SCF ENERGY: -1302.55546568
SUM OF ELECTRONIC AND ZERO-POINT ENERGIES: -1302.293075
SUM OF ELECTRONIC AND THERMAL ENERGIES: -1302.268661
SUM OF ELECTRONIC AND THERMAL ENTHALPIES: -1302.267716
SUM OF ELECTRONIC AND THERMAL FREE ENERGIES: -1302.351009

==> AOX-II/HIS-/CU2+/MONO/UNIDENTATE/C3P <==
37
DATASET
C 0.426234 1.023289 -0.276937
C 0.172269 2.314867 0.199706
C -1.104292 2.843989 0.175794
C -2.166746 2.102940 -0.326148
C -1.923431 0.817145 -0.800447
C -0.652216 0.281121 -0.769042
O -3.008368 0.100139 -1.276841
O -3.442597 2.574449 -0.384111
C 1.746636 0.411316 -0.271398
C 2.890757 0.995223 0.116317
C 4.180587 0.346206 0.123918
C 5.319719 0.952328 0.523033
C 6.584011 0.243646 0.519335
C 6.528294 -1.092787 0.074859
C 5.346916 -1.707475 -0.336267
O 4.173652 -0.943009 -0.298581
O 7.646217 0.816104 0.897942
H 0.979285 2.919485 0.600793
H -1.297641 3.848113 0.543859
H -0.498945 -0.729979 -1.141817
H 1.781085 -0.619511 -0.620029
H 2.909716 2.026083 0.462860
H -2.706958 -0.587633 -1.890450
H -3.484566 3.480307 -0.047270
H 7.434318 -1.689819 0.045287
O 5.178550 -2.862382 -0.740452
H 5.284141 1.984769 0.854662
CU -4.163545 -0.914126 0.173744
O -5.568291 -2.081355 1.057180
O -2.972929 -2.498660 0.126136
H -2.218847 -2.382485 -0.468517
H -3.453717 -3.272215 -0.200149
H -6.151839 -1.556179 1.621105
H -5.153437 -2.728077 1.644338
O -5.249907 0.704895 0.564071
H -6.073734 0.670672 0.059949
H -4.749099 1.473808 0.217725

ENERGIES
SCF ENERGY: -1303.20154486
SUM OF ELECTRONIC AND ZERO-POINT ENERGIES: -1302.926334
SUM OF ELECTRONIC AND THERMAL ENERGIES: -1302.902893
SUM OF ELECTRONIC AND THERMAL ENTHALPIES: -1302.901949
SUM OF ELECTRONIC AND THERMAL FREE ENERGIES: -1302.979606

==> AOX-II/HIS-/CU2+/MONO/UNIDENTATE/C3P/AEA <==
37
DATASET
C -0.114796 1.110262 0.345121
C 0.305777 2.006900 -0.642601
C 1.603526 2.487033 -0.646501
C 2.508826 2.089884 0.326756
C 2.111014 1.187872 1.315668
C 0.809411 0.716227 1.319869
O 3.041693 0.814405 2.239175
O 3.810470 2.512687 0.344950
C -1.464152 0.568157 0.410981
C -2.491952 0.897578 -0.386806
C -3.817970 0.334987 -0.291752
C -4.843198 0.697945 -1.093077
C -6.153652 0.097090 -0.948603
C -6.268899 -0.868077 0.072403
C -5.202213 -1.239793 0.888801
O -3.973134 -0.602812 0.676870
O -7.107929 0.437890 -1.705512
H -0.377795 2.330760 -1.420789
H 1.936964 3.178905 -1.415822
H 0.511303 0.016363 2.099167
H -1.631252 -0.168499 1.195418
H -2.376969 1.637662 -1.175951
H 2.659398 0.157225 2.835428
H 3.927244 3.238078 -0.284438
H -7.218961 -1.365274 0.240734
O -5.182660 -2.076432 1.797807
H -4.679553 1.450164 -1.857636
CU 3.453641 -0.952786 -0.420725
O 5.090396 -3.351683 -1.203221
O 2.164227 -2.236862 0.227530
H 1.783186 -1.953044 1.069286
H 2.587977 -3.087139 0.405229
H 5.411399 -2.476711 -0.951848
H 4.164287 -3.192813 -1.423705
O 4.768861 0.290257 -1.083428
H 5.658555 -0.056357 -0.940187
H 4.702900 1.105466 -0.548788

ENERGIES
SCF ENERGY: -1303.37937455
SUM OF ELECTRONIC AND ZERO-POINT ENERGIES: -1303.108922
SUM OF ELECTRONIC AND THERMAL ENERGIES: -1303.083130
SUM OF ELECTRONIC AND THERMAL ENTHALPIES: -1303.082186
SUM OF ELECTRONIC AND THERMAL FREE ENERGIES: -1303.167053

==> AOX-II/HIS-/CU2+/MONO/UNIDENTATE/C3P/AIP <==
37
DATASET
C -0.462483 0.969487 0.353002
C -0.241526 2.252992 -0.264337
C 0.995435 2.805124 -0.299607
C 2.080901 2.114472 0.277996
C 1.874686 0.845515 0.923171
C 0.625895 0.292473 0.939310
O 2.970253 0.297096 1.448224
O 3.299117 2.566186 0.261763
C -1.726955 0.370335 0.378897
C -2.878839 0.928384 -0.148326

C -4.125194 0.282849 -0.131374
C -5.241463 0.886725 -0.656226
C -6.534538 0.238410 -0.655188
C -6.513892 -1.049597 -0.082604
C -5.357328 -1.664736 0.456434
O -4.163956 -0.949114 0.416325
O -7.551020 0.796734 -1.130452
H -1.070979 2.786211 -0.713063
H 1.185316 3.765491 -0.767810
H 0.468229 -0.669329 1.419314
H -1.798362 -0.608158 0.847144
H -2.872387 1.909855 -0.611391
H 2.752456 -0.499259 1.960786
H 3.371681 3.434800 -0.174388
H -7.427653 -1.632795 -0.035479
O -5.285889 -2.769979 0.962311
H -5.136672 1.880116 -1.080971
CU 4.231232 -0.918601 -0.179777
O 5.584533 -2.202136 -1.366811
O 2.788739 -2.304541 -0.189445
H 2.307297 -2.388768 0.643268
H 3.178979 -3.172906 -0.353321
H 6.045993 -1.619659 -1.982057
H 5.032381 -2.764713 -1.923064
O 5.493180 0.648846 -0.274744
H 6.209465 0.507484 0.356983
H 5.000695 1.411734 0.063357

ENERGIES
SCF ENERGY: -1302.96159520
SUM OF ELECTRONIC AND ZERO-POINT ENERGIES: -1302.690767
SUM OF ELECTRONIC AND THERMAL ENERGIES: -1302.665065
SUM OF ELECTRONIC AND THERMAL ENTHALPIES: -1302.664121
SUM OF ELECTRONIC AND THERMAL FREE ENERGIES: -1302.748513

==> AOX-II/HIS-/CU2+/MONO/UNIDENTATE/C3P/HAT/C3B <==
36
DATASET
C -0.370977 0.890440 0.260885
C -0.171350 2.270647 -0.112787
C 1.045997 2.882566 -0.062417
C 2.156068 2.141326 0.351914
C 2.006743 0.730823 0.750843
C 0.711517 0.154902 0.665308
O 3.017532 0.113178 1.176282
O 3.361829 2.621303 0.424435
C -1.684094 0.298314 0.231950
C -2.817354 0.922494 -0.147322
C -4.118047 0.309263 -0.148273
C -5.235131 0.958719 -0.545047
C -6.529918 0.302512 -0.522907
C -6.516805 -1.027983 -0.063045
C -5.354288 -1.687723 0.345004
O -4.151095 -0.973853 0.283794
O -7.568122 0.916233 -0.895166
H -1.023683 2.853444 -0.447031
H 1.174809 3.921892 -0.346535
H 0.601377 -0.883587 0.963633
H -1.736865 -0.738828 0.555224
H -2.813047 1.956078 -0.482705
H 3.410556 3.560429 0.170941
H -7.444060 -1.589697 -0.012000
O -5.235042 -2.839820 0.763551
H -5.159070 1.986787 -0.883162
CU 4.165769 -0.897162 -0.118012
O 5.583588 -2.077276 -1.159976
O 2.862910 -2.465593 0.050503
H 2.429598 -2.410844 0.912699
H 3.356145 -3.296324 0.060140
H 5.857418 -1.604555 -1.955773
H 5.166506 -2.887853 -1.477327
O 5.320914 0.728632 -0.567786
H 6.176433 0.596273 -0.139635
H 4.922319 1.494245 -0.125839

ENERGIES
SCF ENERGY: -1302.54710440
SUM OF ELECTRONIC AND ZERO-POINT ENERGIES: -1302.287224
SUM OF ELECTRONIC AND THERMAL ENERGIES: -1302.262527
SUM OF ELECTRONIC AND THERMAL ENTHALPIES: -1302.261583
SUM OF ELECTRONIC AND THERMAL FREE ENERGIES: -1302.342361

==> AOX-II/HIS-/CU2+/MONO/UNIDENTATE/C3P/HAT/C4B <==
36
DATASET
C -0.001160 -1.041246 0.139606
C -0.460995 -1.382933 -1.199082
C -1.707091 -1.823246 -1.414765
C -2.644384 -1.996679 -0.310080
C -2.160688 -1.587838 1.050670
C -0.884512 -1.136320 1.232317
O -3.057412 -1.749112 1.994724
O -3.767452 -2.451411 -0.453412
C 1.314396 -0.612412 0.383977
C 2.290210 -0.519808 -0.575386
C 3.627792 -0.132547 -0.309397
C 4.564981 -0.098762 -1.294356
C 5.942617 0.269222 -1.013561
C 6.192735 0.579719 0.333978
C 5.213234 0.547090 1.342671
O 3.916536 0.179399 0.973572
O 6.802547 0.289016 -1.931534
H 0.220016 -1.273311 -2.034485
H -2.067016 -2.087713 -2.403174
H -0.539403 -0.869850 2.227657
H 1.566810 -0.365512 1.411661
H 2.089381 -0.762769 -1.614578
H -2.711173 -1.500762 2.866955
H 7.193274 0.864284 0.643091
O 5.352545 0.804052 2.531953
H 4.273112 -0.360014 -2.306340
CU -3.355174 0.995389 -0.338216
O -4.794673 3.527075 0.224756
O -1.630080 1.836447 -0.531311
H -1.111576 1.743726 0.279361
H -1.714209 2.787314 -0.680351
H -5.130298 2.810928 -0.327170
H -3.836720 3.420942 0.182056
O -5.083134 0.147624 -0.200870
H -5.575911 0.484379 0.558957
H -4.990044 -0.808104 -0.057975

ENERGIES
SCF ENERGY: -1302.55871564
SUM OF ELECTRONIC AND ZERO-POINT ENERGIES: -1302.297991
SUM OF ELECTRONIC AND THERMAL ENERGIES: -1302.273197
SUM OF ELECTRONIC AND THERMAL ENTHALPIES: -1302.272253
SUM OF ELECTRONIC AND THERMAL FREE ENERGIES: -1302.353468

==> AOX-II/HIS-/CU2+/MONO/UNIDENTATE/C3P/HAT/H2O <==
36
DATASET
C -0.419257 0.823029 0.148759
C -0.138403 2.132724 -0.337401
C 1.128169 2.645981 -0.310425
C 2.181937 1.881904 0.206340
C 1.908002 0.580467 0.700288
C 0.636421 0.058015 0.643813
O 2.961247 -0.097917 1.216444

O	3.431176	2.308486	0.261839
C	-1.738047	0.269773	0.163760
C	-2.876793	0.920456	-0.183797
C	-4.176540	0.329391	-0.131285
C	-5.305132	1.015681	-0.445726
C	-6.610990	0.391104	-0.371161
C	-6.597274	-0.956129	0.041462
C	-5.424805	-1.655631	0.359921
O	-4.213231	-0.965073	0.261538
O	-7.654752	1.035383	-0.660116
H	-0.939257	2.739691	-0.744956
H	1.344722	3.641514	-0.685137
H	0.458723	-0.945646	1.021431
H	-1.813933	-0.759052	0.508535
H	-2.861808	1.955565	-0.513693
H	2.657706	-0.888854	1.698138
H	3.520036	3.212377	-0.083151
H	-7.530140	-1.504238	0.124868
O	-5.315138	-2.823347	0.724056
H	-5.221306	2.052211	-0.754919
CU	4.319554	-0.864627	-0.133233
O	5.495815	-1.542156	-1.420952
O	2.975266	-2.187587	-0.772958
H	2.455903	-2.531840	-0.031960
H	3.465347	-2.942254	-1.129379
H	6.299270	-1.015768	-1.333862
O	5.641415	0.502061	0.504975
H	5.816521	0.287024	1.432429
H	5.103056	1.314057	0.529505

ENERGIES
SCF ENERGY: -1302.49809645
SUM OF ELECTRONIC AND ZERO-POINT ENERGIES: -1302.236677
SUM OF ELECTRONIC AND THERMAL ENERGIES: -1302.213160
SUM OF ELECTRONIC AND THERMAL ENTHALPIES: -1302.212216
SUM OF ELECTRONIC AND THERMAL FREE ENERGIES: -1302.290041

==> AOX-II/HIS-/CU2+/MONO/UNIDENTATE/C3P/RAF/C1B <==
39
DATASET

C	0.021404	-0.737038	-0.023912
C	0.522879	0.256246	-1.018015
C	1.280238	1.307373	-0.678160
C	1.637693	1.510795	0.688675
C	1.238810	0.586579	1.716057
C	0.501346	-0.483391	1.370288
O	1.678813	0.889738	2.953064
O	2.334318	2.524951	1.070483
C	-1.484419	-0.721815	0.067197
C	-2.293709	0.135183	-0.557593
C	-3.737528	0.126487	-0.439795
C	-4.536280	0.981079	-1.107602
C	-5.980565	0.920293	-0.959793
C	-6.454203	-0.077330	-0.086417
C	-5.613314	-0.956103	0.597669
O	-4.233345	-0.818958	0.393085
O	-6.724427	1.722982	-1.589894
H	0.252176	0.074332	-2.054449
H	1.649650	2.021813	-1.407721
H	0.202279	-1.210792	2.121900
H	-1.880134	-1.492646	0.725870
H	-1.904232	0.906884	-1.217959
H	1.372926	0.227716	3.588947
H	2.597525	3.102734	0.329727
H	-7.521455	-0.188039	0.077261
O	-5.916263	-1.860962	1.378769
H	-4.093571	1.720979	-1.765972
CU	4.053138	-0.221650	-0.124360
O	5.870067	-1.252443	-2.275264
O	3.174866	-1.934672	0.139602
H	3.105121	-2.105934	1.089969
H	3.694996	-2.667145	-0.219827
H	5.409572	-0.405099	-2.284441
H	5.367281	-1.775911	-1.639934
O	4.872172	1.502216	-0.439046
H	5.804804	1.388449	-0.665728
H	4.862014	2.037697	0.365524
O	0.389643	-2.044729	-0.442771
H	1.359181	-2.122308	-0.352563

ENERGIES
SCF ENERGY: -1378.98078304
SUM OF ELECTRONIC AND ZERO-POINT ENERGIES: -1378.692691
SUM OF ELECTRONIC AND THERMAL ENERGIES: -1378.666164
SUM OF ELECTRONIC AND THERMAL ENTHALPIES: -1378.665220
SUM OF ELECTRONIC AND THERMAL FREE ENERGIES: -1378.752371

==> AOX-II/HIS-/CU2+/MONO/UNIDENTATE/C3P/RAF/C1P <==
39
DATASET

C	0.180510	-2.011739	-0.157178
C	0.664242	-2.032292	1.188751
C	1.987146	-1.832222	1.438915
C	2.885646	-1.622212	0.378744
C	2.441192	-1.627573	-0.972245
C	1.115642	-1.809451	-1.222720
O	3.390784	-1.430610	-1.908532
O	4.169510	-1.399003	0.570794
C	-1.145578	-2.105276	-0.499844
C	-2.362843	-2.212023	0.331448
C	-2.963209	-0.816951	0.221460
C	-2.697828	0.180119	1.073322
C	-3.278040	1.498554	0.849936
C	-4.093522	1.612084	-0.293190
C	-4.343785	0.555752	-1.168712
O	-3.746263	-0.684117	-0.863270
O	-3.025747	2.448075	1.639280
H	-0.030211	-2.188204	2.002381
H	2.378359	-1.826438	2.451398
H	0.756478	-1.794449	-2.248250
H	-1.382200	-2.010506	-1.559959
H	-3.054083	-2.903670	-0.176240
H	2.996875	-1.446940	-2.791531
H	4.397992	-1.413050	1.515170
H	-4.555868	2.565227	-0.529289
O	-5.027360	0.539282	-2.192012
H	-2.034660	0.014401	1.915910
CU	2.111624	1.407998	-0.037590
O	2.046381	4.398955	0.175160
O	0.214324	1.418594	0.317970
H	-0.285717	0.829898	-0.264133
H	-0.178314	2.294408	0.204435
H	2.661310	3.846800	-0.322892
H	1.626972	3.777348	0.782189
O	4.022582	1.460340	-0.340221
H	4.200344	1.592653	-1.280600
H	4.415688	0.599997	-0.124451
O	-2.111090	-2.600674	1.648710
H	-2.952458	-2.636772	2.120802

ENERGIES
SCF ENERGY: -1379.00611473
SUM OF ELECTRONIC AND ZERO-POINT ENERGIES: -1378.717811
SUM OF ELECTRONIC AND THERMAL ENERGIES: -1378.691019
SUM OF ELECTRONIC AND THERMAL ENTHALPIES: -1378.690075
SUM OF ELECTRONIC AND THERMAL FREE ENERGIES: -1378.776157

==> AOX-II/HIS-/CU2+/MONO/UNIDENTATE/C3P/RAF/C2 <==
39

DATASET

C	0.312999	-1.255792	0.299812
C	0.858296	-1.216541	1.588369
C	2.195761	-1.503235	1.788285
C	3.015715	-1.832864	0.717098
C	2.486431	-1.885444	-0.576608
C	1.147651	-1.603340	-0.771235
O	3.338785	-2.208332	-1.586357
O	4.349125	-2.079706	0.851306
C	-1.071460	-0.934839	0.011830
C	-1.972127	-0.445697	0.884105
C	-3.336574	-0.153040	0.548193
C	-4.211355	0.378045	1.449472
C	-5.586620	0.607094	1.142954
C	-5.938607	0.450683	-0.299961
C	-4.924675	-0.091245	-1.194360
O	-3.684625	-0.480637	-0.719397
O	-6.454824	0.892641	1.967914
H	0.239207	-0.964894	2.443270
H	2.629496	-1.471026	2.784346
H	0.743666	-1.642927	-1.780730
H	-1.383482	-1.102206	-1.017825
H	-1.706793	-0.236430	1.916782
H	2.864024	-2.204926	-2.427718
H	4.597843	-2.063071	1.786198
H	-6.993844	0.368060	-0.552025
O	-5.157175	1.281673	-1.207646
H	-3.859079	0.560880	2.458422
CU	3.427974	1.144293	-0.213128
O	4.586378	3.921684	-0.134083
O	1.689973	1.970445	-0.032619
H	0.993897	1.320441	-0.204011
H	1.566864	2.663819	-0.694050
H	5.041451	3.123530	-0.428146
H	3.791466	3.580746	0.293561
O	5.192840	0.357666	-0.330924
H	5.427397	0.233875	-1.259501
H	5.125768	-0.543048	0.042277
O	-5.146314	-0.808060	-2.319104
H	-6.055043	-0.669018	-2.620569

ENERGIES
SCF ENERGY: -1379.00367758
SUM OF ELECTRONIC AND ZERO-POINT ENERGIES: -1378.716378
SUM OF ELECTRONIC AND THERMAL ENERGIES: -1378.689483
SUM OF ELECTRONIC AND THERMAL ENTHALPIES: -1378.688539
SUM OF ELECTRONIC AND THERMAL FREE ENERGIES: -1378.775408

==> AOX-II/HIS-/CU2+/MONO/UNIDENTATE/C3P/RAF/C2B <==
39
DATASET

C	-0.304636	0.996878	0.191856
C	0.069750	2.056774	-0.596033
C	1.370893	2.576192	-0.552106
C	2.356702	2.116042	0.301703
C	2.028121	1.089518	1.177099
C	0.778598	0.324418	0.964302
O	2.842675	0.714537	2.117351
O	3.605581	2.639836	0.384750
C	-1.609660	0.407448	0.234515
C	-2.698493	0.925863	-0.370270
C	-4.009503	0.339043	-0.321414
C	-5.088359	0.906858	-0.902947
C	-6.396127	0.277714	-0.826898
C	-6.433080	-0.937761	-0.118854
C	-5.307300	-1.516757	0.472897
O	-4.091047	-0.832895	0.353088
O	-7.399615	0.816087	-1.370680
H	-0.642078	2.526752	-1.265141
H	1.619899	3.413712	-1.199702
H	-1.716984	-0.503744	0.816382
H	-2.642036	1.854217	-0.933606
H	2.394995	0.046368	2.676322
H	3.661181	3.466532	-0.116454
H	-7.371213	-1.474335	-0.018960
O	-5.229778	-2.573622	1.100772
H	-4.973213	1.846629	-1.432554
CU	3.759732	-0.871869	-0.402847
O	5.504850	-2.874856	-1.836865
O	2.509532	-2.197238	0.247270
H	2.643820	-2.361814	1.190435
H	2.625140	-3.052425	-0.187529
H	5.737595	-1.976042	-1.574452
H	4.580980	-2.955103	-1.569916
O	4.899491	0.540827	-1.051200
H	5.827576	0.344442	-0.870321
H	4.693380	1.351786	-0.549300
H	1.162943	-0.405607	0.203396
O	0.427579	-0.348767	2.136374
H	0.270198	-1.281818	1.944005

ENERGIES
SCF ENERGY: -1378.99264290
SUM OF ELECTRONIC AND ZERO-POINT ENERGIES: -1378.705095
SUM OF ELECTRONIC AND THERMAL ENERGIES: -1378.678376
SUM OF ELECTRONIC AND THERMAL ENTHALPIES: -1378.677432
SUM OF ELECTRONIC AND THERMAL FREE ENERGIES: -1378.763192

==> AOX-II/HIS-/CU2+/MONO/UNIDENTATE/C3P/RAF/C2P <==
39
DATASET

C	0.233528	0.764153	-0.533532
C	-0.251627	0.661337	-1.846006
C	-1.018508	-0.428321	-2.207078
C	-1.281493	-1.432485	-1.284911
C	-0.766019	-1.351092	0.027739
C	-0.008302	-0.262803	0.391154
O	-1.085452	-2.362499	0.864743
O	-2.045956	-2.505444	-1.561560
C	1.049885	1.971854	-0.124784
C	2.226084	1.435772	-0.809736
C	3.224209	0.637104	-0.232631
C	4.285939	0.213931	-0.974298
C	5.377621	-0.534347	-0.367962
C	5.196586	-0.799263	0.997992
C	4.082187	-0.373909	1.747699
O	3.101925	0.368374	1.086059
O	6.364576	-0.893070	-1.058458
H	-0.048640	1.448142	-2.564230
H	-1.427612	-0.518989	-3.208975
H	0.368481	-0.190751	1.407925
H	2.322533	1.655429	-1.871606
H	-0.688997	-2.211192	1.733263
H	-2.326422	-2.494860	-2.489005
H	5.948981	-1.364026	1.538830
O	3.861335	-0.571187	2.934200
H	4.316413	0.452685	-2.032497
CU	-3.552319	0.172837	0.094232
O	-5.713021	0.867965	2.075544
O	-2.937071	2.001456	0.221500
H	-1.980377	2.077310	0.097140
H	-3.110919	2.343583	1.108477
H	-5.182097	0.063305	2.034457
H	-5.518951	1.307132	1.238546
O	-4.325860	-1.597795	-0.038300
H	-4.425579	-1.974734	0.845836
H	-3.678280	-2.165372	-0.494202
H	1.156464	2.014002	0.962796
O	0.534334	3.150298	-0.678945
H	1.153222	3.869712	-0.499637

ENERGIES
SCF ENERGY: -1378.99497468
SUM OF ELECTRONIC AND ZERO-POINT ENERGIES: -1378.708683
SUM OF ELECTRONIC AND THERMAL ENERGIES: -1378.681227
SUM OF ELECTRONIC AND THERMAL ENTHALPIES: -1378.680283
SUM OF ELECTRONIC AND THERMAL FREE ENERGIES: -1378.768734

==> AOX-II/HIS-/CU2+/MONO/UNIDENTATE/C3P/RAF/C3 <==
39
DATASET

C	0.530282	-0.809726	-0.709662
C	1.067929	-1.914961	-0.039904
C	2.413280	-2.215798	-0.156749
C	3.246092	-1.429878	-0.942112
C	2.724048	-0.327754	-1.629533
C	1.380242	-0.028941	-1.506238
O	3.590335	0.401503	-2.381258
O	4.581431	-1.655273	-1.068118
C	-0.861624	-0.421681	-0.619549
C	-1.823569	-1.045543	0.088039
C	-3.190376	-0.624428	0.112782
C	-4.162555	-1.251126	0.819303
C	-5.522905	-0.804417	0.781944
C	-5.827542	0.153940	-0.356342
C	-4.677405	1.072330	-0.668704
O	-3.435926	0.516023	-0.618909
O	-6.409944	-1.187171	1.530099
H	0.437286	-2.545908	0.578268
H	2.841566	-3.067676	0.364718
H	0.981394	0.830435	-2.042785
H	-1.134111	0.461576	-1.194564
H	-1.614412	-1.934262	0.677462
H	3.125318	1.140144	-2.796383
H	4.829962	-2.453678	-0.582131
H	-5.883415	-0.505840	-1.245742
O	-4.788556	2.207796	-1.025159
H	-3.894635	-2.091497	1.449299
CU	3.664106	0.598095	1.354936
O	2.498943	2.948395	-0.099393
O	1.932373	0.590444	2.234553
H	1.488181	-0.249673	2.050056
H	1.375890	1.264974	1.821240
H	2.790970	2.947266	0.820513
H	2.371688	2.009683	-0.290853
O	5.390953	0.610532	0.470612
H	5.447156	1.416168	-0.060156
H	5.361412	-0.122710	-0.173383
O	-7.019385	0.837948	-0.136892
H	-7.280487	1.282569	-0.952653

ENERGIES
SCF ENERGY: -1379.04718566
SUM OF ELECTRONIC AND ZERO-POINT ENERGIES: -1378.758623
SUM OF ELECTRONIC AND THERMAL ENERGIES: -1378.732154
SUM OF ELECTRONIC AND THERMAL ENTHALPIES: -1378.731210
SUM OF ELECTRONIC AND THERMAL FREE ENERGIES: -1378.815214

==> AOX-II/HIS-/CU2+/MONO/UNIDENTATE/C3P/RAF/C3B <==
39
DATASET

C	0.110946	-1.198500	0.301044
C	0.718576	-0.691133	1.513430
C	2.038402	-0.829534	1.824292
C	2.871950	-1.512120	0.939331
C	2.347533	-2.108969	-0.359202
C	0.885838	-1.880193	-0.565670
O	3.101975	-1.472630	-1.353198
O	4.120343	-1.728752	1.132903
C	-1.297014	-0.968114	0.013222
C	-2.144209	-0.262647	0.774863
C	-3.536552	-0.039323	0.458186
C	-4.365247	0.679141	1.242875
C	-5.756335	0.875272	0.876740
C	-6.152194	0.267970	-0.330636
C	-5.283916	-0.473809	-1.132121
O	-3.958265	-0.607114	-0.697308
O	-6.526270	1.550710	1.616068
H	0.090668	-0.164159	2.226510
H	2.455718	-0.434941	2.744885
H	0.485101	-2.293146	-1.489015
H	-1.656582	-1.412665	-0.912182
H	-1.826434	0.201047	1.705415
H	2.715831	-1.669306	-2.219799
H	4.449848	-1.355284	1.972889
H	-7.177053	0.367682	-0.673837
O	-5.520280	-1.046255	-2.199015
H	-3.984501	1.116354	2.159807
CU	2.797040	1.395363	-0.324246
O	3.045720	4.391461	-0.646515
O	1.078043	1.614313	-1.191938
H	0.835180	0.840398	-1.717851
H	1.112862	2.352400	-1.814987
H	3.696126	3.707301	-0.846496
H	2.276032	3.891631	-0.348461
O	4.527084	1.324239	0.545962
H	5.051340	2.103551	0.318224
H	5.033118	0.567950	0.219759
O	2.534967	-3.493872	-0.338875
H	3.475404	-3.689375	-0.204530

ENERGIES
SCF ENERGY: -1378.98300868
SUM OF ELECTRONIC AND ZERO-POINT ENERGIES: -1378.695127
SUM OF ELECTRONIC AND THERMAL ENERGIES: -1378.668103
SUM OF ELECTRONIC AND THERMAL ENTHALPIES: -1378.667159
SUM OF ELECTRONIC AND THERMAL FREE ENERGIES: -1378.754534

==> AOX-II/HIS-/CU2+/MONO/UNIDENTATE/C3P/RAF/C4 <==
39
DATASET

C	-0.304773	1.115409	-0.263662
C	-0.761662	1.754292	0.894134
C	-2.071609	2.189369	0.984715
C	-2.953248	2.000842	-0.070003
C	-2.517936	1.357618	-1.231369
C	-1.205666	0.926220	-1.319366
O	-3.428947	1.184145	-2.228269
O	-4.262869	2.388154	-0.024725
C	1.061228	0.636566	-0.422144
C	2.045199	0.750545	0.482984
C	3.396450	0.290449	0.294967
C	4.373817	0.421992	1.199927
C	5.736393	0.008291	0.870741
C	5.942763	-0.699450	-0.399570
C	4.760292	-0.954160	-1.261296
O	3.610411	-0.319735	-0.936426
O	6.311129	0.672784	-0.249673
H	-0.094675	1.916836	1.734390
H	-2.432811	2.684789	1.882347
H	-0.878939	0.429267	-2.230924
H	1.278657	0.155042	-1.374040
H	1.876371	1.230328	1.444491
H	-3.014355	0.723633	-2.969136
H	-4.430103	2.894047	0.782510
H	6.764259	-1.400939	-0.523909
O	4.769162	-1.684446	-2.216086
H	4.165605	0.874869	2.161902
CU	-3.606308	-0.987744	0.591672

O -4.771572 -3.701635 0.025711
O -1.882852 -1.836372 0.800526
H -1.188030 -1.261742 0.446291
H -1.851219 -2.640801 0.265515
H -5.169513 -2.845874 -0.174906
H -4.079701 -3.482190 0.661417
O -5.347903 -0.163151 0.441012
H -5.836004 -0.549549 -0.297182
H -5.194062 0.771611 0.198902
O 6.537451 -0.176256 1.960587
H 7.387884 -0.550572 1.692228

ENERGIES
SCF ENERGY: -1379.00419417
SUM OF ELECTRONIC AND ZERO-POINT ENERGIES: -1378.715594
SUM OF ELECTRONIC AND THERMAL ENERGIES: -1378.689214
SUM OF ELECTRONIC AND THERMAL ENTHALPIES: -1378.688270
SUM OF ELECTRONIC AND THERMAL FREE ENERGIES: -1378.773372

==> AOX-II/HIS-/CU2+/MONO/UNIDENTATE/C3P/RAF/C4B <==
39
DATASET
C -0.764789 -1.277560 0.156564
C -1.283266 -1.664744 -1.139004
C -2.590912 -1.847194 -1.322428
C -3.602690 -1.646865 -0.239479
C -2.987103 -1.242548 1.082085
C -1.641299 -1.083440 1.237910
O -3.880337 -1.071667 2.019277
O -4.465048 -0.585143 -0.587731
C 0.618816 -1.070095 0.385751
C 1.577470 -1.108235 -0.576713
C 2.953744 -0.809961 -0.335623
C 3.880379 -0.845633 -1.321375
C 5.275430 -0.533168 -1.050251
C 5.552554 -0.203623 0.287274
C 4.581359 -0.169156 1.297468
O 3.265713 -0.481467 0.939288
O 6.130972 -0.568281 -1.974271
H -0.596353 -1.819337 -1.962478
H -3.003406 -2.143290 -2.284998
H -1.245770 -0.793740 2.207766
H 0.907131 -0.822260 1.404458
H 1.342508 -1.351357 -1.609090
H -3.474318 -0.824973 2.867613
H -4.855896 -0.764952 -1.457240
H 6.567954 0.042439 0.580807
O 4.731944 0.104378 2.485356
H 3.573925 -1.112573 -2.327303
CU -1.219232 1.794241 -0.158585
O -1.556873 4.785541 -0.379297
O 0.504551 2.080535 0.679378
H 0.637289 1.500692 1.441135
H 0.551747 2.981178 1.027322
H -1.595733 4.141391 -1.096678
H -1.430522 4.239708 0.406473
O -2.926762 1.749562 -1.058234
H -3.438819 2.531470 -0.817111
H -3.493434 0.979871 -0.850609
O -4.304647 -2.829435 0.015686
H -4.502836 -3.276130 -0.819784

ENERGIES
SCF ENERGY: -1378.99971154
SUM OF ELECTRONIC AND ZERO-POINT ENERGIES: -1378.712196
SUM OF ELECTRONIC AND THERMAL ENERGIES: -1378.686047
SUM OF ELECTRONIC AND THERMAL ENTHALPIES: -1378.685103
SUM OF ELECTRONIC AND THERMAL FREE ENERGIES: -1378.769938

==> AOX-II/HIS-/CU2+/MONO/UNIDENTATE/C3P/RAF/C5 <==
39
DATASET
C 0.381055 -0.707612 -1.042057
C 0.791841 -1.944639 -0.506223
C 2.123997 -2.279088 -0.485669
C 3.079513 -1.397651 -0.994117
C 2.685885 -0.171061 -1.559915
C 1.353911 0.162396 -1.577041
O 3.673507 0.633868 -2.029210
O 4.396495 -1.653373 -0.959400
C -0.964142 -0.264737 -1.011967
C -2.043235 -0.945117 -0.493838
C -3.312657 -0.386673 -0.482995
C -4.497032 -1.098616 0.041755
C -5.430157 -0.158912 0.816452
C -5.521413 1.142564 0.327489
C -4.666624 1.635111 -0.654977
O -3.516415 0.796854 -1.004538
O -6.059691 -0.668643 1.756342
H 0.063320 -2.635783 -0.096988
H 2.461577 -3.222891 -0.067298
H 1.051183 1.120792 -1.993556
H -1.147764 0.724386 -1.430666
H -1.946239 -1.937238 -0.069499
H 3.289215 1.382577 -2.506822
H 4.564012 -2.529702 -0.581679
H -6.266629 1.826753 0.717406
O -4.655614 2.688089 -1.253112
H -5.079076 -1.361501 -0.866431
CU 3.134737 0.439084 1.522161
O 3.299651 2.953617 -0.106557
O 1.256274 0.775184 1.872043
H 0.760288 -0.054765 1.848105
H 0.894879 1.304563 1.147416
H 3.633025 2.083345 -0.367409
H 3.218445 2.893529 0.852838
O 5.030943 0.183229 1.211090
H 5.420466 1.031769 0.960919
H 5.122880 -0.385197 0.426559
O -4.176221 -2.238019 0.778091
H -4.824039 -2.256314 1.505070

ENERGIES
SCF ENERGY: -1379.02696704
SUM OF ELECTRONIC AND ZERO-POINT ENERGIES: -1378.739579
SUM OF ELECTRONIC AND THERMAL ENERGIES: -1378.712958
SUM OF ELECTRONIC AND THERMAL ENTHALPIES: -1378.712014
SUM OF ELECTRONIC AND THERMAL FREE ENERGIES: -1378.797649

==> AOX-II/HIS-/CU2+/MONO/UNIDENTATE/C3P/RAF/C5B <==
39
DATASET
C 0.120607 -1.266692 0.062837
C 0.608305 -1.328241 1.327838
C 2.059472 -1.377439 1.551048
C 2.895110 -1.772771 0.407594
C 2.370086 -1.666093 -0.892343
C 1.027637 -1.439232 -1.034309
O 3.251959 -1.821279 -1.902240
O 4.124292 -2.114855 0.587187
C -1.286195 -1.017312 -0.244516
C -2.112688 -0.345724 0.566624
C -3.509737 -0.088681 0.300658
C -4.308407 0.596142 1.144098
C -5.707496 0.821180 0.828953
C -6.141998 0.285141 -0.398732
C -5.303484 -0.418855 -1.263410
O -3.968910 -0.591261 -0.871066
O -6.451293 1.460351 1.625407

H -0.020352 -1.232102 2.207711
H 2.305457 -0.270493 1.515136
H 0.626468 -1.395405 -2.045547
H -1.642533 -1.380708 -1.206073
H -1.748626 0.068972 1.505049
H 2.801877 -1.823816 -2.759335
H 4.311422 -2.210367 1.543915
H -7.174661 0.413098 -0.707479
O -5.574038 -0.926867 -2.354546
H -3.898093 0.980031 2.072100
CU 2.674638 1.375659 -0.334450
O 3.582194 4.242954 -0.031208
O 1.030288 2.007128 0.470788
H 0.254005 1.581582 0.082352
H 0.923859 2.951090 0.291082
H 3.617830 3.672250 -0.808454
H 3.187888 3.680178 0.646008
O 4.368791 0.827787 -1.107476
H 4.686896 1.497573 -1.726427
H 4.261822 0.013929 -1.627492
O 2.540952 -1.957324 2.723011
H 2.441081 -1.341527 3.459610

ENERGIES
SCF ENERGY: -1378.98619344
SUM OF ELECTRONIC AND ZERO-POINT ENERGIES: -1378.700919
SUM OF ELECTRONIC AND THERMAL ENERGIES: -1378.674061
SUM OF ELECTRONIC AND THERMAL ENTHALPIES: -1378.673117
SUM OF ELECTRONIC AND THERMAL FREE ENERGIES: -1378.759357

==> AOX-II/HIS-/CU2+/MONO/UNIDENTATE/C3P/RAF/C6 <==
39
DATASET
C -0.184540 -1.094564 -0.004682
C -0.634530 -1.275664 -1.326876
C -1.919604 -1.699012 -1.564372
C -2.786474 -1.952606 -0.501545
C -2.353757 -1.789736 0.827785
C -1.067913 -1.364750 1.061806
O -3.256660 -2.058046 1.801727
O -4.062543 -2.334770 -0.678974
C 1.117441 -0.629936 0.309263
C 2.110242 -0.298283 -0.583772
C 3.353200 0.148189 -0.153705
C 4.439670 0.510461 -1.105797
C 5.833377 0.150506 -0.566033
C 5.976787 0.232685 0.824145
C 4.915071 0.417215 1.698625
O 3.571887 0.317986 1.122774
O 6.734669 -0.091037 -1.378021
H 0.023325 -1.079210 -2.166063
H -2.287716 -1.837149 -2.576798
H -0.730620 -1.229780 2.086930
H 1.342461 -0.532071 1.370721
H 1.964359 -0.380216 -1.654044
H -2.861478 -1.899343 2.669235
H -4.257920 -2.456949 -1.620419
H 6.963920 0.151744 1.266089
O 4.878316 0.596403 2.897642
H 4.446906 1.617423 -1.107005
CU -3.503094 1.052887 0.115240
O -4.679764 3.713814 -0.629415
O -1.789389 1.945801 0.039499
H -1.186949 1.617541 0.720549
H -1.898731 2.889339 0.217371
H -5.121491 3.007694 -0.142359
H -3.930069 3.267626 -1.041575
O -5.230528 0.174598 0.110757
H -5.612956 0.191835 0.997386
H -5.081844 -0.767628 -0.088693
O 4.247535 0.004510 -2.393121
H 3.716525 0.624332 -2.905267

ENERGIES
SCF ENERGY: -1379.02011958
SUM OF ELECTRONIC AND ZERO-POINT ENERGIES: -1378.733037
SUM OF ELECTRONIC AND THERMAL ENERGIES: -1378.705844
SUM OF ELECTRONIC AND THERMAL ENTHALPIES: -1378.704900
SUM OF ELECTRONIC AND THERMAL FREE ENERGIES: -1378.792898

==> AOX-II/HIS-/CU2+/MONO/UNIDENTATE/C3P/RAF/C6B <==
39
DATASET
C -0.056679 -1.099470 0.376946
C -0.518755 -1.678997 -0.926918
C -1.932232 -2.130819 -0.963380
C -2.789342 -1.910104 0.041631
C -2.300170 -1.298113 1.253634
C -0.959724 -0.921180 1.393003
O -3.177835 -1.138162 2.200827
O -4.109466 -2.206602 0.051687
C 1.290830 -0.666596 0.562573
C 2.212445 -0.569544 -0.421451
C 3.559211 -0.112207 -0.225863
C 4.442676 -0.002374 -1.241820
C 5.804899 0.446631 -1.002941
C 6.101970 0.753077 0.337107
C 5.176305 0.634741 1.378950
O 3.888864 0.192293 1.051738
O 6.627248 0.535881 -1.955176
H -0.472529 -0.824078 -1.635577
H -2.266329 -2.616971 -1.876496
H -0.652484 -0.473365 2.334566
H 1.553374 -0.358322 1.571663
H 1.966641 -0.827257 -1.447484
H -2.789217 -0.730158 2.993300
H -4.355188 -2.672923 -0.760570
H 7.097322 1.094234 0.603152
O 5.339385 0.870514 2.575890
H 4.129612 -0.261061 -2.247816
CU -3.139737 1.202074 -0.391828
O -4.290669 3.992879 -0.272110
O -1.320298 1.810510 -0.650497
H -0.826765 1.780232 0.180151
H -1.288310 2.733402 -0.935379
H -4.793078 3.169708 -0.260713
H -3.407287 3.714404 -0.542095
O -4.949624 0.532183 -0.200073
H -5.358030 0.924244 0.582459
H -4.910290 -0.426727 -0.027126
O 0.368309 -2.700118 -1.335133
H 0.229031 -2.870779 -2.275078

ENERGIES
SCF ENERGY: -1378.99863736
SUM OF ELECTRONIC AND ZERO-POINT ENERGIES: -1378.710401
SUM OF ELECTRONIC AND THERMAL ENERGIES: -1378.683590
SUM OF ELECTRONIC AND THERMAL ENTHALPIES: -1378.682646
SUM OF ELECTRONIC AND THERMAL FREE ENERGIES: -1378.768997

==> AOX-II/HIS-/CU2+/MONO/UNIDENTATE/C4P <==
37
DATASET
C -0.633003 0.662379 -0.289554
C 0.151921 -0.436853 -0.660703
C 1.527422 -0.318308 -0.746302
C 2.134375 0.893262 -0.467556
C 1.377772 2.001698 -0.100555
C 0.002213 1.879219 -0.018896

O 2.063308 3.146792 0.160626
O 3.505525 0.989842 -0.549225
C -2.081742 0.603621 -0.153055
C -2.855066 -0.479925 -0.319212
C -4.289447 -0.494779 -0.152426
C -5.048109 -1.600064 -0.314374
C -6.484663 -1.555078 -0.125792
C -7.009541 -0.297267 0.233627
C -6.216426 0.837156 0.398779
O -4.838045 0.697639 0.191052
O -7.187647 -2.594549 -0.281825
H -0.309322 -1.394493 -0.878516
H 2.153855 -1.162257 -1.023113
H -0.585466 2.747603 0.271054
H -2.558236 1.543121 0.122439
H -2.428635 -1.443340 -0.590006
H 1.457555 3.853161 0.420752
H 3.805618 1.858605 -0.237458
H -8.076828 -0.183459 0.395225
O -6.561847 1.980994 0.711154
H -4.572104 -2.535819 -0.587982
CU 4.722041 -0.559617 0.155125
O 6.137620 -1.750554 0.973612
O 3.659921 -0.898582 1.813916
H 2.932149 -0.268145 1.910807
H 4.217965 -0.775981 2.594621
H 6.747230 -2.115439 0.318714
H 5.730830 -2.513862 1.405805
O 5.586528 -0.463801 -1.617501
H 6.463473 -0.869642 -1.648188
H 5.705599 0.456226 -1.891136

ENERGIES
SCF ENERGY: -1303.19842855
SUM OF ELECTRONIC AND ZERO-POINT ENERGIES: -1302.925371
SUM OF ELECTRONIC AND THERMAL ENERGIES: -1302.900961
SUM OF ELECTRONIC AND THERMAL ENTHALPIES: -1302.900017
SUM OF ELECTRONIC AND THERMAL FREE ENERGIES: -1302.980816

==> AOX-II/HIS-/CU2+/MONO/UNIDENTATE/C4P/AEA <==
37
DATASET
C 0.189340 0.894740 -0.735159
C 0.792726 -0.007633 -1.618157
C 2.154983 0.044798 -1.853265
C 2.940437 0.995333 -1.217658
C 2.347179 1.940716 -0.380510
C 0.985910 1.879965 -0.138488
O 3.179999 2.873011 0.160705
O 4.293401 0.969977 -1.409079
C -1.223550 0.843405 -0.389599
C -2.080846 -0.134483 -0.722334
C -3.479442 -0.160504 -0.365755
C -4.319890 -1.159818 -0.711991
C -5.716630 -1.128447 -0.327709
C -6.114845 -0.000922 0.419057
C -5.238683 1.023931 0.771927
O -3.906510 0.907509 0.354156
O -6.498315 -2.067062 -0.655875
H 0.199577 -0.765253 -2.120387
H 2.638652 -0.663040 -2.520436
H 0.537820 2.607976 0.534885
H -1.588537 1.676627 0.209114
H -1.753675 -0.995359 -1.301926
H 2.691201 3.468041 0.743673
H 4.707319 1.697410 -0.921277
H -7.145886 0.094068 0.744817
O -5.472019 2.050165 1.418558
H -3.940576 -1.996647 -1.289093
CU 2.952967 -1.632122 0.570832
O 3.925999 0.545238 2.385882
O 1.205046 -1.595577 1.427036
H 0.525465 -1.823978 0.777464
H 1.003268 -0.683372 1.679269
H 4.456665 -0.027153 1.817988
H 3.050678 0.507856 1.979620
O 4.692297 -1.621815 -0.297458
H 5.403931 -1.710254 0.349430
H 4.798753 -0.733432 -0.690818

ENERGIES
SCF ENERGY: -1303.38458976
SUM OF ELECTRONIC AND ZERO-POINT ENERGIES: -1303.113643
SUM OF ELECTRONIC AND THERMAL ENERGIES: -1303.089211
SUM OF ELECTRONIC AND THERMAL ENTHALPIES: -1303.088267
SUM OF ELECTRONIC AND THERMAL FREE ENERGIES: -1303.168488

==> AOX-II/HIS-/CU2+/MONO/UNIDENTATE/C4P/AIP <==
37
DATASET
C -0.597559 0.612253 -0.571241
C 0.076391 -0.573892 -1.037187
C 1.405902 -0.555328 -1.294098
C 2.126778 0.641287 -1.106942
C 1.479845 1.834247 -0.637597
C 0.141264 1.807633 -0.386505
O 2.300018 2.873744 -0.496429
O 3.410118 0.633581 -1.333706
C -1.964389 0.630719 -0.278358
C -2.816278 -0.454746 -0.404129
C -4.183927 -0.407981 -0.094057
C -4.976709 -1.519161 -0.245511
C -6.389933 -1.500152 0.065651
C -6.841726 -0.250649 0.533582
C -6.016709 0.890812 0.685709
O -4.670285 0.767711 0.353255
O -7.106803 -2.517893 -0.081234
H -0.477994 -1.492844 -1.181249
H 1.947452 -1.429789 -1.637807
H -0.363765 2.702795 -0.035994
H -2.379538 1.570521 0.077021
H -2.459041 -1.416405 -0.757963
H 1.835794 3.664170 -0.182639
H 3.810563 1.514013 -1.181888
H -7.885288 -0.120373 0.800744
O -6.357413 1.990750 1.081546
H -4.519292 -2.433174 -0.610926
CU 4.620711 -0.488382 0.389256
O 5.713776 -1.570347 1.938966
O 3.935939 0.905703 1.640542
H 3.848942 1.761191 1.198303
H 4.571140 1.045531 2.354785
H 5.720125 -2.501635 1.685961
H 5.187455 -1.534508 2.746898
O 4.975306 -1.870203 -1.008834
H 5.799001 -2.336638 -0.816051
H 5.100854 -1.464417 -1.876342

ENERGIES
SCF ENERGY: -1302.96495516
SUM OF ELECTRONIC AND ZERO-POINT ENERGIES: -1302.693078
SUM OF ELECTRONIC AND THERMAL ENERGIES: -1302.667619
SUM OF ELECTRONIC AND THERMAL ENTHALPIES: -1302.666675
SUM OF ELECTRONIC AND THERMAL FREE ENERGIES: -1302.749384

==> AOX-II/HIS-/CU2+/MONO/UNIDENTATE/C4P/HAT/C3B <==
36
DATASET
C -0.561983 0.687306 -0.613467

C 0.107030 -0.514652 -1.117283
C 1.431909 -0.556339 -1.396226
C 2.171977 0.617829 -1.195913
C 1.546642 1.882563 -0.676619
C 0.151837 1.839305 -0.414488
O 2.310333 2.835878 -0.511354
O 3.434850 0.653565 -1.413571
C -1.959427 0.657550 -0.304260
C -2.782362 -0.404527 -0.473304
C -4.174825 -0.405245 -0.135264
C -4.966164 -1.485092 -0.334011
C -6.375194 -1.457770 0.018306
C -6.820219 -0.246147 0.577707
C -5.989762 0.862855 0.780581
O -4.647948 0.744018 0.399833
O -7.106033 -2.465775 -0.179980
H -0.479509 -1.413908 -1.267998
H 1.926536 -1.448517 -1.763099
H -0.327939 2.734568 -0.032658
H -2.369647 1.578452 0.103405
H -2.428617 -1.343650 -0.888708
H 3.764067 1.563332 -1.207190
H -7.858477 -0.139122 0.874888
O -6.284143 1.956378 1.258895
H -4.536991 -2.383082 -0.765692
CU 4.645677 -0.443936 0.428573
O 5.449811 -1.647828 2.112656
O 3.880364 0.975424 1.608060
H 3.810183 1.832378 1.166196
H 4.464392 1.120036 2.363316
H 5.480636 -2.558604 1.796410
H 4.790070 -1.653040 2.816309
O 5.103492 -1.819912 -0.957328
H 5.869028 -2.334053 -0.670544
H 5.369066 -1.400453 -1.785490

ENERGIES
SCF ENERGY: -1302.54002462
SUM OF ELECTRONIC AND ZERO-POINT ENERGIES: -1302.283094
SUM OF ELECTRONIC AND THERMAL ENERGIES: -1302.258316
SUM OF ELECTRONIC AND THERMAL ENTHALPIES: -1302.257372
SUM OF ELECTRONIC AND THERMAL FREE ENERGIES: -1302.340917

==> AOX-II/HIS-/CU2+/MONO/UNIDENTATE/C4P/HAT/C4B <==
36
DATASET
C -0.622456 0.267899 -0.517264
C -0.017062 -1.019060 -0.751995
C 1.309688 -1.126765 -0.962916
C 2.159110 0.038587 -0.981168
C 1.527503 1.336485 -0.722086
C 0.179909 1.423981 -0.513396
O 2.352801 2.373636 -0.718668
O 3.381647 -0.029584 -1.225677
C -2.009912 0.423955 -0.281829
C -2.915902 -0.594195 -0.236787
C -4.306938 -0.406027 0.006385
C -5.181444 -1.443526 0.029673
C -6.597643 -1.227146 0.273879
C -6.955611 0.116136 0.485073
C -6.041287 1.179699 0.458888
O -4.700416 0.872153 0.209140
O -7.404230 -2.194265 0.286831
H -0.637349 -1.907953 -0.762322
H 1.785665 -2.084520 -1.147618
H -0.275868 2.394083 -0.333600
H -2.362000 1.440164 -0.124808
H -2.617405 -1.627583 -0.386835
H 1.875641 3.201325 -0.546708
H -7.992375 0.372068 0.678158
O -6.264886 2.374148 0.629886
H -4.811737 -2.449477 -0.138692
CU 4.714058 -0.288392 0.279989
O 6.520833 -0.559020 1.363133
O 4.639306 1.674206 0.871728
H 4.022784 2.129572 0.274609
H 5.504234 2.072399 0.713547
H 6.492064 -1.404057 1.828585
H 6.572157 0.109275 2.057680
O 4.384213 -2.310802 0.150761
H 5.218615 -2.786246 0.253320
H 4.076027 -2.519597 -0.740816

ENERGIES
SCF ENERGY: -1302.55867745
SUM OF ELECTRONIC AND ZERO-POINT ENERGIES: -1302.299511
SUM OF ELECTRONIC AND THERMAL ENERGIES: -1302.274486
SUM OF ELECTRONIC AND THERMAL ENTHALPIES: -1302.273542
SUM OF ELECTRONIC AND THERMAL FREE ENERGIES: -1302.355218

==> AOX-II/HIS-/CU2+/MONO/UNIDENTATE/C4P/HAT/H2O <==
36
DATASET
C -0.554792 0.448703 -0.403239
C 0.124779 -0.776333 -0.673895
C 1.465547 -0.785284 -0.919737
C 2.172447 0.423080 -0.903254
C 1.529649 1.648457 -0.587740
C 0.177351 1.650430 -0.363322
O 2.331160 2.718163 -0.560605
O 3.478010 0.407928 -1.177824
C -1.958174 0.514145 -0.184525
C -2.805450 -0.554481 -0.176526
C -4.211788 -0.449817 0.008447
C -5.024302 -1.541439 -0.000824
C -6.458415 -1.416339 0.173172
C -6.910506 -0.093365 0.343325
C -6.061382 1.025983 0.353002
O -4.693361 0.801421 0.181472
O -7.203762 -2.430163 0.164790
H -0.425227 -1.709145 -0.700596
H 2.006886 -1.698274 -1.143631
H -0.329685 2.587344 -0.150642
H -2.373265 1.506077 -0.021754
H -2.441808 -1.567829 -0.320251
H 1.837015 3.529005 -0.369919
H 3.829889 1.318985 -1.240244
H -7.969559 0.101070 0.477840
O -6.370383 2.203296 0.496605
H -4.582505 -2.522037 -0.144238
CU 4.682720 -0.435770 0.350197
O 5.685933 -1.419912 1.603273
O 4.218535 1.084440 1.618577
H 4.014241 1.873552 1.094627
H 5.010353 1.307551 2.127873
H 5.585885 -0.985795 2.457764
O 5.053335 -1.851634 -1.027138
H 5.837851 -2.338892 -0.739228
H 5.295539 -1.435050 -1.865807

ENERGIES
SCF ENERGY: -1302.50054963
SUM OF ELECTRONIC AND ZERO-POINT ENERGIES: -1302.239417
SUM OF ELECTRONIC AND THERMAL ENERGIES: -1302.215578
SUM OF ELECTRONIC AND THERMAL ENTHALPIES: -1302.214634
SUM OF ELECTRONIC AND THERMAL FREE ENERGIES: -1302.293053

==> AOX-II/HIS-/CU2+/MONO/UNIDENTATE/C4P/RAF/C1B <==
39

DATASET
C -0.016066 -0.746144 -0.463297
C 0.352108 0.326941 -1.430425
C 1.035675 1.422922 -1.076980
C 1.492311 1.557307 0.266087
C 1.275862 0.524080 1.240361
C 0.599476 -0.579595 0.890044
O 1.836307 0.796593 2.440790
O 2.105956 2.640940 0.594294
C -1.506807 -0.763664 -0.199288
C -2.399858 0.122130 -0.644403
C -3.816339 0.067745 -0.345582
C -4.704266 0.962628 -0.820018
C -6.114742 0.862043 -0.484983
C -6.458334 -0.217868 0.350505
C -5.526679 -1.138856 0.831440
O -4.188344 -0.958699 0.455450
O -6.942354 1.703906 -0.932753
H 0.012425 0.183327 -2.452523
H 1.276436 2.218092 -1.773360
H 0.430358 -1.381361 1.605728
H -1.811275 -1.594984 0.436074
H -2.107107 0.958963 -1.274818
H 1.551498 0.166365 3.118848
H 2.370258 2.633961 1.536627
H -7.492331 -0.362079 0.647300
O -5.715713 -2.115240 1.560435
H -4.359034 1.767755 -1.459963
CU 4.068708 -0.029364 -0.225699
O 6.607721 -1.679218 -0.171801
O 4.540872 0.431882 1.592102
H 3.728058 0.585608 2.106689
H 5.019302 1.270796 1.606197
H 5.978675 -1.591782 -0.897704
H 6.170205 -1.230470 0.561632
O 3.631538 -0.570928 -2.027152
H 4.415494 -0.855361 -2.515380
H 3.253468 0.155711 -2.539814
O 0.398495 -1.970573 -1.054784
H 0.101180 -2.700318 -0.493682

ENERGIES
SCF ENERGY: -1378.97994948
SUM OF ELECTRONIC AND ZERO-POINT ENERGIES: -1378.692389
SUM OF ELECTRONIC AND THERMAL ENERGIES: -1378.665741
SUM OF ELECTRONIC AND THERMAL ENTHALPIES: -1378.664797
SUM OF ELECTRONIC AND THERMAL FREE ENERGIES: -1378.749779

==> AOX-II/HIS-/CU2+/MONO/UNIDENTATE/C4P/RAF/C1P <==
39
DATASET
C 1.030276 1.866800 0.142085
C 1.421944 1.495764 -1.186219
C 2.570912 0.797756 -1.389555
C 3.375467 0.435930 -0.294668
C 3.019030 0.799461 1.032568
C 1.880402 1.509756 1.239926
O 3.880974 0.373577 1.980426
O 4.466735 -0.256045 -0.523449
C -0.164440 2.461533 0.445938
C -1.332283 2.759320 -0.422615
C -2.174708 1.506123 -0.258586
C -3.165231 1.376981 0.633289
C -3.818538 0.086697 0.814646
C -3.314481 -0.960190 0.011816
C -2.290987 -0.783731 -0.915375
O -1.742634 0.497202 -1.035741
O -4.750157 -0.047943 1.648763
H 0.791484 1.773324 -2.020389
H 2.891387 0.492219 -2.379478
H 1.589068 1.786251 2.249604
H -0.371737 2.639874 1.500885
H -1.892439 3.585660 0.034630
H 3.575091 0.614820 2.865093
H 4.940924 -0.445864 0.305761
H -3.740572 -1.955110 0.095315
O -1.771524 -1.620801 -1.665054
H -3.467877 2.229253 1.232651
CU 0.714809 -1.388695 0.205062
O -1.162697 -3.891666 -0.102994
O -0.323419 -0.740399 1.723793
H 0.092894 -0.994186 2.557647
H -1.193703 -1.166474 1.725417
H -0.394033 -3.571158 0.385009
H -1.391023 -3.145279 -0.689519
O 1.574038 -2.289919 -1.294855
H 1.378629 -3.235934 -1.262059
H 2.537727 -2.223313 -1.280930
O -0.984939 3.039191 -1.750412
H -1.790475 3.269306 -2.229769

ENERGIES
SCF ENERGY: -1379.01513299
SUM OF ELECTRONIC AND ZERO-POINT ENERGIES: -1378.727905
SUM OF ELECTRONIC AND THERMAL ENERGIES: -1378.701071
SUM OF ELECTRONIC AND THERMAL ENTHALPIES: -1378.700127
SUM OF ELECTRONIC AND THERMAL FREE ENERGIES: -1378.784565

==> AOX-II/HIS-/CU2+/MONO/UNIDENTATE/C4P/RAF/C2 <==
39
DATASET
C 0.472813 0.851496 -0.834766
C 1.107799 -0.048354 -1.699506
C 2.473533 0.021267 -1.900778
C 3.228302 0.985004 -1.245773
C 2.605083 1.917325 -0.414272
C 1.240688 1.844343 -0.211326
O 3.419631 2.841390 0.163673
O 4.581675 0.985235 -1.405663
C -0.940967 0.787762 -0.523422
C -1.790395 -0.194395 -0.877922
C -3.183603 -0.205570 -0.531619
C -4.014124 -1.233189 -0.868841
C -5.413072 -1.216050 -0.582486
C -5.850306 -0.115017 0.326984
C -4.877574 0.916049 0.665448
O -3.603002 0.900402 0.129071
O -6.235682 -2.009717 -1.039938
H 0.535512 -0.815476 -2.211337
H 2.984016 -0.679503 -2.554925
H 0.766038 2.562292 0.454047
H -1.323804 1.613001 0.074914
H -1.458824 -1.055711 -1.452019
H 2.910269 3.439372 0.725822
H 4.968299 1.740308 -0.936685
H -6.916156 0.095089 0.385167
O -5.149162 -0.073290 1.605258
H -3.603367 -2.056305 -1.442616
CU 3.228813 -1.606758 0.594962
O 3.595761 0.589837 2.617926
O 1.400577 -1.708812 1.245465
H 0.810866 -1.964830 0.522625
H 1.123360 -0.812574 1.484758
H 3.961899 0.534205 1.725531
H 3.185915 -0.274532 2.747123
O 5.052960 -1.465380 -0.045566
H 5.664649 -1.375853 0.696350
H 5.108623 -0.620432 -0.533453
O -5.144935 2.206048 0.971129
H -6.044521 2.277199 1.319641

ENERGIES
SCF ENERGY: -1379.00714498
SUM OF ELECTRONIC AND ZERO-POINT ENERGIES: -1378.719849
SUM OF ELECTRONIC AND THERMAL ENERGIES: -1378.693148
SUM OF ELECTRONIC AND THERMAL ENTHALPIES: -1378.692204
SUM OF ELECTRONIC AND THERMAL FREE ENERGIES: -1378.777403

==> AOX-II/HIS-/CU2+/MONO/UNIDENTATE/C4P/RAF/C2B <==
39
DATASET
C -0.111094 -1.163697 -0.617121
C -0.828421 -0.414857 -1.517771
C -2.202453 -0.610617 -1.699297
C -2.955470 -1.537559 -0.988715
C -2.298210 -2.322794 -0.060671
C -0.813344 -2.331162 0.001147
O -2.965497 -3.127276 0.721108
O -4.295995 -1.564445 -1.165046
C 1.270946 -0.977727 -0.295964
C 2.040379 0.010098 -0.801602
C 3.420768 0.221462 -0.467108
C 4.158218 1.214449 -1.011054
C 5.555424 1.390693 -0.653255
C 6.050517 0.466958 0.285394
C 5.274867 -0.553717 0.842895
O 3.939373 -0.648739 0.432005
O 6.237370 2.317947 -1.170565
H -0.352578 0.380533 -2.080361
H -2.730623 0.011857 -2.417780
H -0.582959 -3.136677 -0.738021
H 1.708778 -1.671615 0.416208
H 1.637766 0.728537 -1.511430
H -2.360624 -3.518768 1.381553
H -4.711157 -2.217625 -0.584629
H 7.084283 0.530219 0.609750
O 5.608633 -1.405490 1.667208
H 3.697563 1.886419 -1.727433
CU -2.947965 1.411560 0.295415
O -3.465232 3.943449 1.845892
O -1.650606 0.833961 1.621795
H -1.775524 -0.098920 1.839532
H -1.762693 1.303535 2.459109
H -3.845869 3.738982 0.984086
H -2.667829 3.402016 1.873451
O -4.238835 2.008904 -1.021826
H -5.014412 2.388352 -0.587941
H -4.571727 1.250360 -1.520711
O -0.408608 -2.717323 1.283344
H 0.280982 -3.389520 1.224981

ENERGIES
SCF ENERGY: -1378.99130828
SUM OF ELECTRONIC AND ZERO-POINT ENERGIES: -1378.703406
SUM OF ELECTRONIC AND THERMAL ENERGIES: -1378.676479
SUM OF ELECTRONIC AND THERMAL ENTHALPIES: -1378.675535
SUM OF ELECTRONIC AND THERMAL FREE ENERGIES: -1378.761964

==> AOX-II/HIS-/CU2+/MONO/UNIDENTATE/C4P/RAF/C2P <==
39
DATASET
C -0.110757 2.204062 -0.481834
C 0.869557 2.442947 -1.480997
C 2.148225 2.003556 -1.308279
C 2.502911 1.314651 -0.137685
C 1.568793 1.185128 0.927628
C 0.284084 1.601799 0.746402
O 2.038062 0.632738 2.068832
O 3.713453 0.804687 -0.068922
C -1.475918 3.083190 -0.459211
C -1.561524 1.804022 -1.109202
C -2.169661 0.550695 -0.650573
C -2.314360 -0.493563 -1.483247
C -2.853900 -1.755242 -0.996597
C -3.137448 -1.792519 0.386857
C -3.025121 -0.675759 1.221521
O -2.520185 0.503182 0.649016
O -3.012233 -2.728009 -1.777287
H 0.569371 2.931957 -2.403388
H 2.904760 2.127714 -2.075368
H -0.432131 1.477314 1.553618
H -1.486328 1.847107 -2.191001
H 1.333104 0.562762 2.727275
H 3.818345 0.120755 0.647383
H -3.544197 -2.697054 0.828125
O -3.287196 -0.577193 2.418347
H -2.017212 -0.401547 -2.522170
CU 1.207168 -1.670052 -0.306156
O 3.939345 -1.445774 1.395703
O -0.035838 -1.927188 1.151675
H -0.956640 -2.026158 0.843495
H -0.049967 -1.174070 1.758227
H 3.496273 -2.001601 0.738863
H 3.263485 -1.303002 2.071496
O 2.554339 -1.614434 -1.698664
H 2.988453 -2.474972 -1.769078
H 3.256489 -0.994570 -1.450541
H -1.363005 3.950391 -1.106259
O -2.059871 3.289721 0.747233
H -1.776975 4.146851 1.091672

ENERGIES
SCF ENERGY: -1379.01060162
SUM OF ELECTRONIC AND ZERO-POINT ENERGIES: -1378.721881
SUM OF ELECTRONIC AND THERMAL ENERGIES: -1378.696204
SUM OF ELECTRONIC AND THERMAL ENTHALPIES: -1378.695260
SUM OF ELECTRONIC AND THERMAL FREE ENERGIES: -1378.778187

==> AOX-II/HIS-/CU2+/MONO/UNIDENTATE/C4P/RAF/C3 <==
39
DATASET
C 0.394572 0.750098 -0.690522
C 1.058402 -0.218640 -1.455179
C 2.410913 -0.099903 -1.714219
C 3.130657 0.982596 -1.220686
C 2.481802 1.957253 -0.455156
C 1.130961 1.838615 -0.198744
O 3.260365 2.981147 -0.009463
O 4.457928 1.069117 -1.479446
C -1.018200 0.689657 -0.385744
C -1.868119 -0.302392 -0.720789
C -3.263910 -0.289791 -0.417401
C -4.111605 -1.306873 -0.722053
C -5.499792 -1.212185 -0.413587
C -5.981745 0.190453 -0.085109
C -4.969473 0.983803 0.679749
O -3.679137 0.833652 0.260393
O -6.321166 -2.120485 -0.446309
H 0.516108 -1.067523 -1.858729
H 2.932147 -0.836257 -2.318809
H 0.636637 2.605715 0.393511
H -1.411356 1.541523 0.166446
H -1.532461 -1.183785 -1.260686
H 2.728749 3.624910 0.476344
H 4.804680 1.901342 -1.124309
H -6.016927 0.695498 -1.073174
O -5.207299 1.768387 1.547964
H -3.712197 -2.210687 -1.167086
CU 3.456302 -1.494584 0.714557

O 5.323721 0.711364 1.550309
O 2.139643 -0.816377 1.971901
H 1.328868 -0.593395 1.491940
H 2.457500 0.025636 2.326008
H 5.162855 0.372190 0.659484
H 4.819148 0.112527 2.113951
O 4.862455 -2.247717 -0.393426
H 5.698630 -1.806580 -0.192082
H 4.695069 -2.059651 -1.326402
O -7.222858 0.209757 0.544672
H -7.616212 -0.665430 0.395333

ENERGIES
SCF ENERGY: -1379.04617763
SUM OF ELECTRONIC AND ZERO-POINT ENERGIES: -1378.756157
SUM OF ELECTRONIC AND THERMAL ENERGIES: -1378.729985
SUM OF ELECTRONIC AND THERMAL ENTHALPIES: -1378.729041
SUM OF ELECTRONIC AND THERMAL FREE ENERGIES: -1378.812929

==> AOX-II/HIS-/CU2+/MONO/UNIDENTATE/C4P/RAF/C3B <==
39
DATASET
C -0.034348 1.181869 -0.337890
C 0.584728 0.688811 -1.555087
C 1.909210 0.817443 -1.846536
C 2.738066 1.457793 -0.922958
C 2.148696 2.234952 0.247008
C 0.715298 1.909269 0.511969
O 2.981967 1.959750 1.336619
O 4.007041 1.493571 -1.104454
C -1.430527 0.909491 -0.035374
C -2.273909 0.187718 -0.785831
C -3.651748 -0.072261 -0.433882
C -4.481749 -0.817846 -1.191405
C -5.854082 -1.056549 -0.781222
C -6.230950 -0.460230 0.437692
C -5.362747 0.312770 1.209103
O -4.056771 0.486873 0.732083
O -6.625225 -1.757422 -1.495184
H -0.037606 0.163574 -2.274296
H 2.345863 0.396021 -2.744601
H 0.304022 2.338979 1.422317
H -1.787511 1.337739 0.898526
H -1.964355 -0.262876 -1.725544
H 2.719815 2.523649 2.081696
H 4.480564 1.892014 -0.346236
H -7.240682 -0.592113 0.813391
O -5.583776 0.881822 2.281104
H -4.115782 -1.247131 -2.118031
CU 3.013749 -1.412448 0.181480
O 3.741594 -3.977532 1.571416
O 2.432790 -0.748021 1.896570
H 2.664586 0.200711 1.921770
H 2.942596 -1.165849 2.602797
H 3.953621 -3.728722 0.663899
H 2.999377 -3.403781 1.795368
O 3.597946 -2.058852 -1.547107
H 4.285844 -2.731649 -1.457293
H 4.000088 -1.342501 -2.056438
O 2.169283 3.589141 -0.111249
H 3.076398 3.868897 -0.312140

ENERGIES
SCF ENERGY: -1378.98331500
SUM OF ELECTRONIC AND ZERO-POINT ENERGIES: -1378.694039
SUM OF ELECTRONIC AND THERMAL ENERGIES: -1378.667772
SUM OF ELECTRONIC AND THERMAL ENTHALPIES: -1378.666828
SUM OF ELECTRONIC AND THERMAL FREE ENERGIES: -1378.751741

==> AOX-II/HIS-/CU2+/MONO/UNIDENTATE/C4P/RAF/C4 <==
39
DATASET
C -0.337167 -1.208032 -0.180260
C -0.930264 -0.989387 -1.429544
C -2.264325 -1.292221 -1.636650
C -3.031804 -1.816436 -0.606687
C -2.456576 -2.046181 0.643199
C -1.122715 -1.744723 0.848388
O -3.281176 -2.557436 1.599992
O -4.357183 -2.064513 -0.824219
C 1.052776 -0.889942 0.113006
C 1.937510 -0.341174 -0.733540
C 3.310098 -0.050897 -0.408096
C 4.198730 0.477340 -1.258427
C 5.594010 0.636721 -0.852161
C 5.927095 0.370868 0.554642
C 4.830257 -0.037119 1.468347
O 3.647612 -0.370570 0.902894
O 6.251794 -0.567018 -0.473510
H -0.350723 -0.576786 -2.249084
H -2.738154 -1.118973 -2.598553
H -0.687309 -1.925798 1.829071
H 1.378370 -1.129677 1.123695
H 1.662947 -0.085681 -1.754495
H -2.794750 -2.708055 2.420913
H -4.738752 -2.491556 -0.042808
H 6.770740 0.860932 1.034450
O 4.934196 -0.084603 2.665120
H 3.896483 0.746307 -2.263429
CU -3.444127 1.229797 -0.115236
O -4.966404 3.471253 1.211640
O -1.756477 1.843388 0.605004
H -1.163533 1.091715 0.751565
H -1.876742 2.250585 1.472772
H -5.118090 3.087161 0.339416
H -4.116963 3.098974 1.478617
O -5.113305 0.647397 -0.908111
H -5.864496 0.902174 -0.357644
H -5.094462 -0.330036 -0.905693
O 6.300524 1.510081 -1.628445
H 7.192705 1.627864 -1.273185

ENERGIES
SCF ENERGY: -1379.00408210
SUM OF ELECTRONIC AND ZERO-POINT ENERGIES: -1378.717696
SUM OF ELECTRONIC AND THERMAL ENERGIES: -1378.692194
SUM OF ELECTRONIC AND THERMAL ENTHALPIES: -1378.691250
SUM OF ELECTRONIC AND THERMAL FREE ENERGIES: -1378.775253

==> AOX-II/HIS-/CU2+/MONO/UNIDENTATE/C4P/RAF/C4B <==
39
DATASET
C 0.081854 1.241125 -0.198088
C 0.761699 0.594372 -1.299615
C 2.057507 0.815548 -1.532282
C 2.879874 1.778417 -0.734976
C 2.104496 2.397641 0.411060
C 0.785165 2.126487 0.635851
O 2.839893 3.199022 1.134881
O 3.984154 1.066327 -0.253227
C -1.288925 1.007742 0.083757
C -2.086145 0.169298 -0.630333
C -3.470491 -0.052184 -0.355178
C -4.225648 -0.887785 -1.105874
C -5.638129 -1.085628 -0.818233
C -6.121562 -0.349791 0.276915
C -5.326229 0.508954 1.048381
O -3.978072 0.633906 0.694483
O -6.331302 -1.864177 -1.525573

H	0.213765	-0.084687	-1.941835
H	2.582588	0.344308	-2.359799
H	0.278244	2.604267	1.469749
H	-1.711905	1.546354	0.927216
H	-1.704041	-0.392046	-1.478348
H	2.338061	3.588205	1.870701
H	4.541936	1.661205	0.270306
H	-7.164935	-0.434328	0.563206
O	-5.659402	1.185196	2.017889
H	-3.767453	-1.413344	-1.936921
CU	2.440505	-1.611554	0.175909
O	4.414516	-3.655862	1.672916
O	1.646664	-1.053582	1.855401
H	1.787983	-0.106868	1.993710
H	2.085109	-1.488918	2.598462
H	4.099702	-4.136526	0.899813
H	3.808786	-2.906566	1.740034
O	3.255885	-2.317001	-1.436330
H	3.862904	-3.033580	-1.208583
H	3.806207	-1.637404	-1.848130
O	3.257697	2.869104	-1.534531
H	3.647358	2.536380	-2.356525

ENERGIES
SCF ENERGY: -1378.99612309
SUM OF ELECTRONIC AND ZERO-POINT ENERGIES: -1378.708001
SUM OF ELECTRONIC AND THERMAL ENERGIES: -1378.680894
SUM OF ELECTRONIC AND THERMAL ENTHALPIES: -1378.679950
SUM OF ELECTRONIC AND THERMAL FREE ENERGIES: -1378.767348

==> AOX-II/HIS-/CU2+/MONO/UNIDENTATE/C4P/RAF/C5 <==
39
DATASET

C	0.152304	1.346149	-0.048948
C	0.647449	1.380807	-1.369290
C	1.974209	1.644230	-1.604140
C	2.839307	1.874855	-0.535049
C	2.364993	1.849738	0.788656
C	1.036185	1.597165	1.022514
O	3.297631	2.065839	1.749687
O	4.139991	2.083482	-0.795213
C	-1.198465	1.051894	0.256540
C	-2.197680	0.745858	-0.641076
C	-3.487966	0.462744	-0.221797
C	-4.580027	0.132669	-1.162581
C	-5.502869	-0.959578	-0.607324
C	-5.711454	-0.935277	0.769968
C	-4.967831	-0.126735	1.625474
O	-3.801764	0.543325	1.047485
O	-6.025050	-1.710700	-1.445855
H	-0.014379	1.195284	-2.207914
H	2.381561	1.667194	-2.609658
H	0.668286	1.566397	2.045458
H	-1.458514	1.061977	1.314247
H	-2.015687	0.706085	-1.708363
H	2.900749	2.017699	2.629456
H	4.629827	2.230242	0.030118
H	-6.464680	-1.568975	1.224504
O	-5.069857	0.088529	2.813300
H	-5.218998	1.040286	-1.168620
CU	3.417620	-1.121820	0.060668
O	4.548711	-3.906294	0.247507
O	2.298467	-1.530565	1.576911
H	2.350450	-0.823124	2.233813
H	2.621123	-2.324497	2.023971
H	4.993409	-3.123355	-0.099274
H	3.619666	-3.646395	0.270286
O	4.529366	-0.722129	-1.469231
H	5.396823	-1.139863	-1.388961
H	4.695071	0.236444	-1.452786
O	-4.128710	0.169788	-2.446773
H	-4.714905	-0.883299	-2.755845

ENERGIES
SCF ENERGY: -1379.02348449
SUM OF ELECTRONIC AND ZERO-POINT ENERGIES: -1378.736314
SUM OF ELECTRONIC AND THERMAL ENERGIES: -1378.709545
SUM OF ELECTRONIC AND THERMAL ENTHALPIES: -1378.708601
SUM OF ELECTRONIC AND THERMAL FREE ENERGIES: -1378.794193

==> AOX-II/HIS-/CU2+/MONO/UNIDENTATE/C4P/RAF/C5B <==
39
DATASET

C	-0.816262	0.891128	-0.300630
C	-0.006809	-0.092153	-0.770763
C	1.376453	0.199527	-1.168203
C	1.940491	1.472615	-0.679989
C	1.071100	2.485571	-0.243674
C	-0.247833	2.179736	-0.043499
O	1.658986	3.669106	0.020504
O	3.219219	1.633244	-0.760564
C	-2.235774	0.704668	-0.010098
C	-2.999842	-0.257763	-0.539950
C	-4.400455	-0.453294	-0.241399
C	-5.153795	-1.412298	-0.817068
C	-6.557805	-1.561168	-0.479230
C	-7.049418	-0.644779	0.470752
C	-6.258370	0.342397	1.058508
O	-4.913419	0.407700	0.670947
O	-7.258057	-2.461801	-1.022006
H	-0.355190	-1.102504	-0.960667
H	-0.895980	2.962518	0.347084
H	-2.677241	1.428394	0.671722
H	-2.590617	-0.961075	-1.262529
H	1.040694	4.287567	0.434361
H	3.482552	2.542684	-0.520821
H	-8.090019	-0.690492	0.775927
O	-6.579529	1.190727	1.895021
H	-4.701794	-2.080086	-1.542855
CU	4.702099	-0.617196	0.382334
O	6.362264	-2.949626	1.344672
O	3.586344	-0.611299	1.974488
H	2.730743	-0.219616	1.751311
H	3.953697	-0.051409	2.670527
H	6.638752	-2.236042	0.757397
H	5.464574	-2.699212	1.594900
O	5.612604	-0.631853	-1.330972
H	6.453898	-1.105316	-1.307028
H	5.826781	0.269940	-1.603594
H	1.202133	0.516826	-2.239043
O	2.204740	-0.915149	-1.105083
H	3.040848	-0.736994	-1.558983

ENERGIES
SCF ENERGY: -1378.97838529
SUM OF ELECTRONIC AND ZERO-POINT ENERGIES: -1378.692248
SUM OF ELECTRONIC AND THERMAL ENERGIES: -1378.664754
SUM OF ELECTRONIC AND THERMAL ENTHALPIES: -1378.663810
SUM OF ELECTRONIC AND THERMAL FREE ENERGIES: -1378.751781

==> AOX-II/HIS-/CU2+/MONO/UNIDENTATE/C4P/RAF/C6 <==
39
DATASET

C	-0.067717	-0.972134	0.375689
C	-0.561815	-1.507344	-0.832093
C	-1.843203	-1.996735	-0.898421
C	-2.661064	-1.965441	0.230937
C	-2.190453	-1.428261	1.442127
C	-0.908004	-0.944835	1.509523

O -3.081191 -1.416565 2.465194
O -3.916255 -2.430336 0.126644
C 1.239157 -0.442339 0.501916
C 2.202328 -0.384546 -0.480880
C 3.446708 0.177741 -0.243067
C 4.494076 0.267303 -1.283228
C 5.895311 0.026783 -0.708176
C 6.111444 0.503365 0.583187
C 5.077431 0.960641 1.395305
O 3.712824 0.723140 0.917539
O 6.715715 -0.502012 -1.474306
H 0.064935 -1.532511 -1.716727
H -2.249329 -2.409200 -1.816316
H -0.543221 -0.524125 2.443456
H 1.497773 -0.043688 1.482303
H 2.029600 -0.774959 -1.476586
H -2.675630 -1.060014 3.266530
H -4.372324 -2.363451 0.980791
H 7.110158 0.517916 1.004994
O 5.087430 1.487199 2.485923
H 4.505006 1.340782 -1.566020
CU -3.660010 0.899307 -0.337626
O -5.430597 3.207665 -1.133098
O -2.536198 2.001096 0.780289
H -2.451336 1.607343 1.659584
H -2.935051 2.870021 0.921967
H -5.044233 2.542377 -1.715663
H -4.998800 3.041762 -0.286055
O -4.771242 -0.186811 -1.489114
H -5.681436 0.136072 -1.473264
H -4.796304 -1.079375 -1.102735
O 4.239791 -0.538666 -2.392381
H 5.113076 -0.871738 -2.665509

ENERGIES
SCF ENERGY: -1379.02354384

==> AOX-II/HIS-/CU2+/MONO/UNIDENTATE/C4P/RAF/C6B <==
39
DATASET
C 0.082625 0.813689 -0.640874
C 0.722037 -0.292571 -1.424084
C 2.197015 -0.206748 -1.570666
C 2.886661 0.902137 -1.254695
C 2.190219 2.001610 -0.649643
C 0.830140 1.923826 -0.336522
O 2.922817 3.050794 -0.398344
O 4.225494 0.973600 -1.437530
C -1.296609 0.787495 -0.271305
C -2.205278 -0.118834 -0.696311
C -3.592761 -0.109246 -0.327060
C -4.470203 -1.025026 -0.792208
C -5.866945 -0.995404 -0.389805
C -6.206447 0.042633 0.496169
C -5.287278 0.985900 0.967695
O -3.962828 0.874275 0.526929
O -6.678012 -1.856418 -0.827926
H 2.699722 -1.056137 -2.025766
H 0.373802 2.771786 0.167180
H -1.621800 1.603133 0.370184
H -1.924618 -0.916858 -1.375942
H 2.422994 3.763570 0.034664
H 4.573303 1.823601 -1.127491
H -7.229189 0.138733 0.846461
O -5.485302 1.925800 1.737041
H -4.122155 -1.794225 -1.473459
CU 3.229600 -1.470051 0.839710
O 3.527393 1.231308 2.048354
O 1.448204 -1.629743 1.585933
H 0.880005 -1.683912 0.787493
H 1.200146 -0.807519 2.028851
H 4.142071 0.857297 1.404370
H 3.179662 0.454405 2.502732
O 4.982116 -1.348790 0.022435
H 5.660764 -1.221573 0.697618
H 4.981358 -0.529244 -0.506743
H 0.322965 -0.150476 -2.451847
O 0.309241 -1.550201 -0.926273
H 0.590540 -2.242888 -1.538980

ENERGIES
SCF ENERGY: -1379.00617109
SUM OF ELECTRONIC AND ZERO-POINT ENERGIES: -1378.717394
SUM OF ELECTRONIC AND THERMAL ENERGIES: -1378.691463
SUM OF ELECTRONIC AND THERMAL ENTHALPIES: -1378.690519
SUM OF ELECTRONIC AND THERMAL FREE ENERGIES: -1378.773725

==> AOX-II/HIS2- <==
26
DATASET
C 1.743627 0.417083 -0.000013
C 2.513036 1.588232 -0.000020
C 3.895137 1.547388 -0.000008
C 4.620540 0.330650 0.000009
C 3.809187 -0.855054 0.000011
C 2.438729 -0.811865 0.000002
O 4.506732 -2.041373 0.000022
O 5.902728 0.264427 0.000022
C 0.306944 0.511961 -0.000018
C -0.583094 -0.504766 0.000026
C -2.011478 -0.344624 0.000016
C -2.893056 -1.375594 0.000088
C -4.317423 -1.140692 0.000076
C -4.715204 0.214592 -0.000011
C -3.805834 1.267568 -0.000100
O -2.443762 0.944954 -0.000071
O -5.139753 -2.105232 0.000148
H 2.001383 2.548994 -0.000033
H 4.471980 2.469976 -0.000012
H 1.891981 -1.754241 0.000003
H -0.082815 1.530150 -0.000058
H -0.249692 -1.540597 0.000075
H 3.882395 -2.776798 0.000022
H -5.770959 0.467306 -0.000022
O -4.030168 2.484668 -0.000182
H -2.517145 -2.393609 0.000156

ENERGIES
SCF ENERGY: -876.313150255
SUM OF ELECTRONIC AND ZERO-POINT ENERGIES: -876.133055
SUM OF ELECTRONIC AND THERMAL ENERGIES: -876.118059
SUM OF ELECTRONIC AND THERMAL ENTHALPIES: -876.117115
SUM OF ELECTRONIC AND THERMAL FREE ENERGIES: -876.177005

==> AOX-II/HIS2-/AIP <==
26
DATASET
C 1.739945 0.402889 -0.000015
C 2.513253 1.603501 -0.000043
C 3.869471 1.574359 -0.000032
C 4.593420 0.328384 0.000005
C 3.779390 -0.889892 0.000031
C 2.415116 -0.839230 0.000022
O 4.489950 -2.030623 0.000061
O 5.836653 0.268550 0.000015
C 0.321027 0.525907 -0.000026
C -0.572381 -0.499970 0.000009
C -1.994196 -0.329934 -0.000004
C -2.863993 -1.369837 0.000043
C -4.297498 -1.145818 0.000043

C -4.692739 0.205619 -0.000009
C -3.787356 1.269220 -0.000112
O -2.423484 0.956737 -0.000069
O -5.103661 -2.118279 0.000125
H 1.982805 2.551739 -0.000072
H 4.459943 2.485464 -0.000052
H 1.858819 -1.773207 0.000041
H -0.063575 1.543801 -0.000064
H -0.242214 -1.535817 0.000051
H 3.898099 -2.795634 0.000071
H -5.748441 0.457724 0.000030
O -4.025557 2.478403 -0.000078
H -2.477403 -2.383558 0.000095

ENERGIES
SCF ENERGY: -876.151457045
SUM OF ELECTRONIC AND ZERO-POINT ENERGIES: -875.970463
SUM OF ELECTRONIC AND THERMAL ENERGIES: -875.955598
SUM OF ELECTRONIC AND THERMAL ENTHALPIES: -875.954654
SUM OF ELECTRONIC AND THERMAL FREE ENERGIES: -876.014743

==> AOX-II/HIS2-/CU2+/BIS <==
53
DATASET
C 5.167317 -0.502295 -0.313597
C 4.792939 -1.849807 -0.412640
C 3.475498 -2.220509 -0.609338
C 2.460681 -1.257806 -0.710800
C 2.855453 0.088285 -0.630580
C 4.161082 0.468730 -0.442442
O 1.811520 0.990947 -0.781608
O 1.187494 -1.568145 -0.863476
C 6.529774 -0.060385 -0.087062
C 7.601189 -0.834008 0.163396
C 8.935346 -0.333078 0.383409
C 9.995481 -1.116734 0.686236
C 11.307267 -0.546565 0.907215
C 11.392164 0.855877 0.779614
C 10.295968 1.653920 0.461115
O 9.064783 1.014210 0.271437
O 12.294018 -1.284860 1.195912
H 5.547954 -2.627009 -0.331146
H 3.197511 -3.268887 -0.673759
H 4.408751 1.526528 -0.382358
H 6.674343 1.019083 -0.117720
H 7.513233 -1.916583 0.226638
H 1.963952 1.818555 -0.305316
H 12.344676 1.354981 0.927350
O 10.248565 2.880693 0.316486
H 9.855533 -2.189544 0.768799
CU 0.013756 -0.074478 -0.673470
C -5.126186 0.423092 -0.001116
C -4.715418 1.763931 0.011193
C -3.388620 2.114574 -0.145882
C -2.400030 1.136679 -0.325213
C -2.826751 -0.202726 -0.344957
C -4.142780 -0.563202 -0.181842
O -1.808001 -1.117469 -0.587108
O -1.122598 1.433129 -0.463196
C -6.510902 0.015388 0.147134
C -7.581764 0.828613 0.177260
C -8.947704 0.395705 0.332437
C -10.005511 1.238311 0.315234
C -11.357173 0.747018 0.473588
C -11.480571 -0.647810 0.643991
C -10.384447 -1.507770 0.663584
O -9.113730 -0.941462 0.499464
O -12.340257 1.544246 0.455607
H -5.449030 2.551683 0.157385
H -3.081954 3.156585 -0.122574
H -4.418239 -1.615520 -0.203216
H -6.674592 -1.058796 0.228625
H -7.469319 1.905731 0.072763
H -1.950245 -1.960728 -0.136506
H -12.462653 -1.092127 0.771776
O -10.369152 -2.734991 0.810457
H -9.833795 2.300949 0.178456

ENERGIES
SCF ENERGY: -1949.78238079
SUM OF ELECTRONIC AND ZERO-POINT ENERGIES: -1949.416905
SUM OF ELECTRONIC AND THERMAL ENERGIES: -1949.383992
SUM OF ELECTRONIC AND THERMAL ENTHALPIES: -1949.383047
SUM OF ELECTRONIC AND THERMAL FREE ENERGIES: -1949.487436

==> AOX-II/HIS2-/CU2+/BIS/AEA <==
53
DATASET
C 5.545596 -0.262404 -0.225798
C 5.070131 -1.578762 -0.157609
C 3.722934 -1.849937 -0.284999
C 2.760004 -0.840855 -0.487224
C 3.253105 0.486076 -0.560673
C 4.600118 0.755462 -0.432609
O 2.331177 1.481551 -0.773735
O 1.494938 -1.151623 -0.619755
C 6.943219 0.092835 -0.100804
C 7.969661 -0.747700 0.132324
C 9.350675 -0.355541 0.242021
C 10.359870 -1.221403 0.497342
C 11.728386 -0.766893 0.597085
C 11.926045 0.617685 0.409042
C 10.882660 1.501468 0.145543
O 9.588892 0.971155 0.069555
O 12.667710 -1.581255 0.839121
H 5.762000 -2.402339 -0.002697
H 3.361341 -2.873994 -0.230281
H 4.934632 1.790322 -0.498235
H 7.163993 1.154640 -0.209281
H 7.800249 -1.815362 0.256292
H 2.779487 2.336393 -0.779126
H 12.927019 1.033466 0.467962
O 10.933890 2.723468 -0.037257
H 10.133073 -2.274196 0.630268
CU 0.017109 -0.045590 -0.306032
C -5.535422 0.228631 0.100068
C -5.045566 1.538171 0.196469
C -3.689312 1.788011 0.167452
C -2.727843 0.764271 0.040356
C -3.234544 -0.556831 -0.049788
C -4.591776 -0.804323 -0.021441
O -2.316966 -1.572665 -0.166475
O -1.454863 1.063124 0.017359
C -6.944407 -0.102021 0.114729
C -7.970917 0.764725 0.214643
C -9.364464 0.404116 0.216181
C -10.372228 1.303904 0.307996
C -11.754998 0.883477 0.293418
C -11.968052 -0.507220 0.184788
C -10.925943 -1.426285 0.090484
O -9.617685 -0.926970 0.111943
O -12.691453 1.732823 0.370496
H -5.733600 2.373630 0.293163
H -3.317120 2.807158 0.240221
H -4.935086 -1.835812 -0.095723
H -7.174245 -1.164260 0.032090
H -7.790807 1.834590 0.297773
H -2.781274 -2.416468 -0.231503
H -12.980114 -0.899209 0.165340
O -10.989837 -2.656838 -0.014201

H -10.133717 2.359433 0.388278

ENERGIES
SCF ENERGY: -1949.94108881
SUM OF ELECTRONIC AND ZERO-POINT ENERGIES: -1949.576856
SUM OF ELECTRONIC AND THERMAL ENERGIES: -1949.543313
SUM OF ELECTRONIC AND THERMAL ENTHALPIES: -1949.542369
SUM OF ELECTRONIC AND THERMAL FREE ENERGIES: -1949.650313

==> AOX-II/HIS2-/CU2+/BIS/AIP <==
53
DATASET
C 5.495831 -0.200972 -0.079099
C 5.034987 -1.548263 -0.088318
C 3.704313 -1.828064 -0.125305
C 2.724710 -0.793417 -0.150304
C 3.200432 0.567913 -0.140146
C 4.548009 0.834293 -0.109696
O 2.254529 1.517845 -0.165483
O 1.483757 -1.083764 -0.183714
C 6.883841 0.145261 -0.040308
C 7.914409 -0.732451 0.018687
C 9.297385 -0.361614 0.055336
C 10.297906 -1.272747 0.122087
C 11.687680 -0.860678 0.153530
C 11.897334 0.531017 0.113901
C 10.856930 1.462611 0.043901
O 9.547328 0.970083 0.017456
O 12.612096 -1.718940 0.209857
H 5.748649 -2.364730 -0.067043
H 3.340861 -2.850745 -0.133518
H 4.881688 1.869476 -0.105488
H 7.109047 1.209745 -0.056789
H 7.739751 -1.804895 0.043967
H 2.653046 2.400448 -0.171239
H 12.908393 0.925114 0.132538
O 10.934752 2.690799 0.000173
H 10.049928 -2.328558 0.150252
CU 0.000058 0.007976 -0.080421
C -5.494807 0.199450 0.060503
C -5.037018 1.546990 0.112825
C -3.706790 1.830061 0.114288
C -2.724425 0.799196 0.056822
C -3.196919 -0.562553 0.002710
C -4.544147 -0.832529 0.011727
O -2.248911 -1.508731 -0.056621
O -1.484390 1.093857 0.054581
C -6.882584 -0.149422 0.053694
C -7.915725 0.727418 0.051169
C -9.298725 0.355445 0.041936
C -10.301218 1.266641 0.020738
C -11.691082 0.853728 0.010830
C -11.898736 -0.538718 0.025447
C -10.856205 -1.470402 0.049045
O -9.546656 -0.977051 0.054906
O -12.616966 1.711955 -0.008568
H -5.752785 2.360743 0.155067
H -3.345454 2.852675 0.155713
H -4.875522 -1.867720 -0.027647
H -7.105513 -1.214460 0.042537
H -7.743015 1.800481 0.050697
H -2.645473 -2.391486 -0.092991
H -12.909637 -0.933612 0.020880
O -10.932192 -2.699310 0.065973
H -10.054736 2.323118 0.010810

ENERGIES
SCF ENERGY: -1949.58266196
SUM OF ELECTRONIC AND ZERO-POINT ENERGIES: -1949.217243
SUM OF ELECTRONIC AND THERMAL ENERGIES: -1949.183920
SUM OF ELECTRONIC AND THERMAL ENTHALPIES: -1949.182976
SUM OF ELECTRONIC AND THERMAL FREE ENERGIES: -1949.289257

==> AOX-II/HIS2-/CU2+/BIS/HAT/C3B <==
52
DATASET
C 5.436230 -0.135289 0.010209
C 4.932124 -1.509801 -0.051472
C 3.621377 -1.800029 -0.041644
C 2.650868 -0.738418 0.029794
C 3.146112 0.704806 0.080532
C 4.562780 0.910322 0.078438
O 2.312683 1.604395 0.119511
O 1.437784 -0.963994 0.053951
C 6.851143 0.131091 0.000664
C 7.827373 -0.793324 -0.099977
C 9.233775 -0.486250 -0.103226
C 10.189453 -1.433986 -0.213884
C 11.598393 -1.079669 -0.206624
C 11.866874 0.296036 -0.083837
C 10.867575 1.265724 0.033514
O 9.537672 0.828365 0.016170
O 12.485350 -1.972867 -0.301705
H 5.647000 -2.323832 -0.103077
H 3.254614 -2.820061 -0.083797
H 4.922615 1.934102 0.123916
H 7.128483 1.179887 0.077989
H 7.606961 -1.853662 -0.187740
H 12.894374 0.645264 -0.067973
O 10.995504 2.484993 0.154597
H 9.897359 -2.474689 -0.306152
CU 0.034244 0.314927 0.096833
C -5.459790 0.398158 0.072238
C -5.055658 1.737125 -0.019999
C -3.716885 2.071034 -0.003577
C -2.696123 1.105539 0.103294
C -3.116240 -0.243875 0.204708
C -4.454925 -0.575241 0.187309
O -2.132725 -1.193474 0.320757
O -1.437744 1.472655 0.110876
C -6.841783 -0.031859 0.053780
C -7.934030 0.744079 -0.081533
C -9.287514 0.250388 -0.099345
C -10.377570 1.036580 -0.259728
C -11.709440 0.472411 -0.280635
C -11.782354 -0.927311 -0.116880
C -10.655712 -1.728028 0.052226
O -9.407276 -1.094170 0.053854
O -12.724660 1.211809 -0.440874
H -5.796808 2.527241 -0.105211
H -3.408158 3.110996 -0.075894
H -4.735499 -1.625015 0.265697
H -6.990148 -1.106445 0.157524
H -7.848603 1.822626 -0.195955
H -2.532962 -2.070299 0.383046
H -12.748496 -1.422045 -0.120961
O -10.593336 -2.953352 0.205916
H -10.246164 2.106900 -0.380056

ENERGIES
SCF ENERGY: -1949.15190920
SUM OF ELECTRONIC AND ZERO-POINT ENERGIES: -1948.798701
SUM OF ELECTRONIC AND THERMAL ENERGIES: -1948.765411
SUM OF ELECTRONIC AND THERMAL ENTHALPIES: -1948.764466
SUM OF ELECTRONIC AND THERMAL FREE ENERGIES: -1948.870594

==> AOX-II/HIS2-/CU2+/BIS/RAF/C1B <==
55
DATASET

C -5.628210 -2.064093 -0.414868
C -4.986294 -1.781603 -1.735873
C -3.703270 -1.446387 -1.863317
C -2.828222 -1.338725 -0.708674
C -3.381114 -1.691222 0.604924
C -4.662603 -2.055638 0.724775
O -2.493427 -1.625397 1.620997
O -1.653085 -0.979221 -0.865894
C -6.704696 -1.062981 -0.077418
C -7.007934 0.024671 -0.788163
C -8.045622 0.973801 -0.438068
C -8.336266 2.058378 -1.182914
C -9.389832 2.974954 -0.785329
C -10.061548 2.640967 0.406620
C -9.750464 1.513460 1.166523
O -8.715794 0.688896 0.704255
O -9.656831 3.991011 -1.486953
H -5.632927 -1.869878 -2.606328
H -3.248970 -1.245276 -2.828324
H -5.068481 -2.329048 1.696971
H -7.243273 -1.299251 0.839313
H -6.471989 0.267689 -1.703432
H -2.918161 -1.887281 2.449960
H -10.862577 3.276666 0.770379
O -10.268220 1.124741 2.217066
H -7.773845 2.247407 -2.091152
CU -0.182146 -0.693327 0.294540
C 5.286852 0.052637 0.400359
C 4.850911 0.153606 1.728217
C 3.515239 0.008654 2.044296
C 2.527313 -0.242440 1.071174
C 2.979623 -0.341822 -0.267805
C 4.315258 -0.200762 -0.581259
O 2.030527 -0.585706 -1.230088
O 1.272632 -0.373289 1.425335
C 6.667574 0.197488 -0.008306
C 7.723665 0.479209 0.778682
C 9.078407 0.615330 0.307954
C 10.129945 0.919829 1.104328
C 11.465279 1.046604 0.563778
C 11.584606 0.831774 -0.825866
C 10.497604 0.518287 -1.637703
O 9.242203 0.416736 -1.026034
O 12.444380 1.334334 1.313488
H 5.564811 0.346054 2.524635
H 3.183170 0.086391 3.076767
H 4.619492 -0.286496 -1.623947
H 6.848781 0.064518 -1.074718
H 7.603902 0.630048 1.849518
H 2.453041 -0.609542 -2.097882
H 12.556319 0.910465 -1.303189
O 10.477522 0.310284 -2.856506
H 9.963748 1.072753 2.165659
O -6.200703 -3.377634 -0.445091
H -6.941632 -3.370615 -1.066891

ENERGIES
SCF ENERGY: -2025.57805087
SUM OF ELECTRONIC AND ZERO-POINT ENERGIES: -2025.197809
SUM OF ELECTRONIC AND THERMAL ENERGIES: -2025.163591
SUM OF ELECTRONIC AND THERMAL ENTHALPIES: -2025.162647
SUM OF ELECTRONIC AND THERMAL FREE ENERGIES: -2025.270277

==> AOX-II/HIS2-/CU2+/BIS/RAF/C1P <==
55
DATASET
C 5.223679 -1.046120 -0.723196
C 4.997641 -1.373942 0.664842
C 3.828449 -1.071996 1.260178
C 2.757420 -0.419235 0.547888
C 2.974437 -0.111982 -0.859562
C 4.150135 -0.414909 -1.449662
O 1.932342 0.480077 -1.484586
O 1.692700 -0.149573 1.147743
C 6.398193 -1.282223 -1.355442
C 7.637428 -1.855614 -0.753742
C 8.478544 -0.741273 -0.189461
C 8.778207 -0.597899 1.107165
C 9.566946 0.541563 1.556100
C 9.981487 1.425130 0.540742
C 9.663851 1.248986 -0.805828
O 8.884331 0.129786 -1.139421
O 9.838093 0.691207 2.779317
H 5.781006 -1.865557 1.232375
H 3.640462 -1.307024 2.302816
H 4.306296 -0.173623 -2.498688
H 6.492636 -0.992467 -2.402064
H 7.399128 -2.531292 0.076854
H 2.153701 0.651067 -2.410124
H 10.576982 2.295842 0.796658
O 9.972048 1.950769 -1.771966
H 8.425845 -1.329104 1.827277
CU 0.106807 0.748731 0.635815
C -5.387354 0.511522 0.130895
C -5.011390 1.805889 -0.252316
C -3.689798 2.200831 -0.201935
C -2.656061 1.345293 0.229434
C -3.048902 0.040194 0.616602
C -4.369468 -0.353975 0.562634
O -2.058274 -0.808539 1.046056
O -1.417320 1.771070 0.259003
C -6.751271 0.027382 0.106977
C -7.852041 0.723263 -0.236690
C -9.190165 0.190748 -0.249000
C -10.285014 0.911396 -0.587776
C -11.603091 0.317307 -0.579606
C -11.658152 -1.040625 -0.199256
C -10.526802 -1.774808 0.147299
O -9.291982 -1.115725 0.110110
O -12.623313 0.996759 -0.897670
H -5.760090 2.513312 -0.598459
H -3.405325 3.205840 -0.504372
H -4.624389 -1.368336 0.868493
H -6.876623 -1.013537 0.404756
H -7.785451 1.767670 -0.534361
H -2.443548 -1.667971 1.258193
H -12.613454 -1.555173 -0.169354
O -10.450393 -2.959493 0.493441
H -10.167490 1.952640 -0.869584
O 8.307659 -2.552188 -1.788787
H 9.074342 -2.998103 -1.408687

ENERGIES
SCF ENERGY: -2025.59504227
SUM OF ELECTRONIC AND ZERO-POINT ENERGIES: -2025.212947
SUM OF ELECTRONIC AND THERMAL ENERGIES: -2025.178090
SUM OF ELECTRONIC AND THERMAL ENTHALPIES: -2025.177146
SUM OF ELECTRONIC AND THERMAL FREE ENERGIES: -2025.287236

==> AOX-II/HIS2-/CU2+/BIS/RAF/C2 <==
55
DATASET
C -5.018784 -0.267372 -0.489460
C -4.616691 -0.187284 -1.833780
C -3.334644 -0.521471 -2.202671
C -2.361530 -0.955195 -1.271981
C -2.780714 -1.033330 0.085662
C -4.063255 -0.699791 0.450751
O -1.850878 -1.457677 0.998924
O -1.166637 -1.263517 -1.677579

C -6.336045 0.059460 -0.030754
C -7.381969 0.491697 -0.779168
C -8.667851 0.791428 -0.246289
C -9.716263 1.214914 -1.023469
C -10.984265 1.560537 -0.487610
C -11.168484 1.219415 0.955533
C -10.010249 0.743646 1.697538
O -8.767145 0.653462 1.102418
O -11.897430 2.113241 -1.112581
H -5.319322 0.139920 -2.595040
H -3.025556 -0.459130 -3.242919
H -4.346538 -0.773826 1.499957
H -6.496125 -0.062300 1.040146
H -7.289893 0.633162 -1.852640
H -2.244233 -1.458800 1.880772
H -12.003148 1.682283 1.477760
O -10.955490 -0.189175 1.278867
H -9.537208 1.350895 -2.084436
CU 0.370043 -1.640514 -0.668149
C 5.703961 -0.412426 0.146067
C 5.502715 -1.706770 0.643967
C 4.241353 -2.266628 0.663078
C 3.098858 -1.586511 0.194398
C 3.313244 -0.274522 -0.297127
C 4.575422 0.280831 -0.321183
O 2.211285 0.413709 -0.743136
O 1.923623 -2.161013 0.242708
C 6.994827 0.239394 0.090063
C 8.175794 -0.260031 0.502976
C 9.433780 0.436850 0.426012
C 10.608073 -0.076765 0.862521
C 11.837536 0.677000 0.762260
C 11.721819 1.959105 0.183735
C 10.510907 2.478128 -0.266995
O 9.370682 1.677621 -0.124215
O 12.932184 0.192338 1.175688
H 6.342094 -2.286277 1.018798
H 4.093621 -3.273071 1.047463
H 4.693071 1.291591 -0.710856
H 6.986023 1.243460 -0.333674
H 8.244759 -1.255242 0.936998
H 2.483758 1.289440 -1.045104
H 12.602879 2.583315 0.072579
O 10.287977 3.575746 -0.791253
H 10.622509 -1.071231 1.296402
O -9.784871 0.897106 3.024451
H -10.629584 0.960139 3.491336

ENERGIES
SCF ENERGY: -2025.56810064
SUM OF ELECTRONIC AND ZERO-POINT ENERGIES: -2025.187051
SUM OF ELECTRONIC AND THERMAL ENERGIES: -2025.152457
SUM OF ELECTRONIC AND THERMAL ENTHALPIES: -2025.151513
SUM OF ELECTRONIC AND THERMAL FREE ENERGIES: -2025.260127

==> AOX-II/HIS2-/CU2+/BIS/RAF/C2B <==
55
DATASET
C 5.417599 0.299990 0.160185
C 4.908900 1.546114 0.410251
C 3.543614 1.762974 0.658739
C 2.558867 0.771339 0.716972
C 3.053266 -0.542965 0.534128
C 4.412807 -0.789850 0.001306
O 2.288519 -1.572449 0.744467
O 1.327791 1.032637 1.031182
C 6.795336 -0.012304 -0.092170
C 7.817280 0.861403 0.010912
C 9.196230 0.539482 -0.240989
C 10.194511 1.446173 -0.150340
C 11.567882 1.077322 -0.435592
C 11.767098 -0.270860 -0.791104
C 10.728606 -1.199595 -0.875267
O 9.433387 -0.748999 -0.591635
O 12.492560 1.935614 -0.362551
H 5.567422 2.408939 0.430789
H 3.212393 2.778788 0.860857
H 4.183363 -0.708156 -1.091413
H 7.017818 -1.039915 -0.368910
H 7.645614 1.894492 0.304372
H 2.826828 -2.386854 0.657312
H 12.767130 -0.627129 -1.017135
O 10.789885 -2.395104 -1.172983
H 9.960013 2.466764 0.133217
CU -0.173675 -0.046476 0.691446
C -5.691860 -0.229064 0.014082
C -5.249703 -1.545167 0.204073
C -3.904862 -1.825107 0.332695
C -2.910652 -0.826951 0.281211
C -3.368826 0.499982 0.085492
C -4.714665 0.777769 -0.040290
O -2.415726 1.487446 0.021625
O -1.648677 -1.154391 0.398230
C -7.084869 0.136315 -0.131874
C -8.142324 -0.697465 -0.105230
C -9.516420 -0.296254 -0.260681
C -10.558672 -1.159646 -0.232196
C -11.918721 -0.698548 -0.398380
C -12.072190 0.691818 -0.584041
C -10.994333 1.573639 -0.612664
O -9.712046 1.035604 -0.444614
O -12.886026 -1.515765 -0.374984
H -5.965697 -2.361195 0.253060
H -3.568647 -2.848557 0.480847
H -5.021635 1.812378 -0.191148
H -7.272939 1.199701 -0.279129
H -8.006261 -1.767287 0.039430
H -2.845228 2.341186 -0.115613
H -13.063470 1.113985 -0.716030
O -11.004643 2.799975 -0.770817
H -10.365864 -2.217120 -0.083759
O 4.810061 -2.095035 0.317453
H 5.161639 -2.531745 -0.467050

ENERGIES
SCF ENERGY: -2025.56564689
SUM OF ELECTRONIC AND ZERO-POINT ENERGIES: -2025.184507
SUM OF ELECTRONIC AND THERMAL ENERGIES: -2025.149868
SUM OF ELECTRONIC AND THERMAL ENTHALPIES: -2025.148924
SUM OF ELECTRONIC AND THERMAL FREE ENERGIES: -2025.258275

==> AOX-II/HIS2-/CU2+/BIS/RAF/C2P <==
55
DATASET
C -5.812634 1.532312 -0.409002
C -5.093635 2.627221 0.189509
C -3.753036 2.589137 0.323961
C -2.980117 1.459823 -0.113803
C -3.698421 0.355956 -0.727760
C -5.040871 0.397736 -0.864274
O -2.919700 -0.673241 -1.145812
O -1.733181 1.462146 0.035315
C -7.152907 1.843145 -1.151162
C -7.263902 1.298597 0.193086
C -7.739956 -0.054695 0.506049
C -8.058820 -0.452158 1.748209
C -8.538510 -1.803134 1.989144
C -8.621674 -2.628560 0.849848
C -8.283749 -2.196951 -0.430971
O -7.843041 -0.869320 -0.564875

O -8.849017 -2.175175 3.154692
H -5.656042 3.490582 0.534851
H -3.204496 3.410030 0.774574
H -5.554887 -0.435163 -1.337449
H -7.312881 1.170467 -1.987737
H -7.465165 2.020177 0.981668
H -3.465774 -1.368326 -1.537368
H -8.969302 -3.651769 0.951195
O -8.318991 -2.817830 -1.496144
H -7.962055 0.241477 2.576467
CU -0.386237 0.169070 -0.293819
C 5.082632 -0.386987 -0.077175
C 4.599306 -1.527295 -0.732646
C 3.242861 -1.726848 -0.892654
C 2.278287 -0.816175 -0.416489
C 2.778747 0.326736 0.255732
C 4.134536 0.527461 0.409253
O 1.856205 1.220312 0.743529
O 1.002164 -1.051762 -0.598728
C 6.487962 -0.102438 0.119804
C 7.532226 -0.869810 -0.247390
C 8.914910 -0.528664 -0.031229
C 9.953177 -1.320820 -0.388196
C 11.319920 -0.918794 -0.141246
C 11.483819 0.336825 0.481954
C 10.410422 1.146360 0.844164
O 9.121751 0.672365 0.569744
O 12.286907 -1.665898 -0.473422
H 5.291188 -2.265778 -1.128705
H 2.875286 -2.611117 -1.407784
H 4.473903 1.423543 0.928181
H 6.700260 0.841068 0.622351
H 7.380335 -1.825335 -0.745072
H 2.316850 1.959080 1.161215
H 12.481282 0.706923 0.697273
O 10.428873 2.256586 1.388731
H 9.752386 -2.273976 -0.866367
O -7.508976 3.140999 -1.458080
H -7.747384 3.623751 -0.656436

ENERGIES
SCF ENERGY: -2025.58641833
SUM OF ELECTRONIC AND ZERO-POINT ENERGIES: -2025.204852
SUM OF ELECTRONIC AND THERMAL ENERGIES: -2025.170274
SUM OF ELECTRONIC AND THERMAL ENTHALPIES: -2025.169330
SUM OF ELECTRONIC AND THERMAL FREE ENERGIES: -2025.277349

==> AOX-II/HIS2-/CU2+/BIS/RAF/C3 <==
55
DATASET
C -5.144294 0.381027 -0.098849
C -4.674992 1.707568 -0.121869
C -3.328553 1.974679 -0.082694
C -2.351014 0.952617 -0.013134
C -2.836705 -0.386756 0.004469
C -4.183713 -0.650032 -0.038505
O -1.897666 -1.382021 0.065094
O -1.093366 1.261225 0.024407
C -6.527950 0.031508 -0.127009
C -7.588840 0.884619 -0.146760
C -8.939948 0.465556 -0.150447
C -10.012069 1.316072 -0.173403
C -11.336014 0.822811 -0.112210
C -11.456209 -0.638004 0.295014
C -10.341982 -1.475246 -0.246598
O -9.107197 -0.904686 -0.180935
O -12.376194 1.457997 -0.290499
H -5.378906 2.533460 -0.170760
H -2.966889 2.999421 -0.099710
H -4.516707 -1.686931 -0.022884
H -6.738478 -1.037284 -0.122489
H -7.447745 1.962173 -0.143878
H -2.337879 -2.240956 0.094783
H -11.275902 -0.632382 1.389518
O -10.441050 -2.597718 -0.646879
H -9.830024 2.380996 -0.263018
CU 0.391500 0.153711 0.324157
C 5.933203 -0.236035 0.188276
C 5.420130 -1.537898 0.262278
C 4.064937 -1.754563 0.405784
C 3.129828 -0.702701 0.486376
C 3.660168 0.609430 0.411860
C 5.015287 0.823806 0.267216
O 2.768757 1.651207 0.499897
O 1.856957 -0.965116 0.640685
C 7.341858 0.064633 0.045129
C 8.349828 -0.824649 -0.042143
C 9.742917 -0.485680 -0.174052
C 10.735905 -1.402922 -0.251185
C 12.119001 -1.000535 -0.373696
C 12.347695 0.391182 -0.413747
C 11.320437 1.328301 -0.335195
O 10.011310 0.845864 -0.214327
O 13.042172 -1.865342 -0.436236
H 6.087834 -2.393449 0.206388
H 3.673951 -2.767683 0.462286
H 5.377886 1.850105 0.217262
H 7.588375 1.125647 0.009520
H 8.154666 -1.894584 -0.011267
H 3.246920 2.486434 0.421506
H 13.360739 0.770377 -0.504509
O 11.398913 2.562176 -0.357017
H 10.485824 -2.458223 -0.215428
O -12.700029 -1.185825 -0.013587
H -13.291484 -0.429564 -0.162129

ENERGIES
SCF ENERGY: -2025.61058116
SUM OF ELECTRONIC AND ZERO-POINT ENERGIES: -2025.229883
SUM OF ELECTRONIC AND THERMAL ENERGIES: -2025.194838
SUM OF ELECTRONIC AND THERMAL ENTHALPIES: -2025.193894
SUM OF ELECTRONIC AND THERMAL FREE ENERGIES: -2025.304178

==> AOX-II/HIS2-/CU2+/BIS/RAF/C3B <==
55
DATASET
C 5.380114 -0.132001 -0.166809
C 4.904063 -1.448813 -0.577289
C 3.612848 -1.718286 -0.846415
C 2.610101 -0.700114 -0.734159
C 3.027318 0.704826 -0.286527
C 4.490034 0.867639 -0.038746
O 2.610264 1.556086 -1.324531
O 1.423101 -0.934682 -0.980609
C 6.788189 0.110689 0.101705
C 7.771469 -0.799019 0.040662
C 9.160900 -0.519069 0.323738
C 10.128672 -1.458363 0.287913
C 11.507099 -1.122176 0.591131
C 11.742468 0.229756 0.909360
C 10.733304 1.192106 0.937989
O 9.431297 0.771887 0.637506
O 12.407027 -2.008964 0.562311
H 5.628790 -2.251954 -0.673229
H 3.285904 -2.705847 -1.153885
H 4.799892 1.867456 0.265251
H 7.032679 1.133926 0.380042
H 7.575742 -1.834354 -0.227168
H 3.031726 2.419553 -1.212976
H 12.748690 0.561193 1.145569

O 10.824446 2.395629 1.196280
H 9.868731 -2.480203 0.032313
CU -0.112450 0.181796 -0.845657
C -5.573060 0.302731 -0.141348
C -5.163803 1.639887 -0.230135
C -3.836269 1.959900 -0.430731
C -2.831755 0.980339 -0.557910
C -3.257331 -0.367983 -0.470689
C -4.583639 -0.686057 -0.265660
O -2.287277 -1.331665 -0.600373
O -1.583928 1.333436 -0.744747
C -6.945999 -0.105382 0.068546
C -8.022793 0.697943 0.162812
C -9.371362 0.241666 0.381336
C -10.443270 1.064826 0.457414
C -11.773240 0.542760 0.680303
C -11.864818 -0.859761 0.807457
C -10.756169 -1.699160 0.732114
O -9.508023 -1.103848 0.511472
O -12.770359 1.319944 0.753136
H -5.891942 2.441136 -0.136588
H -3.524758 2.999665 -0.494808
H -4.867226 -1.736122 -0.202745
H -7.100900 -1.180696 0.154951
H -7.925781 1.778076 0.074198
H -2.688074 -2.207087 -0.527636
H -12.830417 -1.325085 0.978895
O -10.710442 -2.930528 0.836167
H -10.298309 2.134783 0.349776
O 2.305060 0.923390 0.901052
H 2.688540 1.674714 1.374405

ENERGIES
SCF ENERGY: -2025.58563692
SUM OF ELECTRONIC AND ZERO-POINT ENERGIES: -2025.204719
SUM OF ELECTRONIC AND THERMAL ENERGIES: -2025.169379
SUM OF ELECTRONIC AND THERMAL ENTHALPIES: -2025.168435
SUM OF ELECTRONIC AND THERMAL FREE ENERGIES: -2025.279517

==> AOX-II/HIS2-/CU2+/BIS/RAF/C4 <==
55
DATASET
C 5.160217 -0.067847 0.279079
C 4.681412 -1.200153 0.997116
C 3.350414 -1.370376 1.215606
C 2.376971 -0.436751 0.744223
C 2.865918 0.703016 0.012131
C 4.199870 0.870201 -0.197807
O 1.921440 1.572916 -0.438634
O 1.142822 -0.636997 0.977517
C 6.509140 0.161475 0.025682
C 7.585494 -0.654489 0.384763
C 8.892485 -0.320756 0.127348
C 9.978619 -1.260679 0.396487
C 11.271206 -0.978798 -0.266851
C 11.380234 0.176199 -1.137886
C 10.408261 1.135695 -1.165701
O 9.190203 0.858306 -0.405603
O 11.107493 -0.759257 1.128597
H 5.384815 -1.932858 1.379977
H 2.975253 -2.228243 1.765594
H 4.546771 1.741549 -0.750481
H 6.747216 1.076715 -0.515493
H 7.424156 -1.601023 0.891269
H 2.347784 2.296816 -0.916714
H 12.299092 0.346588 -1.687314
O 10.340210 2.232522 -1.731053
H 9.693704 -2.282354 0.634727
CU -0.360524 0.438075 0.606335
C -5.851951 0.363354 -0.039117
C -5.494591 1.663355 0.342005
C -4.167225 2.021542 0.465599
C -3.110219 1.121381 0.223355
C -3.483802 -0.187992 -0.169393
C -4.810712 -0.543851 -0.292991
O -2.469121 -1.077574 -0.425256
O -1.866310 1.517106 0.339961
C -7.219766 -0.087544 -0.183642
C -8.337781 0.615942 0.079438
C -9.678724 0.118757 -0.092063
C -10.791229 0.830213 0.205932
C -12.110527 0.270417 0.014840
C -12.147348 -1.043812 -0.497973
C -10.998007 -1.766889 -0.806601
O -9.763347 -1.144989 -0.583901
O -13.147076 0.940227 0.298588
H -6.262964 2.405224 0.542264
H -3.896698 3.032476 0.761006
H -5.052850 -1.561357 -0.598628
H -7.332638 -1.110663 -0.541846
H -8.284246 1.636751 0.452235
H -2.847453 -1.932786 -0.665501
H -13.102417 -1.529774 -0.671133
O -10.905715 -2.912203 -1.263759
H -10.687140 1.837075 0.596879
O 12.130540 -2.039473 -0.496137
H 12.303530 -2.092652 -1.444593

ENERGIES
SCF ENERGY: -2025.54525070
SUM OF ELECTRONIC AND ZERO-POINT ENERGIES: -2025.165628
SUM OF ELECTRONIC AND THERMAL ENERGIES: -2025.130854
SUM OF ELECTRONIC AND THERMAL ENTHALPIES: -2025.129910
SUM OF ELECTRONIC AND THERMAL FREE ENERGIES: -2025.239043

==> AOX-II/HIS2-/CU2+/BIS/RAF/C4B <==
55
DATASET
C 5.336728 0.293453 0.561091
C 4.811630 -0.709022 1.457480
C 3.550084 -0.632398 1.895386
C 2.582491 0.436503 1.482396
C 3.255679 1.503535 0.640128
C 4.541097 1.373980 0.172989
O 2.478520 2.510241 0.343947
O 1.606093 -0.158826 0.750338
C 6.665021 0.225832 0.038290
C 7.540300 -0.773173 0.296071
C 8.869191 -0.842095 -0.239863
C 9.708784 -1.864720 0.034164
C 11.049569 -1.903745 -0.528658
C 11.375479 -0.814209 -1.355162
C 10.495336 0.239221 -1.628258
O 9.225798 0.188677 -1.041892
O 11.825883 -2.862003 -0.265910
H 5.444791 -1.533326 1.764725
H 3.127481 -1.387260 2.554795
H 4.942916 2.130994 -0.495717
H 6.967265 1.038892 -0.616498
H 7.272808 -1.607198 0.938974
H 2.911439 3.130483 -0.267607
H 12.356634 -0.762862 -1.816213
O 10.688813 1.230856 -2.329887
H 9.373813 -2.666778 0.683431
CU 0.037612 0.782321 0.323149
C -5.443226 0.326186 -0.217806
C -5.137235 1.677325 -0.429460
C -3.829893 2.117925 -0.393719
C -2.741009 1.256699 -0.146192
C -3.063321 -0.108187 0.060773

C -4.370365 -0.546727 0.023538
O -2.020242 -0.970498 0.297479
O -1.520043 1.729746 -0.121506
C -6.786574 -0.211030 -0.232852
C -7.934050 0.468002 -0.424873
C -9.245113 -0.128960 -0.427434
C -10.392748 0.564218 -0.616648
C -11.677172 -0.099804 -0.606976
C -11.642592 -1.494210 -0.390919
C -10.458022 -2.199384 -0.197082
O -9.261836 -1.471828 -0.221810
O -12.747725 0.552709 -0.785422
H -5.930173 2.394366 -0.624366
H -3.600388 3.168118 -0.557924
H -4.569737 -1.605239 0.189299
H -6.852852 -1.285995 -0.066152
H -7.931022 1.542951 -0.592869
H -2.367444 -1.859582 0.443427
H -12.568222 -2.060888 -0.371751
O -10.301236 -3.410501 -0.001579
H -10.343897 1.636062 -0.778733
O 2.109546 1.116925 2.638746
H 1.646881 0.464669 3.183414

ENERGIES
SCF ENERGY: -2025.56732869
SUM OF ELECTRONIC AND ZERO-POINT ENERGIES: -2025.186590
SUM OF ELECTRONIC AND THERMAL ENERGIES: -2025.151701
SUM OF ELECTRONIC AND THERMAL ENTHALPIES: -2025.150757
SUM OF ELECTRONIC AND THERMAL FREE ENERGIES: -2025.261576

==> AOX-II/HIS2-/CU2+/BIS/RAF/C5 <==
55
DATASET
C 5.250031 -0.016688 0.245984
C 4.742957 -1.335701 0.449674
C 3.406949 -1.570671 0.452278
C 2.449998 -0.525048 0.251093
C 2.966726 0.807654 0.043423
C 4.306036 1.035582 0.044069
O 2.036964 1.780349 -0.143178
O 1.212158 -0.795325 0.263339
C 6.601308 0.283777 0.240365
C 7.666100 -0.618588 0.420029
C 8.966104 -0.223545 0.424047
C 10.117167 -1.135089 0.631451
C 11.243709 -0.823968 -0.360118
C 11.409902 0.517474 -0.685122
C 10.503939 1.503486 -0.275042
O 9.278257 1.079550 0.308520
O 11.935544 -1.793074 -0.729027
H 5.431511 -2.159999 0.604311
H 3.009365 -2.569216 0.606010
H 4.674631 2.047826 -0.110574
H 6.863132 1.328735 0.080768
H 7.481557 -1.675566 0.572411
H 2.475034 2.631336 -0.275711
H 12.268599 0.844861 -1.260919
O 10.588538 2.715838 -0.383608
H 10.537050 -0.876714 1.623789
CU -0.303204 0.289640 -0.024888
C -5.832861 0.329418 -0.185011
C -5.411020 1.652994 -0.002438
C -4.069492 1.975935 -0.036725
C -3.061266 1.014797 -0.251014
C -3.500097 -0.318853 -0.442474
C -4.841304 -0.639374 -0.409098
O -2.533388 -1.268128 -0.667258
O -1.802390 1.376972 -0.286650
C -7.219096 -0.085913 -0.155388
C -8.291970 0.685647 0.104868
C -9.654172 0.217491 0.115490
C -10.719986 1.000310 0.405442
C -12.063414 0.465301 0.403211
C -12.174613 -0.903656 0.079008
C -11.072885 -1.701541 -0.218774
O -9.810268 -1.097361 -0.188915
O -13.055888 1.201633 0.679612
H -6.140433 2.440501 0.167228
H -3.748393 3.004921 0.106352
H -5.134435 -1.677498 -0.562772
H -7.388794 -1.141789 -0.365298
H -8.181116 1.743777 0.332749
H -2.949993 -2.135219 -0.748318
H -13.151807 -1.375682 0.055371
O -11.045286 -2.902139 -0.513829
H -10.559392 2.046307 0.645263
O 9.757718 -2.486019 0.584642
H 10.498217 -2.921883 0.126560

ENERGIES
SCF ENERGY: -2025.60130573
SUM OF ELECTRONIC AND ZERO-POINT ENERGIES: -2025.220116
SUM OF ELECTRONIC AND THERMAL ENERGIES: -2025.185380
SUM OF ELECTRONIC AND THERMAL ENTHALPIES: -2025.184436
SUM OF ELECTRONIC AND THERMAL FREE ENERGIES: -2025.293503

==> AOX-II/HIS2-/CU2+/BIS/RAF/C5B <==
55
DATASET
C -5.453891 0.159585 0.109302
C -4.949059 1.408386 0.075926
C -3.510991 1.650089 0.341639
C -2.591130 0.475746 0.165744
C -3.199639 -0.834903 0.296102
C -4.544901 -0.960630 0.245024
O -2.334141 -1.871156 0.353384
O -1.385531 0.668713 -0.013960
C -6.876805 -0.146373 -0.015472
C -7.871374 0.700357 0.280158
C -9.277824 0.402088 0.134087
C -10.260284 1.260618 0.478185
C -11.656297 0.902453 0.312972
C -11.891945 -0.379836 -0.221078
C -10.867235 -1.256264 -0.576425
O -9.549750 -0.823153 -0.379419
O -12.571933 1.711042 0.637832
H -5.574379 2.287204 -0.057451
H -4.969662 -1.962681 0.273641
H -7.118089 -1.151495 -0.357126
H -7.658363 1.691815 0.675394
H -2.817519 -2.708820 0.330696
H -12.910720 -0.720731 -0.376217
O -10.954672 -2.391001 -1.054841
H -9.999255 2.231641 0.885668
CU 0.149898 -0.460450 -0.028919
C 5.669341 -0.442600 -0.081759
C 5.280588 -1.769874 0.142525
C 3.945502 -2.119970 0.159767
C 2.911373 -1.183874 -0.041541
C 3.316675 0.153728 -0.273645
C 4.651236 0.501461 -0.290755
O 2.324061 1.080073 -0.480608
O 1.659117 -1.570553 -0.016629
C 7.046393 0.002662 -0.105799
C 8.146104 -0.746273 0.102966
C 9.496165 -0.244911 0.074102
C 10.590093 -1.007792 0.306577
C 11.919114 -0.439021 0.272122
C 11.985918 0.939848 -0.020968

C	10.855540	1.716985	-0.259977
O	9.609881	1.080298	-0.203558
O	12.938062	-1.156364	0.497175
H	6.030258	-2.539487	0.305704
H	3.650516	-3.151663	0.335613
H	4.918147	1.542136	-0.472230
H	7.183376	1.063982	-0.312204
H	8.069142	-1.809905	0.318733
H	2.719247	1.952350	-0.604710
H	12.949898	1.436956	-0.064153
O	10.787752	2.923908	-0.520937
H	10.463374	-2.062810	0.526333
H	-3.460606	1.759415	1.453941
O	-2.966388	2.783782	-0.281610
H	-3.450084	3.560314	0.023732

ENERGIES
SCF ENERGY: -2025.58021073
SUM OF ELECTRONIC AND ZERO-POINT ENERGIES: -2025.199765
SUM OF ELECTRONIC AND THERMAL ENERGIES: -2025.164471
SUM OF ELECTRONIC AND THERMAL ENTHALPIES: -2025.163527
SUM OF ELECTRONIC AND THERMAL FREE ENERGIES: -2025.274182

==> AOX-II/HIS2-/CU2+/BIS/RAF/C6 <==
55
DATASET

C	5.480865	-0.432513	-0.051250
C	4.855533	-1.711548	0.117004
C	3.508172	-1.822419	0.147247
C	2.647369	-0.681959	0.010679
C	3.279230	0.609878	-0.159582
C	4.629689	0.713982	-0.190662
O	2.431677	1.662818	-0.275775
O	1.396966	-0.837789	0.045429
C	6.840550	-0.255921	-0.077095
C	7.839817	-1.269517	0.088055
C	9.095586	-1.006043	0.934468
C	9.274698	-1.125006	-0.507984
C	9.671545	0.089457	-1.281500
C	9.862869	1.256662	-0.541097
C	9.683835	1.347371	0.849369
O	9.248232	0.222015	1.553649
O	9.834760	-0.021419	-2.519724
H	5.468130	-2.601009	0.218452
H	3.020814	-2.784263	0.272360
H	5.089831	1.691967	-0.316194
H	7.189911	0.767721	-0.218423
H	7.519964	-2.305199	0.146446
H	2.935634	2.482676	-0.365676
H	10.181016	2.163610	-1.045019
O	9.844651	2.340311	1.553544
H	9.662982	-2.065030	-0.890801
CU	-0.064110	0.320788	-0.237372
C	-5.572801	0.397243	-0.222738
C	-5.146061	1.728013	-0.122869
C	-3.806198	2.047090	-0.217373
C	-2.805228	1.074731	-0.413381
C	-3.249684	-0.266341	-0.522264
C	-4.588724	-0.583035	-0.428570
O	-2.290577	-1.227336	-0.728748
O	-1.546623	1.430726	-0.503844
C	-6.955856	-0.017415	-0.123413
C	-8.025201	0.764592	0.119199
C	-9.380885	0.285955	0.211827
C	-10.445978	1.075202	0.486246
C	-11.780601	0.525463	0.576021
C	-11.884913	-0.864458	0.355183
C	-10.784179	-1.668586	0.071149
O	-9.530430	-1.048669	0.006561
O	-12.771935	1.268184	0.839013
H	-5.869795	2.523859	0.031743
H	-3.481040	3.081577	-0.137032
H	-4.886264	-1.627404	-0.517541
H	-7.126563	-1.085481	-0.257059
H	-7.916441	1.836450	0.271209
H	-2.709029	-2.097014	-0.753797
H	-12.855216	-1.348360	0.406038
O	-10.750056	-2.886769	-0.138818
H	-10.291166	2.137202	0.646207
O	9.502126	-1.965576	1.836849
H	9.515407	-2.831429	1.407774

ENERGIES
SCF ENERGY: -2025.58321265
SUM OF ELECTRONIC AND ZERO-POINT ENERGIES: -2025.202100
SUM OF ELECTRONIC AND THERMAL ENERGIES: -2025.167532
SUM OF ELECTRONIC AND THERMAL ENTHALPIES: -2025.166588
SUM OF ELECTRONIC AND THERMAL FREE ENERGIES: -2025.274416

==> AOX-II/HIS2-/CU2+/BIS/RAF/C6B <==
55
DATASET

C	5.369671	-0.148213	-0.357201
C	4.881070	-1.535971	-0.095677
C	3.458154	-1.792739	-0.412383
C	2.553160	-0.843313	-0.780281
C	3.107186	0.493605	-0.946873
C	4.460271	0.803380	-0.725932
O	2.264145	1.407227	-1.342606
O	1.302849	-1.084906	-1.041232
C	6.740365	0.223479	-0.174833
C	7.692081	-0.552545	0.384796
C	9.059073	-0.147615	0.575464
C	9.978070	-0.937195	1.171676
C	11.350354	-0.490667	1.341275
C	11.624049	0.797753	0.846083
C	10.663015	1.603985	0.230001
O	9.366846	1.087567	0.111461
O	12.203342	-1.236260	1.897777
H	3.125818	-2.822024	-0.297506
H	4.777993	1.832331	-0.878081
H	6.997933	1.232014	-0.490609
H	7.459820	-1.549509	0.747795
H	2.682633	2.279493	-1.436180
H	12.625603	1.206766	0.933282
O	10.797502	2.735616	-0.237721
H	9.684038	-1.919560	1.525830
CU	-0.163046	0.030527	-0.661599
C	-5.670714	0.298102	0.033616
C	-5.182632	1.608178	0.130477
C	-3.832219	1.868081	0.019227
C	-2.876600	0.853617	-0.193748
C	-3.381153	-0.467853	-0.284697
C	-4.732428	-0.725351	-0.176688
O	-2.466463	-1.473112	-0.484440
O	-1.608136	1.159245	-0.294100
C	-7.073435	-0.044212	0.134615
C	-8.094565	0.805576	0.357421
C	-9.481394	0.428422	0.443125
C	-10.485305	1.305920	0.678831
C	-11.860848	0.867588	0.750384
C	-12.070854	-0.514580	0.558240
C	-11.032693	-1.410630	0.316526
O	-9.731469	-0.895567	0.266403
O	-12.795040	1.693451	0.972471
H	-5.867702	2.436230	0.291368
H	-3.461416	2.887702	0.092286
H	-5.075163	-1.756516	-0.256546
H	-7.303111	-1.102828	0.014343
H	-7.915766	1.870648	0.489953

H -2.927217 -2.320016 -0.531433
H -13.077652 -0.918536 0.596711
O -11.094437 -2.632135 0.133636
H -10.249131 2.356180 0.815429
H 4.961216 -1.635681 1.009696
O 5.741910 -2.471741 -0.722809
H 5.471968 -3.357897 -0.451353

ENERGIES
SCF ENERGY: -2025.56749565
SUM OF ELECTRONIC AND ZERO-POINT ENERGIES: -2025.187102
SUM OF ELECTRONIC AND THERMAL ENERGIES: -2025.151888
SUM OF ELECTRONIC AND THERMAL ENTHALPIES: -2025.150944
SUM OF ELECTRONIC AND THERMAL FREE ENERGIES: -2025.263181

==> AOX-II/HIS2-/CU2+/MONO/BIDENTATE <==
33
DATASET
C 0.362780 -0.299376 -0.010569
C -0.050884 -1.637980 0.057001
C -1.387125 -1.983184 0.055846
C -2.382032 -0.999074 -0.009123
C -1.957406 0.335631 -0.091628
C -0.629280 0.691573 -0.088605
O -2.998968 1.252216 -0.217030
O -3.668359 -1.292474 0.015190
C 1.760384 0.094999 -0.011002
C 2.815399 -0.738851 -0.018814
C 4.200291 -0.341166 -0.007897
C 5.225545 -1.222963 -0.031953
C 6.600612 -0.773836 -0.015997
C 6.781757 0.624215 0.024828
C 5.718987 1.525292 0.050259
O 4.420912 0.997887 0.030742
O 7.552523 -1.607873 -0.036857
H 0.688883 -2.430505 0.121373
H -1.694996 -3.022876 0.120805
H -0.352870 1.741948 -0.150326
H 1.944533 1.168864 -0.007917
H 2.672067 -1.817194 -0.036026
H -2.821805 2.081824 0.247541
H 7.784740 1.038912 0.041232
O 5.756121 2.759877 0.089457
H 5.009377 -2.285859 -0.062278
CU -4.809860 0.223692 -0.010514
O -6.419073 -1.029615 0.179664
H -7.178014 -0.676884 -0.303474
O -5.964142 1.864488 -0.026624
H -6.696382 1.756057 0.595096
H -6.201887 -1.868194 -0.249131
H -5.453972 2.619112 0.297607

ENERGIES
SCF ENERGY: -1226.31974417
SUM OF ELECTRONIC AND ZERO-POINT ENERGIES: -1226.085985
SUM OF ELECTRONIC AND THERMAL ENERGIES: -1226.064169
SUM OF ELECTRONIC AND THERMAL ENTHALPIES: -1226.063225
SUM OF ELECTRONIC AND THERMAL FREE ENERGIES: -1226.138292

==> AOX-II/HIS2-/CU2+/MONO/UNIDENTATE/C3P <==
36
DATASET
C 0.639583 0.352373 0.219484
C 0.179934 1.675135 0.192079
C -1.172670 1.955955 0.249898
C -2.138621 0.942359 0.352768
C -1.665802 -0.388115 0.381658
C -0.317726 -0.668601 0.315389
O -2.618047 -1.368729 0.484167
O -3.424630 1.231579 0.437523
C 2.039181 -0.020659 0.155910
C 3.096059 0.804829 0.052904
C 4.468479 0.366688 -0.009614
C 5.523764 1.205837 -0.116533
C 6.878454 0.700403 -0.177318
C 7.008202 -0.702915 -0.116334
C 5.915238 -1.559379 -0.004057
O 4.641872 -0.979057 0.046351
O 7.860267 1.492922 -0.278705
H 0.886712 2.497021 0.118679
H -1.524012 2.984165 0.224142
H 0.003834 -1.708768 0.342260
H 2.230882 -1.092461 0.195717
H 2.967012 1.884187 0.007926
H -2.203434 -2.239417 0.439998
H 7.993329 -1.157010 -0.156064
O 5.905034 -2.793763 0.060232
H 5.348174 2.275738 -0.157928
CU -4.761475 0.003684 -0.164898
O -4.086122 0.091532 -2.110502
H -4.830397 0.109881 -2.726642
O -6.332722 -1.119244 -0.739365
H -6.164683 -1.566508 -1.579513
H -3.620776 0.928791 -2.240478
H -6.507445 -1.820857 -0.097957
O -5.426334 -0.247732 1.763162
H -6.375242 -0.079037 1.831797
H -5.000175 0.433904 2.299784

ENERGIES
SCF ENERGY: -1302.75971402
SUM OF ELECTRONIC AND ZERO-POINT ENERGIES: -1302.500939
SUM OF ELECTRONIC AND THERMAL ENERGIES: -1302.477022
SUM OF ELECTRONIC AND THERMAL ENTHALPIES: -1302.476078
SUM OF ELECTRONIC AND THERMAL FREE ENERGIES: -1302.554638

==> AOX-II/HIS2-/CU2+/MONO/UNIDENTATE/C4P <==
36
DATASET
C -0.602855 0.107972 -0.421298
C -0.018302 -1.143111 -0.662123
C 1.338011 -1.251439 -0.894524
C 2.184434 -0.130125 -0.914619
C 1.587641 1.122833 -0.653563
C 0.232994 1.233835 -0.415851
O 2.412353 2.217920 -0.671951
O 3.468491 -0.232082 -1.203187
C -2.023255 0.298924 -0.193669
C -2.974460 -0.650588 -0.227700
C -4.379655 -0.413252 -0.007422
C -5.319672 -1.383861 -0.055478
C -6.717161 -1.083008 0.173986
C -7.011526 0.270525 0.437820
C -6.035612 1.263954 0.482479
O -4.708910 0.877561 0.254463
O -7.591705 -1.996502 0.133699
H -0.629415 -2.040941 -0.667363
H 1.788485 -2.221338 -1.091270
H -0.190944 2.219943 -0.231987
H -2.325881 1.324726 0.012755
H -2.726782 -1.688473 -0.439219
H 1.909712 3.014831 -0.457973
H -8.037906 0.574674 0.617455
O -6.174608 2.472415 0.700207
H -5.018274 -2.404279 -0.268018
CU 4.766164 -0.249829 0.217555
O 6.210283 -0.257494 1.638558
H 6.053863 0.435979 2.293137
H 6.228716 -1.084926 2.136473

O	5.052609	1.738256	-0.020245
H	5.306497	2.163886	0.808268
O	4.390742	-2.226478	0.530199
H	5.195107	-2.726984	0.720151
H	4.154133	2.067060	-0.232899
H	4.036411	-2.593848	-0.290774

ENERGIES
SCF ENERGY: -1302.76228838
SUM OF ELECTRONIC AND ZERO-POINT ENERGIES: -1302.501580
SUM OF ELECTRONIC AND THERMAL ENERGIES: -1302.477799
SUM OF ELECTRONIC AND THERMAL ENTHALPIES: -1302.476855
SUM OF ELECTRONIC AND THERMAL FREE ENERGIES: -1302.555876
